# Burdens of Breast Cancer and Projections for 2030 Among Women in Asia: Findings from the 2021 Global Burden of Disease Study

**DOI:** 10.3390/curroncol32050267

**Published:** 2025-05-01

**Authors:** Feng Wang, Sixuan Liu, Jianwei Li, Yuzhen Shi, Zhaohui Geng, Yajie Ji, Jie Zheng

**Affiliations:** 1 Shuguang Hospital Affiliated to Shanghai University of Traditional Chinese Medicine, Shanghai 201203, China; ghio96678@163.com (F.W.);; 2 Fudan University Shanghai Cancer Center, Shanghai 200032, China; 3 Department of Nursing, Shanghai University of Traditional Chinese, Shanghai 201203, China

**Keywords:** global burden of disease, breast cancer, Asia, age–period–cohort model, autoregressive integrated moving average model

## Abstract

***Background*:** Employing the most recent dataset from the Global Burden of Disease (GBD) Study 2021, this report sought to delineate the current epidemiologic landscape of breast cancer in Asian women. ***Methods*:** We examined the evolving trends in disease prevalence and explored the correlations between breast cancer and factors such as age, temporal periods, and generational cohorts. We utilized an autoregressive integrated moving average (ARIMA) model to predict the incidence and deaths of breast cancer in Asia. ***Results*:** From 1990 to 2021, the age-standardized incidence rate (ASIR), age-standardized DALYs rate (ASDR), and age-standardized mortality rate showed an overall upward trend for Asian women with breast cancer. In 2021, the high-income Asia Pacific region had the highest ASIR value, while South Asia had the lowest ASIR value. The highest age-standardized mortality rate and ASDR values in 2021 occurred in Southeast Asia, while the lowest values for these metrics were in East Asia. In 2021, breast cancer incidence and DALYs were highest in the 50–54 age group, with deaths peaking in the 55–59 age group. The leading risk factor attributed to breast cancer deaths in Asia in 1990 and 2021 was a “diet high in red meat”. Breast cancer incidence and mortality rates are expected to continue to rise in Asia over the next 10 years. ***Conclusions*:** The burden of breast cancer in Asian women is increasing, especially in low SDI countries. This study highlighted the differences between populations and regions and predicted the incidence and mortality rates of breast cancer in Asia over the next decade using an ARIMA model. An increased awareness of breast cancer risk factors and prevention strategies is necessary to reduce breast cancer burden in the future.

## 1. Background

Breast cancer remains the leading cancer type affecting women worldwide, consistently ranking among the top three most diagnosed cancers globally alongside lung and colorectal cancers. According to 2012 global cancer statistics, this disease accounted for about 1.7 million new diagnoses and was associated with half a million cancer-related deaths that year [1,2]. Breast cancer mortality rates have exhibited a downward trend in both North America and the European Union, primarily due to advancements in early detection and the effectiveness of systemic therapies. However, in South America, Africa, and Asia, breast cancer incidence is rising, likely due to shifts in lifestyle patterns and the introduction of screening initiatives. Breast cancer mortality rates continue to increase in these regions, partly because of limited access to advanced diagnostic technologies and effective treatment options [2].

Although the incidence of invasive breast cancer is lower in Asian compared with Western women, a significant increase in breast cancer incidence is being currently observed in several Asian countries [3]. In Iran, for example, the annual incidence of breast cancer is about 20 new cases per 100,000 women [4], and among these patients, 70% are diagnosed at an advanced stage of cancer, resulting in death within a short period of time [5]. Moreover, Asians make up 60% of the world population (7 billion people), and that number is growing rapidly. China and India alone, the two most populous countries, account for 37% of the world population [6]. The burden of breast cancer in Asia is closely tied to factors such as racial diversity, cultural perspectives, healthcare systems, geographic distribution, and the pace of urbanization. Investigating the disease burden of breast cancer among Asian women holds significant importance for understanding and improving the physiological health of women in Asia and worldwide.

This study aims to comprehensively analyze trends in breast cancer incidence, disability-adjusted life years (DALYs), and mortality in Asia. By stratifying the data by age and sociodemographic index (SDI), and breast cancer-related risk factors, this study highlights differences between populations and regions, and predicts the incidence and mortality of breast cancer in Asia over the next decade by using an autoregressive integrated moving average (ARIMA) model. These findings offer evidence-based guidance for health strategies to policymakers and public health practitioners, informing future breast cancer control initiatives.

## 2. Methods

### 2.1. Data Sources

The Global Burden of Disease (GBD) Study 2021 provides a comprehensive and standardized methodology for estimating mortality and morbidity by cause and age across the globe. This large-scale project synthesizes epidemiological data from 204 countries and territories, facilitating a comparative analysis of health burdens associated with 369 diseases and injuries, alongside 88 risk factors [7]. Breast cancer data come from vital registries, police records, surveillance, surveys/censuses, and other health-related data sources that can provide partial or full information on breast cancer incidence and deaths [8].

In this study, we used the Global Health Data Exchange query tool to search the GBD Study 2021 database for institutional assessment site, health indicators, and input data source tools (https://vizhub.healthdata.org/gbd-results/) (accessed on 6 January 2025) to retrieve data about breast cancer incidence, mortality, and DALYs.

This study conducted GBD database analyses in full compliance with the cross-sectional methodological standards defined in the Guidelines for Accurate and Transparent Health Estimates Reporting [9]. The University of Washington Institutional Review Board (USA) issued approval for the informed consent exemption requirement in GBD research. This work was reported in accordance with STROCSS standards [10].

### 2.2. SDI

SDI quantifies the developmental status of a country or region by integrating data on fertility rates, educational attainment, and per capita income. Ranging from 0 to 1 on a numerical scale, a higher SDI value reflects more advanced socioeconomic development [3]. In GBD 2021, after computing the sociodemographic index (SDI), the value is scaled by multiplying it by 100, resulting in a range from 0 to 100. A score of 0 corresponds to the lowest levels of income and education, coupled with the highest fertility rates, while a score of 100 represents the highest levels of income and education, along with the lowest fertility rates [11].

### 2.3. Data Analysis

A descriptive analysis of time and age trends in the burden of breast cancer in Asia was conducted and compared with global trends. This analysis was performed using R software package version 4.4.2 (R Foundation, Vienna, Austria).

#### 2.3.1. Age-Standardized Rate (ASR) and Estimated Annual Percentage Change (EAPC)

The burden of non-fatal diseases in the GBD study was modeled through Bayesian meta-regression analysis [12]. ASR was computed as a weighted average of age-specific ratios, derived through the summation of hypothetical ratio values under a standardized population age distribution.

EAPC is a valid and widely used indicator from previous studies that tracks trends in measures such as prevalence and morbidity over a specific time period [13]. The calculation of EAPC is based on the natural logarithm rate of a fitted regression model. The natural logarithm of each observed value is fitted onto a straight line with time as the variable and calculated according to the slope of this line [14]: $EAPC=ln\left(ASR\right)=\alpha +\beta x+\varepsilon$, where *x* denotes the calendar year, and ε denotes the error term, whose 95% confidence interval (CI) is established utilizing the formula 100 × (exp (β) − 1) [15].

#### 2.3.2. Analysis of Cross-Country Inequalities

The slope index of inequality (SII) and the concentration index were employed to assess socioeconomic disparity in the burden of breast cancer, as measured by DALYs, across Asian countries and regions. These metrics, endorsed by the World Health Organization, serve as standardized measures for evaluating absolute and relative gradient inequalities [16]. The SII was derived through a national rate regression model, utilizing the SDI correlation scale, where the midpoint of the cumulative population, ranked by SDI, was used as the reference. Heteroscedasticity was addressed by applying a weighted regression model. The concentration index was calculated by measuring the area under the Lorenz curve, which was constructed using the relationship between SDI rankings and the cumulative population distribution [17]. These indices evaluate absolute and relative inequalities independently, with values deviating further from zero signifying higher levels of disparity. A positive SII or concentration index suggests a greater burden in countries with higher SDI, while negative values indicate a higher burden in nations with lower SDI.

#### 2.3.3. Age–Period–Cohort Model (APC Model)

The APC model was employed to assess associations among age, period, birth cohort, and breast cancer incidence. The birth cohort of a person can be calculated by the time period of diagnosis and their age (birth cohort = period of diagnosis − age) [18]. Period effects capture temporal shifts that simultaneously influence all age groups, likely stemming from transformations in social, economic, cultural, or environmental conditions. Cohort effects denote differential trajectories among populations sharing birth years [19]. Period and cohort relative risks (RRs) measure age-stratified rate ratios versus a reference population. The age curve reflects the expected age-specific rates in a reference cohort, adjusted for period-related influences. In age–period–cohort (APC) models, net drift and local drift are critical parameters: net drift captures the overall log-linear trend across periods and birth cohorts, representing the mean annual percentage change in age-adjusted rates over time. Local drift, on the other hand, describes the log-linear trend for each age group across periods and birth cohorts, indicating the annual percentage change in age-specific rates over time.

#### 2.3.4. ARIMA Model

The ARIMA model, a well-established approach in time-series analysis, integrates autoregressive (AR), differencing (I), and moving average (MA) elements to effectively identify trends and cyclical variations within sequential data. This model enables the projection of future trends and patterns [20]. In the ARIMA model, the time-series must be a stationary random sequence with a zero mean value. Initially, the Augmented Dickey–Fuller (ADF) test is employed to assess the stationarity of the series. If the ADF test yields significant results, the series is confirmed to be stationary. Subsequently, the parameters of the ARIMA model are preliminarily estimated using autocorrelation function (ACF) and partial autocorrelation function (PACF) plots. The adequacy of the residuals of the model is evaluated through the Ljung–Box Q test, along with ACF and PACF analyses, to ensure that they conform to the properties of a white noise sequence. Once the model is validated and passes the white noise test, it is utilized to forecast the incidence and mortality rates of breast cancer in Asia from 2022 to 2031.

## 3. Results

### 3.1. Overall Trends in Breast Cancer Incidence, Mortality, and DALYs Burden Among Asian Women

#### 3.1.1. Overall Breast Cancer Trends

The number of women with breast cancer in Asia in 2021 (95% uncertainty interval [UI]: 934,358.23 (837,386.96 to 1,050,748.44)) (Table 1), and the percentage change in age-standardized incidence rate (ASIR) (95% UI: 84.85% (59.08% to 115.61%)) have increased significantly during the 1990–2021 study period (Table 2). In Asia in 2021, the estimated breast cancer-related deaths (95% UI: 306,494.59 (276,354.3 to 340,676.9)) (Table 3) represent an increase from 1980 (95% UI: 16.9% (3.83% to 31.97%)) (Table 2). The value of DALYs has also increased from 1990 (95% UI: 3,993,737.13 (3,554,041.8 to 4,499,412.85)) to 2021 (95% UI: 10,335,176.26 (9,280,423.16 to 11,485,399.63)) (Table 4), representing a considerable increase (95% UI: 17.31% (2.72% to 32.8%)) (Table 2). EAPC in ASIR (95% UI: 199.09% (194.95% to 203.23%)) (Table 1), EAPC in age-standardized mortality rate (95% UI: 44.39% (40.05% to 48.73%)) (Table 3), and EAPC in age-standardized DALYs rate (ASDR) (95% UI: 38.88% (32.52% to 45.25%)) also showed an increasing trend (Table 4).

#### 3.1.2. Breast Cancer Trends by Geographic Region

**Figure 1:**
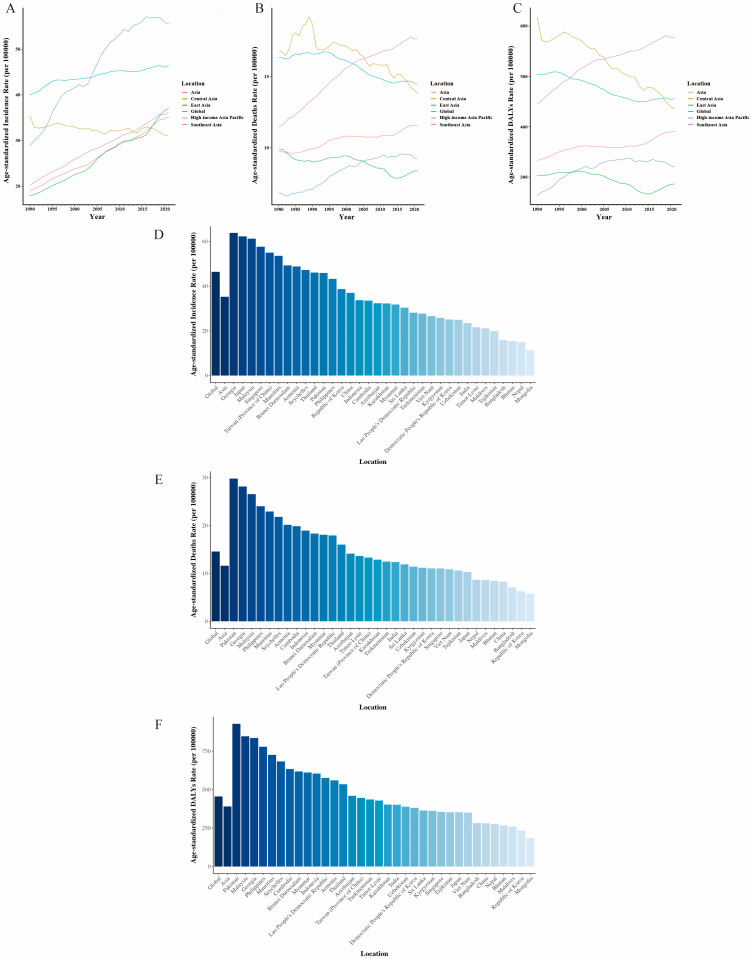
ASIR, ASDR, and age-standardized death rate that related to breast cancer in geographical regions and countries in Asia from 1990 to 2021. (**A**) Changing trends of ASIR in geographical regions in Asia from 1990 to 2021. (**B**) Changing trends of age-standardized death rate in regions in Asia from 1990 to 2021. (**C**) Changing trends of ASDR in regions in Asia from 1990 to 2021. (**D**) ASIR related to breast cancer in countries and territories in Asia and global in 2021. (**E**) Age-standardized death rate related to breast cancer in countries and territories in Asia and global in 2021. (**F**) ASDR related to breast cancer in countries and territories in Asia and global in 2021.

Breast cancer incidence, deaths, and DALYs were on the rise in all Asian geographic regions during the study period. Among them, the region with the largest ASIR value in 2021 was the high-income Asia Pacific region (95% UI: 5562.38% (5048.57% to 5945.23%)), while the region with the lowest ASRI was South Asia (95% UI: 2462.48% (2151.53% to 2832.02%)) (Figure 1A, Table 1). Additionally, the region with the highest age-standardized mortality rate and ASDR in 2021 was Southeast Asia (95% UI: 1772.42% (1476.27% to 2151.5%)), and (95% UI: 57,797.87% (47,690.73% to 70,699.18%)), respectively (Figure 1B, Table 3), whole those with the lowest age-standardized mortality rate and ASDR in 2021 was East Asia (95% UI: 837.98% (659.5% to 1039.86%)), and (95% UI: 28,625.16% (22,385.77% to 36,040.98%)), respectively (Figure 1C, Table 4). Between 1990 and 2021, the percentage change in ASIR increased the most in East Asia (95% UI: 107.2% (50.33% to 189.58%)), while among all regions in Asia, only Central Asia (95% UI: −12.5% (−22.56% to −0.96%)) showed a downward trend (Table 2). During the study period from 990 to 2021, the percentage change in age-standardized mortality rate was the fastest in South Asia (95% UI: 40.82% (19.34% to 66.4%)), while Central Asia showed the largest decrease (95% UI: −25.82% (−34.07% to −16.89%)) (Table 2). The percentage change in ASDR was the fastest in South Asia (95% UI: 38.67% (18.04% to 63.43%)), while Central Asia showed the largest decline (95% UI: −29.64% (−38.08% to −20.4%)) (Table 2).

With the exception of Central Asia (95% UI: −18.97% (−26.04% to −11.89%)), EAPC in ASIR was on the rise in the remaining Asian regions, with the fastest growth observed in East Asia (95% UI: 234.92% (227.01% to 242.84%)) (Table 1). Although EAPC in age-standardized mortality rate decreased in Central Asia (95% UI: −53.89% (−64.45% to −43.32%)) and East Asia (95% UI: −43.11% (−51.55% to −34.67%)), it showed an upward trend in other Asian regions, and the fastest increase was in South Asia (95% UI: 109.39% (101.82% to 116.96%)) (Table 3). Similarly to the EAPC in age-standardized mortality rate, the EAPC in ASDR decreased in Central Asia (95% UI: −99.5% (−108.5% to −90.5%)) and East Asia (95% UI: −45.78% (−56.76% to −34.79%)), while it showed an upward trend in other Asian regions, with the fastest increase in South Asia (95% UI: 96.45% (83.86% to 109.05%)) (Table 4).

#### 3.1.3. Breast Cancer Trends by Asian Country

Among Asian countries in 2021, China had the highest incidence of breast cancer (95% UI: 385,837.7 (294,095.4 to 489,009.76)) (Table 1) and the highest number of breast cancer-related deaths (95% UI: 88,106.72 (68,162.64 to 110,341.23)) (Table 3) and DALYs (95% UI: 2,921,096.2 (2,254,510.09 to 3,716,738.57)) (Table 4). In contrast, Seychelles in 2021 exhibited the lowest values for breast cancer incidence (95% UI: 28.92 (24.48 to 34.01)) (Table 1), breast cancer-related deaths (95% UI: 13.28 (11.2 to 15.48)) (Table 3), and DALYs (95% UI: 420.54 (354.63 to 493.04)) (Table 4). Georgia displayed the highest ASIR value (95% UI: 6386.47% (5482.06% to 7435.85%)) in 2021 (Figure 1D, Table 1) and Mongolia exhibited the lowest ASIR value (95% UI: 1136.86% (874.4% to 1412.04%)) (Figure 1D, Table 1). In 2021, Pakistan was the country with the highest age-standardized mortality rate (95% UI: 2976.42% (2158.15% to 3907.74%)) (Figure 1E, Table 3) and Mongolia was the country with the lowest age-standardized mortality rate (95% UI: 575.73% (439.33% to 710.15%)) (Figure 1E, Table 3). Similarly, in 2021, Pakistan had the highest ASDR (95% UI: 92,737.36% (66,590.36% to 123,896.93%)) (Figure 1F, Table 4), while Mongolia had the lowest ASDR (95% UI: 18,522.31 (14,419.27% to 22,716.59%)) (Figure 1F, Table 4).

Between 1990 and 2021, the percentage change in ASIR increased in most Asian countries, with the largest increase observed in the Republic of Korea (95% UI: 205.26% (129.31% to 274.16%)), while the most significant decline was in Kazakhstan (95% UI: −18.16% (−32.86% to 0.48%)) (Table 2). The percentage change in age-standardized mortality rate was on the rise in most Asian countries, and the rise was most obvious in Mauritius (95% UI: 96.68% (72.91% to 116.7%)), while the highest decline was noted in Kazakhstan (95% UI: −38.92% (−50.13% to −25.06%)) (Table 2). As is consistent with the percentage change in age-standardized mortality rate, the percentage change in ASDR was also the highest in Mauritius (95% UI: −99.41% (73.93% to 121.45%)). Most other Asian countries also showed a general upward trend, with Kazakhstan (95% UI: −41.33% (−52.01% to −28.57%)) showing the largest decline among the few countries showing a downward trend (Table 2).

The country with the highest EAPC in ASIR was the Republic of Korea (95% UI: 396.83% (361.24% to 432.53%)) and the lowest was Kyrgyzstan (95% UI: −77.58% (−102.18% to −52.91%)) (Table 1). The country with the highest EAPC in age-standardized mortality rate was Mauritius (95% UI: 184.07% (151.8% to 216.43%)) and the country with the lowest such rate was Kyrgyzstan (95% UI: −127.18% (−142.4% to −111.95%)) (Table 3). The country with the highest EAPC in ASDR was Mauritius (95% UI: 160.98% (128.32% to 193.74%)), while the country with the lowest EAPC in ASDR was Kyrgyzstan (95% UI: −176.54% (−192.33% to 160.72%)) (Table 4).

### 3.2. Age Differences and Corresponding Trends

**Figure 2:**
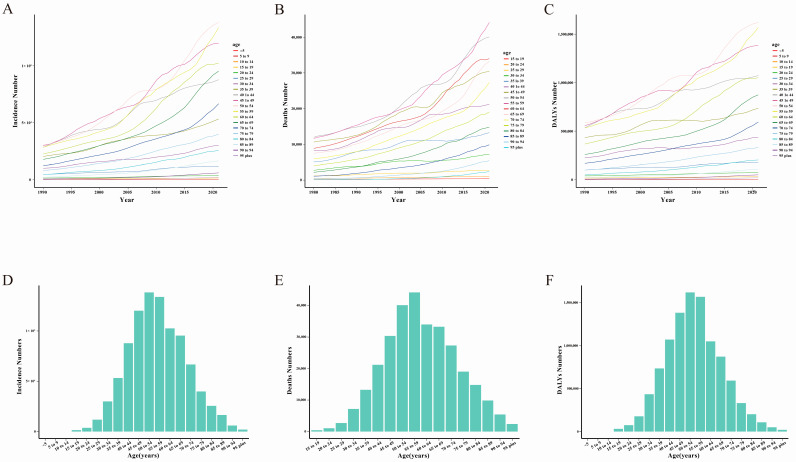
The disease burden attributed to breast cancer in different age groups in Asia. (**A**) The incidence number ratio in Asia by age groups from 1990 to 2021. (**B**) The death number ratio in Asia by age groups from 1990 to 2021. (**C**) The DALYs number ratio in Asia by age groups from 1990 to 2021. (**D**) The incidence number associated with breast cancer in Asians by age groups in 2021. (**E**) The death number associated with breast cancer in Asians by age groups in 2021. (**F**) The DALYs number associated with breast cancer in Asians by age groups in 2021.

From 1990 to 2021, the incidence of breast cancer in each age group (≥15–19 years old) has shown an overall upward trend. The 60–64 age group showed a rapid upward trend after 2005 (Figure 2A, Supplementary Table S1). The changing DALYs trend for each age group from 1990 to 2021 was similar to that of breast cancer incidence (Figure 2C, Supplementary Table S2). From 1980 to 2021, the number of deaths in all age groups (≥15–19 years of age) continued to show an overall increase, especially in the 50–54 year, 55–59 year, and 60–64 year age groups. The 65–69 year age group showed a rapid upward trend after 2000 (Figure 2B, Supplementary Table S3).

In 2021, breast cancer incidence values in Asia in the 15–19 year and 50–54 year age groups reached a peak (95% UI: 138,208.1 (121,113.36 to 159,620.02)) and then showed a downward trend (Figure 2D, Supplementary Table S1). Breast cancer-related deaths reached a peak in 2021 in the 55–59 year age group (95% UI: 44,131.81 (37,991.78 to 50,971.79)) and the change pattern of this bar chart is similar to that of breast cancer incidence (Figure 2E, Supplementary Table S3). The DALYs value for the 50–54 year age group (95% UI: 1,620,795.89 (1,436,581.53 to 1,831,453.24)) peaked in 2021, and then showed a downward trend (Figure 2F, Supplementary Table S2).

### 3.3. Breast Cancer Burden in Asia with Reference to Risk Factors

**Figure 3:**
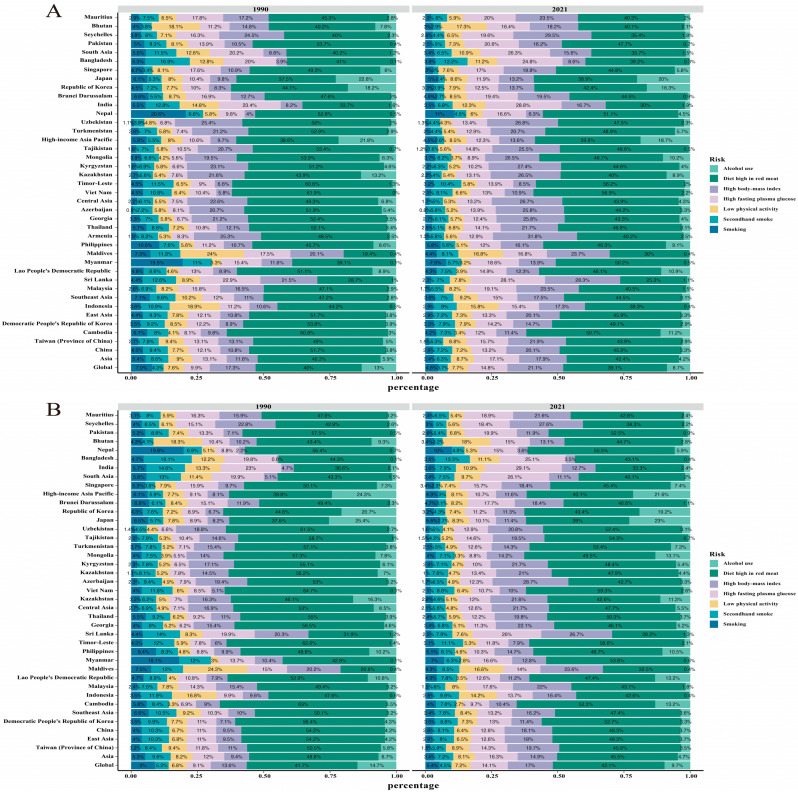
Asia breast cancer in ASDR and age-standardized death rate and its proportion by etiologies. (**A**) Asia breast cancer in age-standardized death rate and its proportion by etiologies in 1990 and 2021. (**B**) Asia breast cancer in ASDR and its proportion by etiologies in 1990 and 2021.

Our findings in the Asian women cohort regarding the burden of breast cancer attributable to risk factors are presented in Figure 3A. In both 1990 and 2021, the primary risk factor contributing to breast cancer-related deaths in Asia was a “diet high in red meat”, aligning with the leading risk factor globally for breast cancer mortality. In 2021, “high body-mass index” and “high fasting plasma glucose” ranked as the second and third leading risk factors for breast cancer-related deaths in Asia, respectively. Among Asian countries, Vietnam had the highest proportion of breast cancer deaths attributed to a “diet high in red meat” (56.9%) in 2021. Notably, “alcohol use” was not identified as a risk factor for breast cancer deaths in Vietnam in 1990, but by 2021, it accounted for 2.2% of these deaths. Conversely, Sri Lanka had the lowest proportion of breast cancer deaths linked to a “diet high in red meat” (25.3%) in 2021. In Sri Lanka, “high body-mass index” (28.3%) and “high fasting plasma glucose” (28.1%) emerged as the primary risk factors for breast cancer mortality, both surpassing the contribution of a “diet high in red meat.”

The main risk factor attributed to breast cancer DALYs in Asia in 1990 and 2021 was still “diet high in red meat”, which was consistent with the main risk factor attribution for breast cancer DALYs globally. The Asian countries reporting the highest and lowest proportion of “diet high in red meat” among the risk factors for breast cancer DALYs in 2021 were East Timor (58.6%) and Sri Lanka (28.2%), respectively (Figure 3B).

### 3.4. Breast Cancer Burden Inequalities Between Countries

**Figure 4:**
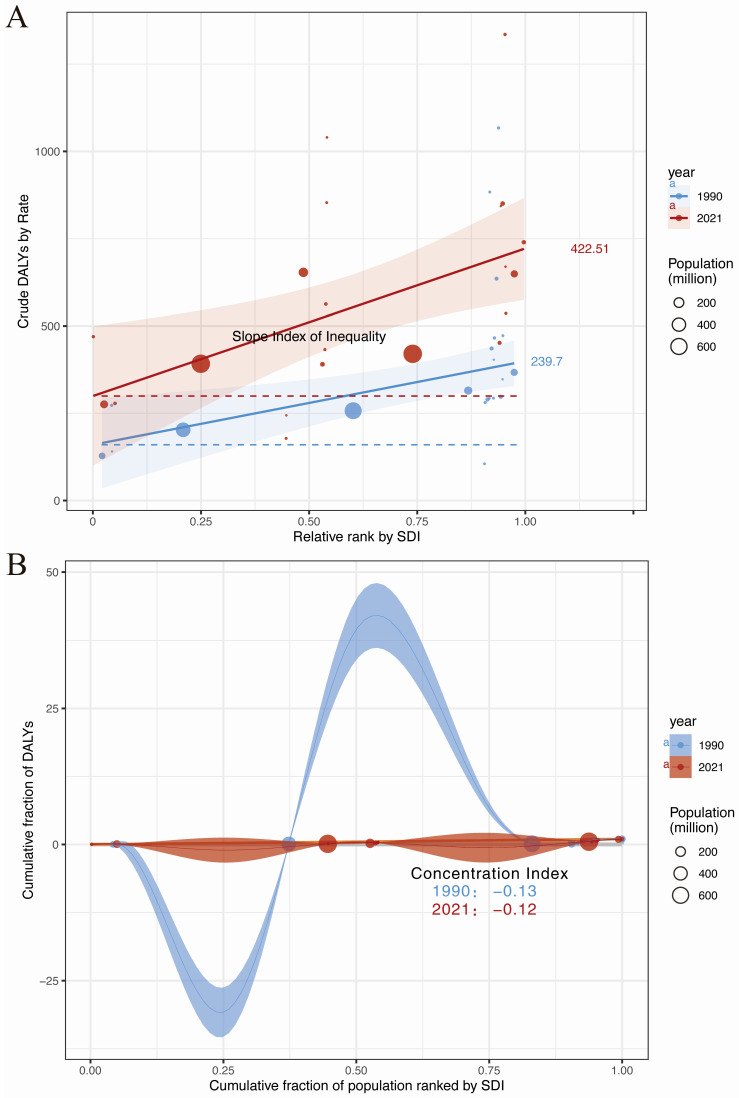
Absolute and relative cross-country inequality for ASDR of Asian breast cancer, 1990–2021. (**A**) Health inequality regression curves for ASDR of Asian breast cancer. (**B**) Concentration curves for ASDR of Asian breast cancer.

Significant absolute and relative SDI-related inequalities were observed in the burden of breast cancer DALYs in Asia, and these inequalities increased significantly over time (Figure 4). As indicated by the slope index of inequality, the gap in breast cancer DALYs rates between countries with the highest and lowest SDI increased from 239.70 (95% CI: 76.29 to 403.10) in 1990 to 422.51 (95 % CI: 112.56 to 732.45) in 2021 (Figure 4A). In addition, the concentration index (a measure of relative gradient inequality) was −0.13 (95% CI: −0.14 to −0.12) in 1990 and −0.12 (95% CI: −0.13 to −0.11) in 2021, indicating that the burden is unevenly distributed among countries with different SDI (Figure 4B).

### 3.5. Age–Period–Cohort Analysis

**Figure 5:**
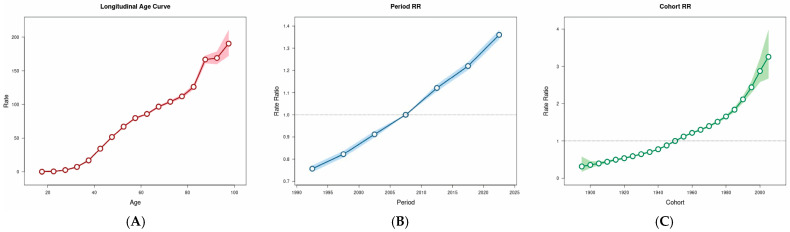
Age–period–cohort impact of burden for ASIR of Asian breast cancer. Red, blue, and green denote age, period, and birth cohort factors, respectively. (**A**) Longitudinal age curve. (**B**) Period RR. (**C**) Cohort RR.

We used the APC model to estimate the effects of age, period, and cohort on Asian breast cancer ASIR (Figure 5, Supplementary Table S4). When cycle and cohort effects were controlled, the incidence rates of breast cancer in Asia showed an overall upward trend with increasing age. In the period effect, using the period group from 2005 to 2010 as the reference value (relative risk [RR] = 1), the ASIR of Asian breast cancer showed a monotonous increase during the whole study period. In terms of cohort effects, using the 1945–1950 birth cohort as a reference value (RR = 1), we found that Asian breast cancer ASIR increased with the birth cohort.

### 3.6. Frontier Analysis for the Association Between Ideal Breast Cancer DALYs and SDI

**Figure 6:**
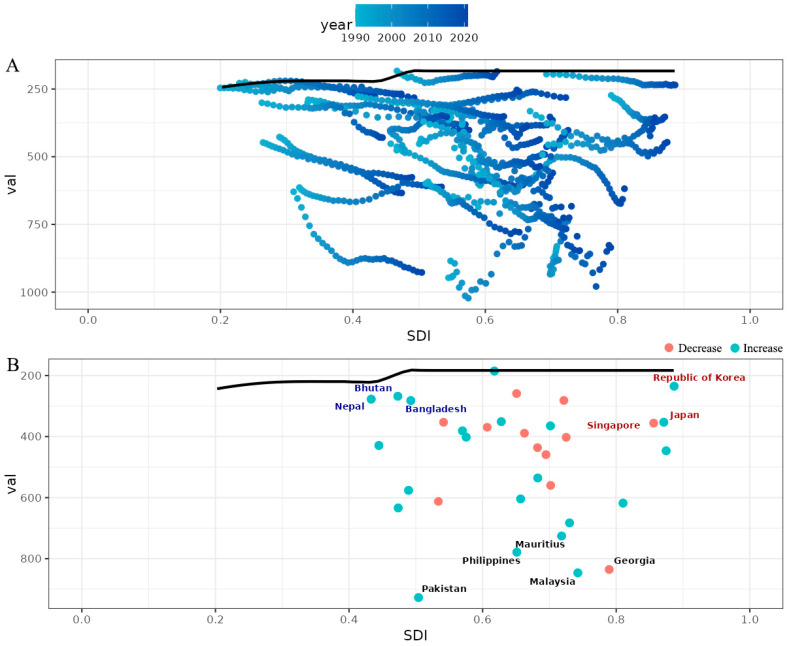
A frontier analysis on the basis of the sociodemographic index and ASDR (per 100,000) of breast cancer in Asia from 1990 to 2021. (**A**) The frontier line is delineated in black, indicating the potentially achievable age-standardized DALYs on the basis of sociodemographic index; the dots represent the actual age-standardized DALYs in every country and territory. The color scale represents the years from 1990 depicted in light blue to 2021 in dark blue. (**B**) A frontier analysis on the basis of the sociodemographic index and age-standardized DALYs per 100,000 of Asian breast cancer in 2021. The increase in ASDR from 1990 to 2021 is shown in green dots, whereas the decrease in red dots. The frontier line, representing the potentially achievable ASDR on the basis of the sociodemographic index is portrayed in black. The top five countries with the highest effective difference are labeled in black; the top three countries with the lowest effective difference in low sociodemographic index (<50) are labeled in blue, whereas the top three countries with the highest effective difference in high sociodemographic index (>85) are labeled in red.

Frontier analysis was conducted to explore the ideal situation in which Asian countries would be able to control the disease burden under corresponding annual SDI conditions (Figure 6, Supplementary Table S5). In the frontier analysis results, the three countries closest to the border fitting line in the lower SDI countries are marked in blue (Figure 6; Bhutan, Nepal, Bangladesh, East Timor). In the higher SDI countries, the three countries farthest from the border fitting line are marked in red (Republic of Korea, Japan, Singapore), and among all the countries, the five countries farthest from the border fitting line are marked in black (Pakistan, Malaysia, Philippines, Georgia, Mauritius).

### 3.7. Projections of Breast Cancer Incidence and Mortality from 2020 to 2031

**Figure 7:**
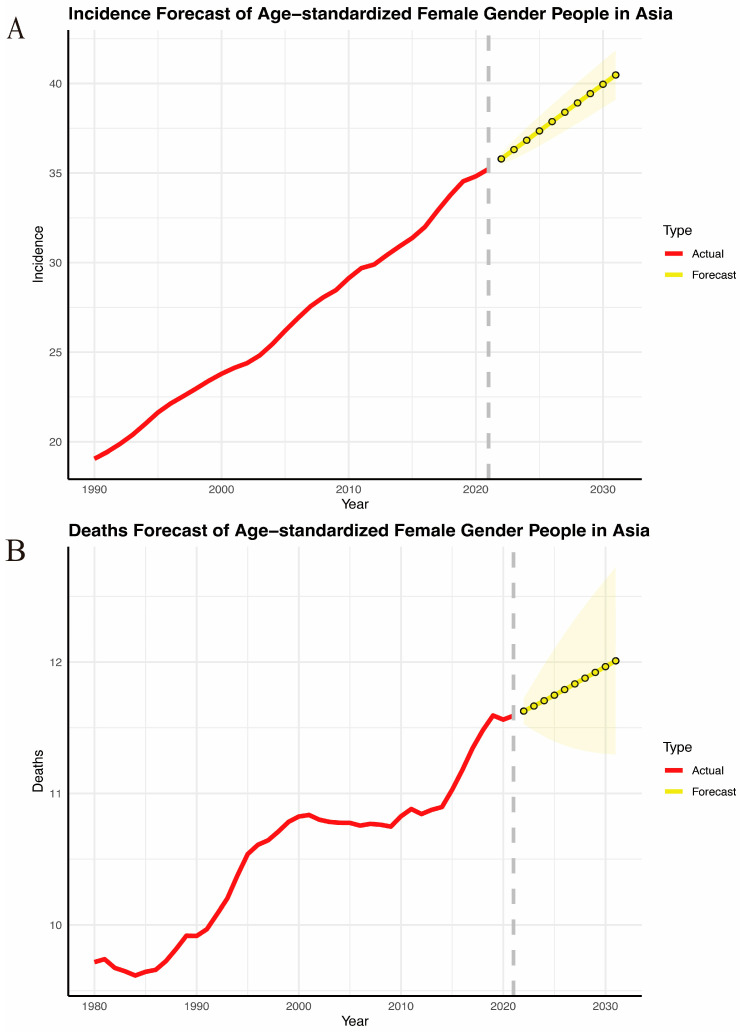
Burden of breast cancer in Asia between 1990 and 2030. (**A**) Incidence. (**B**) Deaths.

The ARIMA model analysis revealed the results for breast cancer incidence and mortality in Asia. Overall, the incidence of breast cancer has continued to increase over the past three decades and is expected to remain on the rise over the next 10 years, reaching 40.48 (95% CI: 39.10–41.85) by 2031 (Figure 7A). Meanwhile, breast cancer deaths in Asia showed an overall upward trend from 1980 to 2021 and are projected to reach 12.01 (95% CI: 11.30–12.72) in 2031 (Figure 7B).

## 4. Discussion

As is consistent with a global rise in breast cancer incidence over the past several decades [21,22,23], Asian countries have seen the greatest increase in this parameter [24]. Our present study revealed a consistently upward trend in ASIR, ASDR, and the age-standardized mortality rate of breast cancer among Asian women between 1990 and 2021. Lifestyle and environmental shifts are likely significant contributors to the pathogenesis of breast cancer in this population. Potential drivers for the rapid rise in breast cancer incidence in Asia include the following: (1) dietary factors, such as high fat consumption, low vegetable intake, and reduced soy consumption, and (2) reproductive factors, including delayed childbearing, lower parity, reduced breastfeeding, earlier menarche, and later menopause [23,25]. Based on the ARIMA model fitting results presented in this study, it is predicted that breast cancer incidence and related deaths will continue to rise in Asia for the next 10 years.

In 2021, the high-income Asia Pacific region recorded the highest ASIR for breast cancer in Asia, while South Asia had the lowest value for this parameter. During the same year, Southeast Asia exhibited the highest ASDR and DALYs rate, whereas East Asia reported the lowest values for these two parameters. While ≥70% of breast malignancies in high-income nations are detected at initial stages (stage 1–2), low/middle-income countries report 20–50% advanced-stage diagnoses, correlating with limited diagnostic capacity across the cancer care continuum [26]. Breast cancer diagnosis and treatment delays correlate with adverse survival outcomes [27,28]. This may partly explain the results observed in the high-income Asia Pacific region, although the highest trends in ASIR, ASDR, and age-standardized mortality rates have been recorded in Southeast Asia.

Delays in the diagnosis and treatment of breast cancer may be attributed to one or more of the following factors: delays in patient screening, delays in seeking care by patients or healthcare providers, delays in accessing healthcare services, or delays in initiating appropriate treatment [29]. Multifactorial determinants of delayed breast cancer diagnosis encompass demographic variables (age, marital status), socioeconomic parameters (insurance coverage), clinical history (benign breast disease), biological characteristics (menopausal status, tumor phenotype), and symptomatic presentation patterns [30,31,32,33,34]. A delay in cancer diagnosis not only reduces the chances of survival of a patient, it may also increase medical costs because more invasive treatments may be required [30].

In China (located in East Asia), the surgical management of breast cancer exhibits significant variability across provinces and regions, and even within the same local area. The rate of breast-conserving surgery remains notably low in general hospitals nationwide. While sentinel lymph node biopsy is gaining traction in urban centers, it has yet to be widely adopted by most patients with early-stage breast cancer, with its prevalence currently below 5% [24]. In India (located in South Asia), there are no organized large-scale breast cancer screening programs. The few existing initiatives primarily target small communities and cover only a minimal fraction of the population, relying on funding from research grants or out-of-pocket payments by individuals. Consequently, nearly all breast cancer cases in India are detected clinically instead of by routine screening. Up to two-thirds of patients present with locally advanced disease, while 6–25% have metastatic cancer at diagnosis. A significant proportion of patients are diagnosed with T2/T3 tumors, and notably, up to one-third exhibit skin and/or chest wall involvement (T4a-c). Inflammatory breast cancer is more prevalent among younger patients, significantly reducing their survival prospects [24,35]. As the most populous country in South Asia, the low detection rate of early breast cancer in India is also related to the low ASIR in South Asia observed in our study results. The 5-year survival rate was 82.2% for breast cancer in Japan and 80.3% for invasive breast cancer in South Korea. The 10-year survival rate was 70.3% [24], and the survival rate was closely related to the detection rate of early breast cancer and medical treatment.

Diagnostic infrastructure deficits in resource-limited settings correlate with compromised clinical accuracy due to specialized human scarcity [36]. Similarly, resource-constrained settings demonstrate systemic barriers to breast cancer management, principally characterized by limited primary healthcare availability and restricted access to clinical specialist consultations [37]. For example, sentinel lymph node biopsy serves as the standard axillary staging modality for early-stage breast cancer in the United States, as well as in Canada and Sweden, supported by multicenter clinical validation [38,39], but it is still a new technology that is being evaluated in Asia. This is also why the breast cancer burden is unevenly distributed between countries with different SDIs. The benefits of a global approach to breast cancer lie in the rapid sharing of preventive measures and appropriate treatments between the more developed and the developing countries [21,23,40].

Our present findings revealed that during the study period from 1990 to 2021, breast cancer-related incidence, deaths, and DALYs among various age groups in Asian women have shown an overall rising trend. In 2021, the incidence and DALY values related to breast cancer in Asia peaked in the 50–54 year age group. Some studies have suggested that the peak occurrence of breast cancer in the West is 55–60 years of age [24]. The observed disparities in breast cancer incidence rates are attributed to multifactorial determinants encompassing geographical disparities, racial/ethnic composition, genetic predisposition, lifestyle patterns, environmental exposures, socioeconomic inequalities, prevalence of established risk factors, mammography screening adherence, stage at diagnosis, and disparities in treatment accessibility [22].

Providing culturally sensitive care is indeed crucial, especially when addressing health disparities and barriers to healthcare access among diverse populations. A study conducted in New York City found that structural barriers (language, health insurance status) and sociocultural barriers (lack of cancer prevention, gender roles, stigma, physician gender, and fatalism) are major hurdles to screening for breast and cervical cancer among Muslim women [41]. Before offering a therapeutic care plan, healthcare professionals must critically study the cultural values of Asian women regarding breast cancer diagnosis and treatment [42].

Our research can help policymakers understand the burden of breast cancer in their region or country and give them some useful recommendations. For example, establish a stratified screening system for breast cancer, manage high-risk groups, standardize the use of hormone drugs, and avoid abuse. In China, breast health electives could be offered in universities, early screening programs with ultrasound combined with genetic testing could be developed for women aged 20–35 with a family history, and a national breast cancer efficacy tracking database could be developed. Although GBD studies provide high-quality estimates of the burden associated with multiple diseases and injuries, there may still be some limitations [7]. A GBD study relies primarily on data obtained from globally significant databases and cancer registries, as well as other epidemiological studies, and the results depend on the out-of-sample predictive validity of the modeling effort [7]. In many parts of the world, especially low-income areas, cancer registries may not be fully developed and therefore not reliable enough, which could affect the robustness and generality of our findings [43,44]. Our study analyzed correlations using GBD data, which aggregates population-level estimates without individual exposure timelines or genetic covariates. We did not conduct a test for causality because of data limitations.

## 5. Conclusions

From 1990 to 2021, the measured values for breast cancer-related ASIR, ASDR, and the age-standardized mortality rate in Asian women have showed an overall upward trend. In 2021, the Asian geographic region with the highest ASIR values was the high-income Asia Pacific region while South Asia had the lowest values for these parameters. Notably, in 2021, the highest values for ASDR and age-standardized mortality rate occurred in Southeast Asia, while East Asia had the lowest values for these parameters. From 1990 to 2021, the breast cancer-related incidence, deaths, and DALYs of various age groups (aged ≥15 to 19 years of age) have shown an overall upward trend. In 2021, the values for breast cancer incidence and DALYs were the highest in the 50–54 year age group, with breast cancer-related deaths peaking in the 55–59 year age group. The leading risk factor attributed to breast cancer deaths in Asia in 1990 and again in 2021 was a “diet high in red meat”, and the Asian country with the highest proportion of the “diet high in red meat” related risk factors for breast cancer deaths in 2021 was Vietnam. The absolute and relative SDI-related inequalities in the breast cancer-related DALYs burden in Asia have increased significantly over time. In Asia, the incidence of breast cancer and the number of deaths due to this disease are expected to continue to rise over the next 10 years.

To offer a breast cancer therapy and management plan tailored to Asian women of different ethnicities, healthcare professionals must critically study the cultural values of Asian women and the extent of their knowledge about breast cancer and its screening. The sociocultural profile of this cohort necessitates culturally attuned healthcare models incorporating linguistically adaptive education modules, strategically designed to encourage Asian women’s autonomous engagement with breast cancer prevention and care.

## Figures and Tables

**Table 1:** Changing trends of incidence attributable to Asian breast cancer from 1990 to 2021: results from Global Burden of Disease Study 2021.

Incidence (95% UI)					
Location	1990		2021		EAPC (95% CI)
	Number	Age-Standardized Rate	Number	Age-Standardized Rate	
Indonesia	12,142.86 (8381.07 to 17,254.81)	1954.5% (1343.25% to 2758.54%)	49,021.32 (32,798.52 to 70,414.27)	3370.56% (2251.37% to 4827.76%)	164.09% (155.15% to 173.04%)
Global	865,880.71 (824,337.79 to 900,793.7)	3999.45% (3800.59% to 4160%)	2,082,737.02 (1,940,351.2 to 2,225,082.58)	4640.28% (4325.55% to 4956.08%)	39.93% (35.01% to 44.85%)
China	84,793.22 (68,417.18 to 103,213.48)	1784.36% (1448.11% to 2165.14%)	385,837.7 (294,095.4 to 489,009.76)	3699.83% (2823% to 4694.6%)	234.34% (226.3% to 242.39%)
Malaysia	1850.72 (1561.32 to 2166.34)	3249.01% (2745.04% to 3783.5%)	9383.3 (7852.83 to 11,149.07)	6124.82% (5133.5% to 7263.09%)	210.22% (200.51% to 219.94%)
Taiwan (Province of China)	2122.46 (1984.44 to 2270.56)	2412.15% (2259.17% to 2570.41%)	10,772.64 (9739.99 to 11,798.99)	5496.19% (4951.25% to 6021.12%)	271.93% (233.63% to 310.37%)
Viet Nam	3157.26 (2412.21 to 4128.57)	1335.19% (1022.74% to 1745.21%)	15,593.5 (11,666.77 to 21,040.09)	2658.95% (1996.9% to 3571.58%)	232.04% (228.05% to 236.03%)
Lao People’s Democratic Republic	211.33 (122.05 to 347.12)	1703% (1010.69% to 2737.17%)	813.84 (569.89 to 1120.28)	2816.51% (1995.77% to 3883.04%)	174.78% (170.52% to 179.05%)
Myanmar	3409.95 (2284.63 to 4918.5)	2345.27% (1620.13% to 3317.2%)	9516.53 (7231.16 to 12,579.91)	3181.27% (2443.94% to 4194.58%)	83.68% (76.05% to 91.32%)
East Asia	88,619.9 (72,204.6 to 107,164.47)	1791.51% (1467.02% to 2159.3%)	401,075.8 (311,440.05 to 503,565.63)	3712.02% (2881.8% to 4670.01%)	234.92% (227.01% to 242.84%)
Cambodia	515.56 (323.82 to 791.76)	1697.71% (1081.01% to 2574.66%)	2644.27 (1887.74 to 3563.4)	3355.16% (2404.93% to 4479.22%)	225.72% (221.1% to 230.34%)
Kazakhstan	3072.04 (2723.19 to 3437.64)	3939.47% (3493.28% to 4412.76%)	3527.27 (2952.58 to 4137.08)	3224.19% (2699.99% to 3779.88%)	3% (−17.25% to 23.3%)
Democratic People’s Republic of Korea	1704.23 (1116.39 to 2499.48)	1608.39% (1058.88% to 2341.26%)	4465.46 (3014.67 to 6197.05)	2504.08% (1687.11% to 3485.25%)	167.04% (156.11% to 177.98%)
Sri Lanka	1086.57 (897.54 to 1309.97)	1727.39% (1424.46% to 2083.6%)	4407.79 (2837.9 to 6075.92)	3038.42% (1955.52% to 4166.43%)	218.33% (202.95% to 233.74%)
Mongolia	48.69 (38.17 to 61.68)	798.76% (616.57% to 1021.95%)	180.63 (140.35 to 224)	1136.86% (874.4% to 1412.04%)	107.47% (95.72% to 119.23%)
Timor-Leste	22.76 (13.88 to 34.34)	1242.4% (796.72% to 1867.62%)	98.16 (66.31 to 134.13)	2159.88% (1467.75% to 2971.73%)	196.89% (174.64% to 219.2%)
Southeast Asia	32,374.19 (27,338.92 to 38,922.22)	2029.95% (1718.51% to 2416.11%)	138,058.13 (115,120.95 to 167,272.8)	3615.01% (3020.23% to 4367.76%)	183.99% (177.12% to 190.87%)
Maldives	6.59 (3.28 to 11.53)	1375.44% (766.99% to 2274.42%)	39.16 (29.59 to 50.24)	2115.51% (1624.73% to 2677.57%)	119.29% (90.8% to 147.85%)
Philippines	5261.12 (4687.01 to 5872.97)	2938.67% (2610.02% to 3300.77%)	21,322.34 (16,755.57 to 26,651.38)	4329.55% (3411.05% to 5381.23%)	117.65% (105.81% to 129.5%)
Armenia	940.98 (878.91 to 1009.78)	5842.21% (5447.45% to 6268.46%)	1162.7 (997.47 to 1338.12)	4883.85% (4211.85% to 5597.81%)	−51.8% (−83.35% to −20.15%)
Kyrgyzstan	537.66 (479.11 to 599.42)	3111.92% (2764.55% to 3484.54%)	796.93 (645.47 to 965.1)	2579.17% (2109.86% to 3122.77%)	−77.58% (−102.18% to −52.91%)
Thailand	4566.96 (3687.53 to 5448.29)	1983.1% (1611.35% to 2361.79%)	24,500.44 (18,387.05 to 31,651.67)	4608.04% (3450.7% to 5993.96%)	284.56% (257.97% to 311.2%)
Central Asia	9815.88 (9236.58 to 10,314.85)	3539.24% (3331% to 3722.64%)	15,355.91 (13,707.25 to 17,195.1)	3096.86% (2774.83% to 3460.79%)	−18.97% (−26.04% to −11.89%)
Republic of Korea	2563.95 (2218.39 to 3032.28)	1267.76% (1100.15% to 1499.11%)	16,111.5 (13,004.46 to 19,081.2)	3870% (3159.29% to 4600.2%)	396.83% (361.24% to 432.53%)
Georgia	2033.58 (1813.75 to 2244.41)	5896.65% (5256.1% to 6493.6%)	1947.24 (1667.9 to 2257.13)	6386.47% (5482.06% to 7435.85%)	35.73% (7.86% to 63.68%)
Azerbaijan	904.59 (751.6 to 1036.51)	3006.95% (2481.54% to 3447.05%)	2055.39 (1497.72 to 2669.23)	3237.07% (2382.18% to 4199.07%)	36.99% (16.64% to 57.38%)
Tajikistan	368.24 (299.36 to 443.85)	2344.69% (1898.01% to 2831.83%)	763.65 (465.14 to 1135.98)	1990.3% (1257.12% to 2924.83%)	−70.91% (−84.75% to −57.05%)
Japan	28,068.16 (26,425.25 to 29,536.27)	3237.54% (3052.61% to 3404.01%)	78,020.96 (67,786.77 to 84,828.31)	6228.36% (5684.49% to 6649.06%)	216.24% (192.49% to 240.04%)
Nepal	546.37 (400.45 to 719.37)	984.98% (713.71% to 1291.67%)	2082.31 (1493.47 to 2866.08)	1506.6% (1081.09% to 2086.17%)	143.49% (120.52% to 166.52%)
Singapore	579.83 (538.13 to 626.86)	4067.62% (3790.59% to 4386.49%)	2445.81 (2234.29 to 2659.2)	5763.29% (5286.2% to 6279.54%)	156.5% (131.87% to 181.18%)
High-income Asia Pacific	31,234.99 (29,577.64 to 32,777.32)	2882.9% (2731.28% to 3019.08%)	96,689.37 (84,849.88 to 104,793.61)	5562.38% (5048.57% to 5945.23%)	222.19% (198.57% to 245.85%)
Turkmenistan	275.68 (240.26 to 314.23)	2335.03% (2034.97% to 2656.77%)	688.65 (504.42 to 933.63)	2774.17% (2045.18% to 3745.05%)	107.77% (58.9% to 156.88%)
Bangladesh	2415.17 (1792.19 to 3347.46)	843.34% (628.8% to 1154.42%)	12,911.45 (9662.07 to 16,599.87)	1589.06% (1191.54% to 2042.41%)	185.25% (169.37% to 201.16%)
Seychelles	7.57 (6.49 to 8.82)	2577.61% (2206.99% to 3000.69%)	28.92 (24.48 to 34.01)	4723.2% (3988.54% to 5552.07%)	196.57% (162.46% to 230.78%)
Brunei Darussalam	23.05 (16.87 to 30.39)	3346.92% (2495.21% to 4428.3%)	111.09 (85.86 to 137.86)	4927.3% (3825.9% to 6100.89%)	180.83% (162.79% to 198.91%)
South Asia	43,743.57 (38,840.26 to 49,290.21)	1310.65% (1156.87% to 1477.74%)	205,357.1 (179,412.09 to 235,439.18)	2462.48% (2151.53% to 2832.02%)	197.11% (181.25% to 212.99%)
Uzbekistan	1634.42 (1466.66 to 1811.41)	2430.31% (2182.02% to 2707.2%)	4233.44 (3450.76 to 5076.11)	2490.29% (2040.65% to 2986.35%)	22.54% (−0.17% to 45.3%)
India	33,068.29 (28,439.8 to 38,559.92)	1210.36% (1033.85% to 1421.85%)	156,160.16 (131,883.24 to 185,936.55)	2352.43% (1982.04% to 2804.43%)	217.69% (196.75% to 238.67%)
Pakistan	7699.25 (6040.58 to 9582.47)	2646.61% (2066.99% to 3312.7%)	34,152.67 (24,163.77 to 45,770.5)	4590.51% (3300.83% to 6076.76%)	143.55% (126.01% to 161.13%)
Mauritius	88.11 (81.52 to 94.82)	2023.11% (1872.59% to 2173.82%)	495.99 (448.3 to 533.48)	5357.52% (4811.98% to 5782.07%)	241.69% (202.13% to 281.4%)
Bhutan	14.49 (9.92 to 19.4)	974.11% (679.96% to 1301.96%)	50.5 (35 to 70.74)	1544.16% (1076.73% to 2166.45%)	144.05% (131.93% to 156.18%)
Asia	221,631.76 (199,674.3 to 247,609.83)	1905.72% (1719.34% to 2118.09%)	934,358.23 (837,386.96 to 1,050,748.44)	3522.78% (3161.77% to 3958.93%)	199.09% (194.95% to 203.23%)

**Table 2:** Changing trends of percentage change attributable to Asian breast cancer from 1990 to 2021: results from Global Burden of Disease Study 2021.

Location	Percentage Change in Age-Standardized Rate, 1990–2021 (per 100,000)		
	Deaths	Incidence	DALYs
Azerbaijan	−16.98% (−38.03% to 6.47%)	7.65% (−21.54% to 39%)	−19.95% (−41.48% to 2.61%)
Georgia	4.97% (−13.56% to 22.9%)	8.31% (−10.25% to 28.41%)	−7.64% (−23.93% to 8.84%)
Kazakhstan	−38.92% (−50.13% to −25.06%)	−18.16% (−32.86% to 0.48%)	−41.33% (−52.01% to −28.57%)
Kyrgyzstan	−36.16% (−47.46% to −20.27%)	−17.12% (−33.81% to 6.06%)	−37.61% (−50.06% to −20.67%)
South Asia	40.82% (19.34% to 66.4%)	87.88% (60.08% to 121.38%)	38.67% (18.04% to 63.43%)
East Asia	−6.87% (−30.95% to 27.32%)	107.2% (50.33% to 189.58%)	−5.45% (−30.64% to 31.58%)
Mauritius	96.68% (72.91% to 116.7%)	164.82% (132.62% to 194.34%)	99.41% (73.93% to 121.45%)
Bangladesh	22.63% (−14.57% to 84.54%)	88.42% (29.39% to 188.67%)	23.63% (−15.39% to 89.02%)
China	−8.24% (−33.32% to 27.62%)	107.35% (49.12% to 193.42%)	−6.67% (−32.59% to 32.16%)
Central Asia	−25.82% (−34.07% to −16.89%)	−12.5% (−22.56% to −0.96%)	−29.64% (−38.08% to −20.4%)
Democratic People’s Republic of Korea	15.13% (−24.21% to 70.27%)	55.69% (2.73% to 134.19%)	17.35% (−23.56% to 78.05%)
Bhutan	15.67% (−22.78% to 72.53%)	58.52% (2.86% to 145.13%)	10.97% (−28.86% to 72.23%)
Asia	16.9% (3.83% to 31.97%)	84.85% (59.08% to 115.61%)	17.31% (2.72% to 32.8%)
Global	−12.36% (−17.11% to −7.18%)	16.02% (9.18% to 23.67%)	−9.58% (−15.36% to −3.26%)
Japan	40.04% (30.31% to 46.3%)	92.38% (76.7% to 108.04%)	29.22% (23% to 34.37%)
India	44.23% (21.75% to 73.27%)	94.36% (63.9% to 132.87%)	38.89% (16.98% to 66.53%)
Viet Nam	29.63% (−7.87% to 81.57%)	99.14% (35.16% to 183.93%)	26.5% (−12.01% to 82.73%)
Sri Lanka	10.11% (−31.69% to 60.88%)	75.9% (7% to 160.35%)	9.95% (−32.6% to 58.79%)
Armenia	−29.26% (−39.4% to −17.01%)	−16.4% (−28.83% to −2.3%)	−40.87% (−49.38% to −31.31%)
Nepal	15.32% (−20.28% to 71.12%)	52.96% (4.18% to 128.62%)	12.84% (−23.61% to 74.37%)
Thailand	45.82% (4.55% to 107.5%)	132.37% (65.97% to 228.45%)	44.54% (2.58% to 104.64%)
Seychelles	40.89% (13.87% to 73.48%)	83.24% (45.91% to 129.82%)	34.72% (8.14% to 68.24%)
Pakistan	48.61% (2.1% to 105.89%)	73.45% (18.02% to 145.3%)	47.33% (1.18% to 108.27%)
Timor-Leste	41% (−6.07% to 122.14%)	73.85% (15.08% to 185.27%)	42.81% (−7.6% to 136.53%)
Taiwan (Province of China)	40.18% (26.71% to 53.63%)	127.85% (101.3% to 154.7%)	34.62% (21.33% to 47.39%)
Mongolia	5.34% (−25.76% to 47.07%)	42.33% (1.19% to 99.7%)	1.08% (−28.88% to 38.97%)
Southeast Asia	32.2% (12.19% to 56.32%)	78.08% (51.59% to 109.68%)	29.72% (9.2% to 54.69%)
Cambodia	50.59% (−7.58% to 151.12%)	97.63% (21.55% to 237.98%)	48.33% (−11.16% to 154.97%)
Indonesia	38.31% (1.64% to 87.58%)	72.45% (26.4% to 139.38%)	33.68% (−1.46% to 84.61%)
Lao People’s Democratic Republic	30.85% (−22.13% to 124.46%)	65.39% (−3.57% to 186.68%)	28.76% (−25.49% to 127.66%)
Malaysia	32.68% (7.83% to 65.83%)	88.51% (50.51% to 133.88%)	29.44% (4.3% to 59.31%)
Maldives	−10.69% (−50.56% to 67.08%)	53.81% (−19.54% to 212.5%)	−13.79% (−56.48% to 85.12%)
Republic of Korea	18.78% (−11.46% to 45.29%)	205.26% (129.31% to 274.16%)	20.42% (−8.89% to 48.39%)
Myanmar	5.82% (−31.37% to 62.99%)	35.65% (−12.61% to 114.99%)	−0.37% (−37.01% to 60.1%)
Philippines	19.26% (−8.77% to 54.57%)	47.33% (13.1% to 88%)	30.06% (−1.07% to 67.69%)
High-income Asia Pacific	30.68% (20.3% to 37.75%)	92.94% (77.77% to 108.41%)	21.79% (13.52% to 28.45%)
Tajikistan	−24.66% (−51.07% to 9.92%)	−15.11% (−46.91% to 27.34%)	−25.82% (−54.4% to 13.01%)
Turkmenistan	−8.32% (−31.55% to 23.2%)	18.81% (−13.55% to 61.89%)	−4.73% (−30.09% to 30.09%)
Uzbekistan	−14.89% (−30.08% to 4.69%)	2.47% (−16.51% to 25.56%)	−14.06% (−29.06% to 4.95%)
Singapore	−25.31% (−31.43% to −18%)	41.69% (26.59% to 59.24%)	−27.74% (−33.66% to −20.73%)
Brunei Darussalam	7.29% (−24.14% to 50.4%)	47.22% (3.22% to 108.74%)	5.17% (−26.2% to 49.62%)

**Table 3:** Changing trends of deaths attributable to Asian breast cancer from 1990 to 2021: results from Global Burden of Disease Study 2021.

Deaths (95% UI)					
Location	1980		2021		EAPC (95% CI)
	Number	Age-Standardized Rate	Number	Age-Standardized Rate	
Timor-Leste	11.6 (7.16 to 17.51)	882.24% (546.88% to 1306.85%)	59.52 (40.68 to 81.51)	1362.57% (935.87% to 1861.06%)	106.54% (94.97% to 118.12%)
Global	277,498.86 (261,536.89 to 293,328.4)	1633.69% (1539.19% to 1722.48%)	660,925.3 (609,171.34 to 707,181.86)	1454.99% (1345.14% to 1555.89%)	−39.95% (−45.54% to −34.37%)
Kazakhstan	1310.13 (1154.25 to 1487.8)	1917.48% (1687.31% to 2179.43%)	1374.96 (1140.5 to 1602.48)	1284.55% (1067.48% to 1495.66%)	−93.59% (−117.01% to −70.12%)
Singapore	126.86 (118.25 to 135.38)	1519.65% (1417.65% to 1623.86%)	481.65 (434.69 to 522.09)	1102.01% (1000.65% to 1192.38%)	−72.85% (−84.28% to −61.4%)
Viet Nam	1508.45 (1099.63 to 2046.21)	775.12% (566.01% to 1048.97%)	6220.77 (4668.04 to 8248.77)	1084.85% (817.5% to 1425.45%)	87.6% (85.46% to 89.75%)
Kyrgyzstan	261.56 (224.23 to 300.11)	1686.87% (1447.57% to 1933.15%)	328.55 (268.64 to 392.12)	1117.45% (918.91% to 1338.24%)	−127.18% (−142.4% to −111.95%)
Republic of Korea	845.64 (637.29 to 1137.23)	601.06% (454.78% to 799.2%)	2858.76 (2275.66 to 3450.22)	625.04% (502.16% to 751.06%)	41.86% (33.54% to 50.19%)
China	33,812.29 (25,987.93 to 43,763.69)	993.43% (773.01% to 1270.76%)	88,106.72 (68,162.64 to 110,341.23)	823.68% (636.95% to 1033.47%)	−50.65% (−59.52% to −41.77%)
East Asia	34,931.98 (26,977.89 to 45,023.5)	986.21% (773.75% to 1252.73%)	92,961.69 (73,229.73 to 115,384.35)	837.98% (659.5% to 1039.86%)	−43.11% (−51.55% to −34.67%)
Mauritius	53.53 (49.78 to 57.39)	1660.67% (1548.25% to 1777.18%)	220.79 (199.62 to 235.25)	2289.86% (2070.21% to 2436.03%)	184.07% (151.8% to 216.43%)
Tajikistan	175.17 (130.12 to 227.47)	1341.88% (1001.76% to 1743.4%)	374.65 (235.46 to 551.52)	1060.16% (703.12% to 1505.82%)	−73.13% (−84.46% to −61.78%)
Seychelles	3.35 (2.75 to 3.93)	1288.5% (1058.7% to 1508.23%)	13.28 (11.2 to 15.48)	2177.6% (1841.72% to 2539.19%)	168.67% (144.21% to 193.19%)
Mongolia	25.87 (18.44 to 35.14)	491.77% (350.38% to 669.26%)	84.2 (64.97 to 103.85)	575.73% (439.33% to 710.15%)	46.29% (27.77% to 64.85%)
Turkmenistan	127.71 (110.41 to 147.16)	1321.04% (1142.89% to 1521.67%)	300.69 (223.56 to 401.87)	1245.82% (931.73% to 1655.69%)	−26.29% (−55.52% to 3.03%)
Taiwan (Province of China)	393.2 (368.41 to 419.84)	664.39% (625.47% to 707.09%)	2817.17 (2538.69 to 3072.85)	1328.03% (1205.55% to 1441.87%)	180.9% (158.51% to 203.34%)
Uzbekistan	695.9 (624.59 to 770.24)	1241.82% (1113.88% to 1376.48%)	1841.46 (1532.27 to 2209.72)	1142.23% (953.72% to 1368.86%)	−32.73% (−46.6% to −18.83%)
Southeast Asia	13,021.86 (10,376.24 to 16,349.06)	1149.53% (931.08% to 1423.94%)	65,437.37 (54,265.99 to 79,833.65)	1772.42% (1476.27% to 2151.5%)	108.44% (100.66% to 116.22%)
Democratic People’s Republic of Korea	726.48 (478.75 to 1084.08)	914.43% (607.66% to 1363.31%)	2037.8 (1414.63 to 2733.12)	1104.25% (768.62% to 1476.72%)	56.91% (52.67% to 61.16%)
Cambodia	294.12 (175.29 to 465.75)	1273.07% (783.37% to 1966.24%)	1493.19 (1073.23 to 1986.48)	1983.52% (1432.73% to 2611.9%)	121.47% (116.61% to 126.32%)
South Asia	20,392.37 (17,113.98 to 24,001.36)	852.23% (715.64% to 1000.55%)	105,497.03 (92,006.39 to 121,287.14)	1324.41% (1153.67% to 1528.48%)	109.39% (101.82% to 116.96%)
Indonesia	4503.83 (2907.43 to 6816.06)	1039.77% (677.63% to 1578.71%)	25,686.71 (16,881.86 to 37,378)	1891.76% (1238.08% to 2728.38%)	144.53% (129.1% to 159.99%)
Asia	81,979.86 (70,853.04 to 96,019.81)	971.72% (846.65% to 1122.76%)	306,494.59 (276,354.3 to 340,676.9)	1159.18% (1046.6% to 1288.24%)	44.39% (40.05% to 48.73%)
Central Asia	4010.8 (3792.97 to 4260.45)	1682.17% (1591.1% to 1786.5%)	6608.11 (5910.1 to 7374.62)	1383.16% (1241.38% to 1537.35%)	−53.89% (−64.45% to −43.32%)
Armenia	291.44 (264.05 to 319.23)	2163.79% (1961.2% to 2365.34%)	499.58 (437.07 to 580.28)	2012.56% (1766.33% to 2327.09%)	−0.06% (−37.04% to 37.05%)
Brunei Darussalam	7.97 (5.43 to 11.05)	1851.72% (1302.29% to 2496.96%)	38.27 (29.68 to 47.06)	1830.47% (1432.95% to 2244.45%)	22.97% (1.99% to 43.99%)
Malaysia	723.51 (577.73 to 919.54)	1802.31% (1431.05% to 2307.85%)	3873.77 (3292.24 to 4520.72)	2653.69% (2254.98% to 3094.57%)	88.62% (81.43% to 95.82%)
India	15,571.59 (12,570.02 to 19,120.26)	804.69% (649.6% to 982.93%)	78,878.68 (66,512.08 to 94,203.94)	1236.54% (1039.66% to 1476.56%)	108.25% (97.96% to 118.56%)
Bangladesh	1285.7 (824.54 to 1996.57)	566.75% (367.9% to 871.99%)	5572.51 (4201.33 to 7204.89)	706.56% (533.95% to 908.79%)	48.05% (38.96% to 57.15%)
Japan	4830.83 (4654.11 to 4967.08)	686.59% (660.23% to 707.26%)	16,709.57 (13,790.59 to 18,359.72)	1026.95% (914.87% to 1087.23%)	128.85% (118.08% to 139.64%)
Nepal	306.91 (209.94 to 418.18)	752.45% (526.26% to 1011.57%)	1125.32 (797.74 to 1561.3)	865.58% (619.19% to 1203.79%)	20.49% (4.53% to 36.47%)
Bhutan	6.85 (4.58 to 9.75)	643.6% (431.94% to 904.27%)	26.35 (18.5 to 36.04)	842.81% (598.32% to 1143.9%)	57.39% (49.71% to 65.09%)
Maldives	3.13 (1.34 to 5.87)	980.09% (503.33% to 1662.98%)	14.18 (10.82 to 17.95)	862.16% (677.13% to 1081.41%)	−20.13% (−41.22% to 1.01%)
High-income Asia Pacific	5811.3 (5521.55 to 6136.36)	684.59% (649.19% to 722.41%)	20,088.25 (16,719.24 to 22,018.71)	926.13% (825.76% to 986.22%)	103.48% (93.77% to 113.2%)
Azerbaijan	409.6 (346.91 to 482.85)	1652.79% (1409.42% to 1942.32%)	863.71 (646.59 to 1112.87)	1412.37% (1072.58% to 1796.6%)	−41.67% (−50.22% to −33.1%)
Myanmar	1628.61 (1064.77 to 2402.72)	1468.45% (1002.06% to 2109.49%)	5256.86 (4038.3 to 7040.74)	1805.77% (1397.09% to 2379.84%)	39% (26.98% to 51.04%)
Georgia	713.43 (625.89 to 802.34)	2224.37% (1958.14% to 2494.54%)	940.31 (810.32 to 1079.81)	2811.73% (2415.15% to 3257.84%)	49.91% (31.25% to 68.61%)
Lao People’s Democratic Republic	127.1 (73.08 to 214.77)	1327.39% (793.22% to 2153.69%)	481.74 (339.04 to 670.23)	1789.92% (1283.61% to 2465.47%)	90.25% (85.91% to 94.6%)
Pakistan	3221.32 (2279.73 to 4364.56)	1590.78% (1096.2% to 2136.15%)	19,894.18 (14,316.08 to 26,527.58)	2976.42% (2158.15% to 3907.74%)	162.14% (139.58% to 184.75%)
Thailand	1546.11 (1210.79 to 1889.83)	1051.75% (821.28% to 1293.59%)	9036.1 (6777.12 to 11,439.54)	1597.64% (1200.69% to 2024.31%)	130.47% (116.45% to 144.51%)
Philippines	2153.98 (1916.17 to 2401.61)	1860.34% (1659.17% to 2062.09%)	11,239.95 (8829.03 to 14,035.7)	2398.4% (1896.87% to 2977.04%)	53.49% (46.41% to 60.57%)
Sri Lanka	445 (368.86 to 538.51)	1098.19% (902.01% to 1324.72%)	1749.24 (1142.55 to 2358.07)	1186.66% (778.88% to 1599.55%)	37.88% (28.75% to 47.02%)

**Table 4:** Changing trends of DALYs attributable to Asian breast cancer from 1990 to 2021: results from Global Burden of Disease Study 2021.

	DALYs (95% UI)				
Location	1990		2021		EAPC (95% CI)
	Number	Age-Standardized Rate	Number	Age-Standardized Rate	
Myanmar	92,162.6 (61,697.47 to 133,542.73)	61,375.16% (41,601.01% to 87,391.65%)	184,930.73 (137,434.95 to 249,880.71)	61,148.39% (45,696.91% to 82,468.89%)	−22.4% (−34.08% to −10.71%)
Global	11,036,401.95 (10,434,964.35 to 11,671,316.52)	50,380.75% (47,590.78% to 53,222.54%)	20,254,801.61 (18,963,375.54 to 21,574,428.57)	45,555.95% (42,663.99% to 48,529.8%)	−45.9% (−51.58% to −40.21%)
Philippines	113,044.67 (100,563.65 to 124,430.64)	59,877.65% (53,150.86% to 66,280.57%)	389,501.17 (302,649.81 to 491,218.37)	77,876.81% (60,769.37% to 97,827.51%)	87.14% (79.78% to 94.5%)
East Asia	1,530,662.9 (1,238,542.99 to 1,864,753.77)	30,274.63% (24,616.25% to 36,791.43%)	3,077,415.02 (2,410,862.12 to 3,867,298.92)	28,625.16% (22,385.77% to 36,040.98%)	−45.78% (−56.76% to −34.79%)
High-income Asia Pacific	282,740.27 (270,991.17 to 295,992.78)	26,283.3% (25,175.72% to 27,512.98%)	539,252.33 (480,456.33 to 585,167.58)	32,010.08% (29,448.68% to 34,412.12%)	61.62% (45.62% to 77.64%)
Turkmenistan	5497.76 (4790.87 to 6251.72)	45,792.28% (39,994.95% to 52,040.68%)	10,917.43 (8083.32 to 14,713.45)	43,628.34% (32,354.62% to 58,640.53%)	34.31% (−10.94% to 79.77%)
Sri Lanka	21,547.02 (17,890.12 to 25,782.79)	33,188.64% (27,568.88% to 39,700.47%)	52,913.57 (34,230.66 to 72,188.76)	36,490.28% (23,565.89% to 49,666.51%)	55.77% (41.5% to 70.06%)
Tajikistan	7578.03 (6063.91 to 9167.29)	47,603.98% (38,080.31% to 57,592.43%)	13,958.3 (8412.46 to 21,122.51)	35,313.18% (22,152.03% to 52,215.84%)	−110.25% (−120.81% to −99.67%)
South Asia	1,106,160.68 (979,672.69 to 1,249,654.8)	31,491.45% (27,855.72% to 35,557.84%)	3,710,897.48 (3,235,644.4 to 4,263,863.2)	43,668.81% (38,058.94% to 50,146.15%)	96.45% (83.86% to 109.05%)
Uzbekistan	30,719.99 (27,733.86 to 33,696.44)	45,250.6% (40,693.23% to 49,791.23%)	66,972.57 (55,330.84 to 80,321.18)	38,886.25% (32,277.18% to 46,680.48%)	−44.5% (−63.93% to −25.02%)
Thailand	86,975.18 (70,251.2 to 104,180.92)	37,051.05% (30,083.64% to 44,066.54%)	285,453.73 (213,281.73 to 364,389.13)	53,554.61% (40,380.53% to 68,707.48%)	121.25% (98.41% to 144.13%)
China	1,466,485.56 (1,177,503.3 to 1,798,026.6)	30,167.57% (24,316.89% to 36,876.26%)	2,921,096.2 (2,254,510.09 to 3,716,738.57)	28,154.18% (21,687.06% to 35,810.57%)	−52.15% (−63.9% to −40.39%)
Brunei Darussalam	423.3 (310.19 to 557.77)	58,767.67% (43,256.2% to 78,588%)	1427.46 (1101.61 to 1751.85)	61,807.68% (47,896.34% to 75,667.22%)	72.46% (45.75% to 99.25%)
Armenia	15,422.1 (14,370.6 to 16,405.17)	94,695.87% (88,204.38% to 100,770.28%)	13,247.59 (11,616.82 to 15,194.94)	55,996.02% (491,77.98% to 64,091.22%)	−174.01% (−206.55% to −141.36%)
Japan	235,044.59 (224,566.71 to 244,515.97)	27,315.28% (26,171.86% to 28,413.6%)	425,081.29 (376,703.9 to 462,267.44)	35,297.26% (32,674.49% to 37,557.83%)	78.73% (60.78% to 96.71%)
Seychelles	147.24 (125.33 to 171.61)	50,687.22% (42,879.81% to 59,067.74%)	420.54 (354.63 to 493.04)	68,284.69% (57,818.83% to 80,285.99%)	98.95% (71.07% to 126.91%)
Asia	3,993,737.13 (3,554,041.8 to 4,499,412.85)	33,306.96% (29,761.04% to 37,431.12%)	10,335,176.26 (9,280,423.16 to 11,485,399.63)	39,072.56% (35,105.65% to 43,365.19%)	38.88% (32.52% to 45.25%)
Azerbaijan	17,484.69 (14,510.46 to 20,075.48)	57,369.52% (47,351.01% to 65,714.91%)	29,491.83 (21,841.21 to 38,003.77)	45,921.5% (34,147.97% to 58,731.71%)	−72.53% (−82.96% to −62.08%)
Bangladesh	67,921.65 (50,342.84 to 93,448.25)	22,805.47% (16,930.13% to 31,311.93%)	230,781.71 (172,029.7 to 298,633.45)	28,194.5% (21,126.94% to 36,353.32%)	44.32% (30% to 58.65%)
Mauritius	1605.29 (1494.32 to 1738.78)	36,396.19% (33,865.87% to 39,390.37%)	6702.16 (6037.52 to 7186.27)	72,577.38% (65,051.02% to 78,243.15%)	160.98% (128.32% to 193.74%)
Central Asia	172,576.09 (163,121.53 to 181,554.43)	61,866.69% (58,413.68% to 65,115.68%)	218,123.81 (193,868.77 to 245,238.63)	43,530.89% (38,795.24% to 48,761.36%)	−99.5% (−108.5% to −90.5%)
Bhutan	380.63 (256.51 to 508.63)	24,143.2% (16,640.68% to 32,179%)	891.8 (608.66 to 1247.2)	26,790.65% (18,347.94% to 37,302.32%)	21.17% (10.91% to 31.45%)
Timor-Leste	593.34 (367.43 to 915.57)	30,056.41% (18,931.77% to 46,092.84%)	1975.8 (1342.7 to 2709.39)	42,922.19% (29,032.86% to 59,028.54%)	123.86% (100.26% to 147.52%)
Republic of Korea	40,123.37 (35,318.03 to 47,556.34)	19,512.22% (17,206.33% to 23,161.32%)	97,660.51 (79,028.92 to 116,208.88)	23,497.57% (19,219.56% to 27,801.39%)	68.12% (60.67% to 75.56%)
Viet Nam	66,174.82 (50,516.57 to 86,822.58)	27,758.82% (21,117.7% to 36,347.48%)	207,014.67 (154,595.56 to 280,225.74)	35,114.09% (26,322.98% to 47,212.25%)	81.92% (77.76% to 86.08%)
Georgia	30,993.45 (27,722.32 to 34,432.93)	90,450.74% (80,812.06% to 100,310.59%)	25,077.78 (21,464.47 to 29,112.52)	83,536.16% (71,255.65% to 97,215.52%)	−2.5% (−26.54% to 21.59%)
Taiwan (Province of China)	29,294.47 (27,473.09 to 31,024.07)	33,174.87% (31,191.87% to 35,123.86%)	88,354.87 (80,637.94 to 95,743.26)	44,660.03% (40,776.4% to 48,263.61%)	97.16% (77.43% to 116.94%)
India	829,960.28 (711,198.14 to 969,448.51)	28,929.54% (24,651.3% to 33,879.96%)	2,706,295.31 (2,287,548.07 to 3,225,998.66)	40,181.03% (33,932.69% to 47,998.33%)	106.93% (90.07% to 123.82%)
Kazakhstan	53,688.64 (47,825.39 to 59,859)	68,583.14% (61,097.51% to 76,521.36%)	44,122.1 (36,918.78 to 51,609.41)	40,239.46% (33,742.25% to 47,018.19%)	−131.89% (−164.06% to −99.62%)
Cambodia	13,582.6 (8429.27 to 20,733.35)	42,725.8% (26,758.85% to 65,226.98%)	50,872.11 (36,267.04 to 68,945.61)	63,374.22% (45,315.89% to 85,527.81%)	125.7% (121.31% to 130.09%)
Democratic People’s Republic of Korea	34,882.88 (22,726.69 to 51,469.25)	32,478.36% (21,353.19% to 47,823.8%)	67,963.96 (45,695.19 to 92,799.34)	38,113.34% (25,671.92% to 52,212.43%)	65.99% (60.11% to 71.87%)
Indonesia	292,123.5 (201,999.71 to 413,873.59)	45,203.03% (31,165.87% to 63,995.75%)	899,965.19 (599,398.97 to 1,302,308.57)	60,425.17% (40,310.66% to 87,214.57%)	84.73% (73.58% to 95.89%)
Lao People’s Democratic Republic	5790.43 (3274.38 to 9482.83)	44,746.84% (25,802.85% to 72,749.7%)	17,221.49 (12,068.8 to 23,834.25)	57,614.82% (40,378.53% to 79,828.97%)	89.16% (82.47% to 95.85%)
Malaysia	38,144.31 (32,071.86 to 45,025.37)	65,398.77% (54,969.04% to 76,849.95%)	130,602.74 (109,921.11 to 154,884.53)	84,650.66% (71,319.05% to 100,147.05%)	81.2% (67.86% to 94.55%)
Mongolia	1138.98 (883.53 to 1423.75)	18,323.83% (14,249.06% to 23,049.06%)	3011.94 (2361.41 to 3696.12)	18,522.31% (14,419.27% to 22,716.59%)	−21.2% (−38.98% to −3.39%)
Nepal	14,465.44 (10,699.94 to 19,120.04)	24,592.66% (18,206.96% to 32,178.23%)	39,306.73 (27,963.31 to 54,509.02)	27,749.25% (19,869.35% to 38,369.73%)	43.8% (20.35% to 67.3%)
Pakistan	193,432.67 (152,516.13 to 240,097.03)	62,943.62% (49,412.35% to 78,277.91%)	733,621.92 (517,654.81 to 989,920.94)	92,737.36% (66,590.36% to 123,896.93%)	87.05% (64.92% to 109.22%)
Kyrgyzstan	10,052.45 (8907.46 to 11,320.52)	58,135.43% (51,254.32% to 65,776.98%)	11,324.27 (9173.44 to 13,806.16)	36,273.41% (29,495.1% to 43,988.34%)	−176.54% (−192.33% to −160.72%)
Singapore	7149.01 (6690.05 to 7637.94)	49,244.37% (46,112.32% to 52,522.82%)	15,083.07 (13,862.08 to 16,478.69)	35,582.85% (32,727.24% to 38,836.61%)	−75.65% (−92.13% to −59.15%)
Southeast Asia	733,104.19 (608,727.57 to 890,815.54)	44,554.69% (37,050.47% to 54,044.37%)	2,231,170.6 (1,841,701.79 to 2,734,839.98)	57,797.87% (47,690.73% to 70,699.18%)	79% (71.57% to 86.43%)
Maldives	152.7 (74.53 to 270.16)	30,047.64% (15,939.72% to 50,896.76%)	484.69 (359.9 to 632.84)	25,902.61% (19,517.61% to 33,270.75%)	−84.57% (−106.92% to −62.17%)

## Data Availability

The data underlying this article are available in the Global Health Data Exchange at https://vizhub.healthdata.org/gbd-results/ (accessed on 6 January 2025).

## Supplementary Materials

The following supporting information can be downloaded at: https://www.mdpi.com/article/10.3390/curroncol32050267/s1. Supplementary Table S1. Age groups and ASIR of breast cancer in Asia between 1990 and 2021. Supplementary Table S2. Age groups and ASDR of breast cancer in Asia between 1990 and 2021. Supplementary Table S3. Age groups and age-standardized death rate of breast cancer in Asia between 1990 and 2021. Supplementary Table S4. Absolute and relative cross-country inequality in ASDR of Asian breast cancer, 1990–2021. Supplementary Table S5. Frontier analysis on basis of sociodemographic index and ASDR (per 100,000) of breast cancer in Asia in 2021.

## List of Abbreviations

GBD, Global Burden of Disease; DALY, disability-adjusted life-year; SDI, sociodemographic index; ASR, Age-standardized rate; ASIR, age-standardized incidence rates; ASDR, age-standardized DALY rates; EAPC, estimated annual percentage change; UI, uncertainty interval; CI, concentration index; SII, slope index of inequality; ARIMA model, Autoregressive Integrated Moving Average model; APC model, Age–period–cohort analysis.
