# Supplementary material for: Burdens of Breast Cancer and Projections for 2030 Among Women in Asia: Findings from the 2021 Global Burden of Disease Study
Source: Curr Oncol. 2025 May 1;32(5):267. doi: 10.3390/curroncol32050267 (PMC12109881; doi:10.3390/curroncol32050267)
Supplement: Supplementary file 1 [file curroncol-32-00267-s001.zip › curroncol-3520432-supplementary.pdf]

Supplementary Table S1. Age groups and ASIR of breast cancer in Asia between 1990–2021.

Measure	Location	Age	Year	Incidence
Incidence	Asia	95 plus	1990	116.07 (89.51to136.37)
Incidence	Asia	95 plus	1991	119.88 (93.19to140.77)
Incidence	Asia	95 plus	1992	132 (101.95to154.43)
Incidence	Asia	95 plus	1993	146.62 (112.32to171.34)
Incidence	Asia	95 plus	1994	163.64 (123.97to193.78)
Incidence	Asia	95 plus	1995	184.74 (138.99to220.3)
Incidence	Asia	95 plus	1996	199.05 (148.75to236.64)
Incidence	Asia	95 plus	1997	213.77 (158.11to253.33)
Incidence	Asia	95 plus	1998	238.49 (174.69to281.31)
Incidence	Asia	95 plus	1999	259.6 (190.09to307.71)

Incidence	Asia	95 plus	2000	280.91 (203.33to333.79)
Incidence	Asia	95 plus	2001	312.2 (225.05to368.1)
Incidence	Asia	95 plus	2002	346.64 (247.13to408.9)
Incidence	Asia	95 plus	2003	380.74 (272.65to448.25)
Incidence	Asia	95 plus	2004	424.24 (300.44to497.92)
Incidence	Asia	95 plus	2005	462.14 (331.86to543.66)
Incidence	Asia	95 plus	2006	518.73 (370.59to611.64)
Incidence	Asia	95 plus	2007	583.92 (420.52to686.19)
Incidence	Asia	95 plus	2008	631.89 (449.51to744.09)
Incidence	Asia	95 plus	2009	682.63 (482.85to808.1)
Incidence	Asia	95 plus	2010	761.6 (534.79to899.69)
Incidence	Asia	95 plus	2011	860.86 (605.44to1015.13)

Incidence	Asia	95 plus	2012	938.58 (659.16to1110.29)
Incidence	Asia	95 plus	2013	1030.97 (721.58to1211.86)
Incidence	Asia	95 plus	2014	1086.7 (767.36to1275.45)
Incidence	Asia	95 plus	2015	1179.35 (824.05to1396.28)
Incidence	Asia	95 plus	2016	1328.25 (913.34to1566.1)
Incidence	Asia	95 plus	2017	1463.66 (1020.26to1730.87)
Incidence	Asia	95 plus	2018	1618.08 (1122.16to1914.23)
Incidence	Asia	95 plus	2019	1744.61 (1210.23to2070.21)
Incidence	Asia	95 plus	2020	1797.85 (1224.43to2143.49)
Incidence	Asia	95 plus	2021	1955.7 (1334.74to2323.29)
Incidence	Asia	<5	1990	0 (0to0)
Incidence	Asia	<5	1991	0 (0to0)

Incidence	Asia	<5	1992	0<br>(0to0)
Incidence	Asia	<5	1993	0<br>(0to0)
Incidence	Asia	<5	1994	0<br>(0to0)
Incidence	Asia	<5	1995	0<br>(0to0)
Incidence	Asia	<5	1996	0<br>(0to0)
Incidence	Asia	<5	1997	0<br>(0to0)
Incidence	Asia	<5	1998	0<br>(0to0)
Incidence	Asia	<5	1999	0<br>(0to0)
Incidence	Asia	<5	2000	0<br>(0to0)
Incidence	Asia	<5	2001	0<br>(0to0)
Incidence	Asia	<5	2002	0<br>(0to0)
Incidence	Asia	<5	2003	0<br>(0to0)

Incidence	Asia	<5	2004	0<br>(0to0)
Incidence	Asia	<5	2005	0<br>(0to0)
Incidence	Asia	<5	2006	0<br>(0to0)
Incidence	Asia	<5	2007	0<br>(0to0)
Incidence	Asia	<5	2008	0<br>(0to0)
Incidence	Asia	<5	2009	0<br>(0to0)
Incidence	Asia	<5	2010	0<br>(0to0)
Incidence	Asia	<5	2011	0<br>(0to0)
Incidence	Asia	<5	2012	0<br>(0to0)
Incidence	Asia	<5	2013	0<br>(0to0)
Incidence	Asia	<5	2014	0<br>(0to0)
Incidence	Asia	<5	2015	0<br>(0to0)

Incidence	Asia	<5	2016	0<br>(0to0)
Incidence	Asia	<5	2017	0<br>(0to0)
Incidence	Asia	<5	2018	0<br>(0to0)
Incidence	Asia	<5	2019	0<br>(0to0)
Incidence	Asia	<5	2020	0<br>(0to0)
Incidence	Asia	<5	2021	0<br>(0to0)
Incidence	Asia	5 to 9	1990	0<br>(0to0)
Incidence	Asia	5 to 9	1991	0<br>(0to0)
Incidence	Asia	5 to 9	1992	0<br>(0to0)
Incidence	Asia	5 to 9	1993	0<br>(0to0)
Incidence	Asia	5 to 9	1994	0<br>(0to0)
Incidence	Asia	5 to 9	1995	0<br>(0to0)

Incidence	Asia	5 to 9	1996	0<br>(0to0)
Incidence	Asia	5 to 9	1997	0<br>(0to0)
Incidence	Asia	5 to 9	1998	0<br>(0to0)
Incidence	Asia	5 to 9	1999	0<br>(0to0)
Incidence	Asia	5 to 9	2000	0<br>(0to0)
Incidence	Asia	5 to 9	2001	0<br>(0to0)
Incidence	Asia	5 to 9	2002	0<br>(0to0)
Incidence	Asia	5 to 9	2003	0<br>(0to0)
Incidence	Asia	5 to 9	2004	0<br>(0to0)
Incidence	Asia	5 to 9	2005	0<br>(0to0)
Incidence	Asia	5 to 9	2006	0<br>(0to0)
Incidence	Asia	5 to 9	2007	0<br>(0to0)

Incidence	Asia	5 to 9	2008	0<br>(0to0)
Incidence	Asia	5 to 9	2009	0<br>(0to0)
Incidence	Asia	5 to 9	2010	0<br>(0to0)
Incidence	Asia	5 to 9	2011	0<br>(0to0)
Incidence	Asia	5 to 9	2012	0<br>(0to0)
Incidence	Asia	5 to 9	2013	0<br>(0to0)
Incidence	Asia	5 to 9	2014	0<br>(0to0)
Incidence	Asia	5 to 9	2015	0<br>(0to0)
Incidence	Asia	5 to 9	2016	0<br>(0to0)
Incidence	Asia	5 to 9	2017	0<br>(0to0)
Incidence	Asia	5 to 9	2018	0<br>(0to0)
Incidence	Asia	5 to 9	2019	0<br>(0to0)

Incidence	Asia	5 to 9	2020	0<br>(0to0)
Incidence	Asia	5 to 9	2021	0<br>(0to0)
Incidence	Asia	10 to 14	1990	0<br>(0to0)
Incidence	Asia	10 to 14	1991	0<br>(0to0)
Incidence	Asia	10 to 14	1992	0<br>(0to0)
Incidence	Asia	10 to 14	1993	0<br>(0to0)
Incidence	Asia	10 to 14	1994	0<br>(0to0)
Incidence	Asia	10 to 14	1995	0<br>(0to0)
Incidence	Asia	10 to 14	1996	0<br>(0to0)
Incidence	Asia	10 to 14	1997	0<br>(0to0)
Incidence	Asia	10 to 14	1998	0<br>(0to0)
Incidence	Asia	10 to 14	1999	0<br>(0to0)

Incidence	Asia	10 to 14	2000	0<br>(0to0)
Incidence	Asia	10 to 14	2001	0<br>(0to0)
Incidence	Asia	10 to 14	2002	0<br>(0to0)
Incidence	Asia	10 to 14	2003	0<br>(0to0)
Incidence	Asia	10 to 14	2004	0<br>(0to0)
Incidence	Asia	10 to 14	2005	0<br>(0to0)
Incidence	Asia	10 to 14	2006	0<br>(0to0)
Incidence	Asia	10 to 14	2007	0<br>(0to0)
Incidence	Asia	10 to 14	2008	0<br>(0to0)
Incidence	Asia	10 to 14	2009	0<br>(0to0)
Incidence	Asia	10 to 14	2010	0<br>(0to0)
Incidence	Asia	10 to 14	2011	0<br>(0to0)

Incidence	Asia	10 to 14	2012	0<br>(0to0)
Incidence	Asia	10 to 14	2013	0<br>(0to0)
Incidence	Asia	10 to 14	2014	0<br>(0to0)
Incidence	Asia	10 to 14	2015	0<br>(0to0)
Incidence	Asia	10 to 14	2016	0<br>(0to0)
Incidence	Asia	10 to 14	2017	0<br>(0to0)
Incidence	Asia	10 to 14	2018	0<br>(0to0)
Incidence	Asia	10 to 14	2019	0<br>(0to0)
Incidence	Asia	10 to 14	2020	0<br>(0to0)
Incidence	Asia	10 to 14	2021	0<br>(0to0)
Incidence	Asia	15 to 19	1990	571.81<br>(504.8to654.78)
Incidence	Asia	15 to 19	1991	586.45<br>(517.83to651.23)

Incidence	Asia	15 to 19	1992	601.8 (530.87to675.42)
Incidence	Asia	15 to 19	1993	617.36 (545.87to696.69)
Incidence	Asia	15 to 19	1994	641.13 (570.12to720.02)
Incidence	Asia	15 to 19	1995	657.95 (580.79to744.59)
Incidence	Asia	15 to 19	1996	681.53 (602.52to766.26)
Incidence	Asia	15 to 19	1997	710.06 (623.24to793.19)
Incidence	Asia	15 to 19	1998	739.46 (654.07to820.69)
Incidence	Asia	15 to 19	1999	783.35 (699.4to878.04)
Incidence	Asia	15 to 19	2000	821.96 (727.37to928.57)
Incidence	Asia	15 to 19	2001	846.46 (754.53to956.2)
Incidence	Asia	15 to 19	2002	867.71 (766.05to974.92)
Incidence	Asia	15 to 19	2003	899.8 (799.03to1014.35)

Incidence	Asia	15 to 19	2004	929.49 (819.96to1040.28)
Incidence	Asia	15 to 19	2005	960.73 (851.17to1081.78)
Incidence	Asia	15 to 19	2006	974.47 (860.92to1103.47)
Incidence	Asia	15 to 19	2007	989.51 (872.87to1114.13)
Incidence	Asia	15 to 19	2008	1022.82 (897.03to1158.44)
Incidence	Asia	15 to 19	2009	1048.1 (913.81to1189.24)
Incidence	Asia	15 to 19	2010	1073.11 (929.89to1215.3)
Incidence	Asia	15 to 19	2011	1113.27 (961.73to1282.13)
Incidence	Asia	15 to 19	2012	1129.14 (949.84to1335.42)
Incidence	Asia	15 to 19	2013	1160.04 (979.33to1366.12)
Incidence	Asia	15 to 19	2014	1183.61 (975.33to1415.9)
Incidence	Asia	15 to 19	2015	1200.13 (972.16to1454.58)

Incidence	Asia	15 to 19	2016	1224.43 (977.45to1488.4)
Incidence	Asia	15 to 19	2017	1264.88 (1012.95to1533.58)
Incidence	Asia	15 to 19	2018	1279.33 (1005.65to1597.33)
Incidence	Asia	15 to 19	2019	1320.33 (1022.62to1666.92)
Incidence	Asia	15 to 19	2020	1410.64 (1064.26to1782.93)
Incidence	Asia	15 to 19	2021	1452.76 (1120.43to1814.71)
Incidence	Asia	20 to 24	1990	1418.93 (1226to1662.97)
Incidence	Asia	20 to 24	1991	1458.54 (1264.91to1700.5)
Incidence	Asia	20 to 24	1992	1495.66 (1287.57to1779.89)
Incidence	Asia	20 to 24	1993	1545.17 (1310.99to1829.81)
Incidence	Asia	20 to 24	1994	1597.41 (1373.83to1882.69)
Incidence	Asia	20 to 24	1995	1635.79 (1400.86to1940.37)

Incidence	Asia	20 to 24	1996	1679.33 (1441.76to2005.44)
Incidence	Asia	20 to 24	1997	1734.28 (1502.61to2013.77)
Incidence	Asia	20 to 24	1998	1794.75 (1572.19to2048.51)
Incidence	Asia	20 to 24	1999	1854.09 (1594.57to2148.16)
Incidence	Asia	20 to 24	2000	1919.03 (1664.83to2234.13)
Incidence	Asia	20 to 24	2001	1960.97 (1726.52to2242.76)
Incidence	Asia	20 to 24	2002	2013.68 (1802.65to2278.94)
Incidence	Asia	20 to 24	2003	2092.06 (1880.67to2325.83)
Incidence	Asia	20 to 24	2004	2167.35 (1942.74to2401.77)
Incidence	Asia	20 to 24	2005	2268.92 (2056.06to2504.29)
Incidence	Asia	20 to 24	2006	2375.91 (2144.63to2622.29)
Incidence	Asia	20 to 24	2007	2507.76 (2285.86to2784.45)

Incidence	Asia	20 to 24	2008	2631.68 (2375.24to2921.62)
Incidence	Asia	20 to 24	2009	2765.94 (2509.01to3041.49)
Incidence	Asia	20 to 24	2010	2912.84 (2636.04to3217.14)
Incidence	Asia	20 to 24	2011	3052.61 (2780.04to3363.94)
Incidence	Asia	20 to 24	2012	3123.94 (2852.35to3440.14)
Incidence	Asia	20 to 24	2013	3192.34 (2859.25to3570.83)
Incidence	Asia	20 to 24	2014	3230.31 (2742.23to3752.66)
Incidence	Asia	20 to 24	2015	3268.16 (2701.95to3869.66)
Incidence	Asia	20 to 24	2016	3328.93 (2720.49to3954.54)
Incidence	Asia	20 to 24	2017	3369.61 (2803.69to3951.6)
Incidence	Asia	20 to 24	2018	3417.25 (2787.52to4074.36)
Incidence	Asia	20 to 24	2019	3503.65 (2825.6to4219.89)

Incidence	Asia	20 to 24	2020	3651.08 (2974.74to4469.54)
Incidence	Asia	20 to 24	2021	3727.12 (3056.04to4517.46)
Incidence	Asia	25 to 29	1990	4489.41 (3869.61to5311.39)
Incidence	Asia	25 to 29	1991	4765.32 (4133.91to5615.42)
Incidence	Asia	25 to 29	1992	4968.77 (4224.97to5909.25)
Incidence	Asia	25 to 29	1993	5138.8 (4356.47to6147.12)
Incidence	Asia	25 to 29	1994	5360.12 (4586.4to6345.65)
Incidence	Asia	25 to 29	1995	5558.04 (4765.53to6542.42)
Incidence	Asia	25 to 29	1996	5726.55 (4915.28to6838.83)
Incidence	Asia	25 to 29	1997	5868.95 (5028.27to6930.14)
Incidence	Asia	25 to 29	1998	6146.11 (5346.64to7142.56)
Incidence	Asia	25 to 29	1999	6314.77 (5379.63to7431.36)

Incidence	Asia	25 to 29	2000	6472.76 (5568.75to7661.16)
Incidence	Asia	25 to 29	2001	6526.69 (5620.74to7752.53)
Incidence	Asia	25 to 29	2002	6630.53 (5829.3to7657.04)
Incidence	Asia	25 to 29	2003	6802.46 (6189.79to7565.75)
Incidence	Asia	25 to 29	2004	6839.86 (6222.01to7538.53)
Incidence	Asia	25 to 29	2005	6944.41 (6418.6to7565.28)
Incidence	Asia	25 to 29	2006	7122.81 (6609to7795.76)
Incidence	Asia	25 to 29	2007	7442.77 (6918.76to8126.22)
Incidence	Asia	25 to 29	2008	7921.44 (7271.66to8623.16)
Incidence	Asia	25 to 29	2009	8607.29 (7833.43to9412.05)
Incidence	Asia	25 to 29	2010	9200.36 (8365.16to10027.91)
Incidence	Asia	25 to 29	2011	9710.16 (8933.15to10559.02)

Incidence	Asia	25 to 29	2012	10130.55 (9340.48to10966.25)
Incidence	Asia	25 to 29	2013	10633.9 (9788.58to11678.17)
Incidence	Asia	25 to 29	2014	10802.49 (9848.87to11918.1)
Incidence	Asia	25 to 29	2015	10899.29 (9826.09to12210.21)
Incidence	Asia	25 to 29	2016	11195.01 (9985.21to12478.77)
Incidence	Asia	25 to 29	2017	11559.37 (10138.64to13009.39)
Incidence	Asia	25 to 29	2018	11564.55 (10075.74to13221.45)
Incidence	Asia	25 to 29	2019	11733.94 (10167.87to13436.03)
Incidence	Asia	25 to 29	2020	11798.4 (10174.73to13578.08)
Incidence	Asia	25 to 29	2021	11821 (10272.61to13623.25)
Incidence	Asia	30 to 34	1990	10032.43 (8813.79to11614.93)
Incidence	Asia	30 to 34	1991	10266.06 (9154.02to11779.21)

Incidence	Asia	30 to 34	1992	10621.15 (9373.51to12254.57)
Incidence	Asia	30 to 34	1993	11173.19 (9845.11to13060.81)
Incidence	Asia	30 to 34	1994	11873.97 (10402.54to13836.3)
Incidence	Asia	30 to 34	1995	12713.54 (11102.8to14703.72)
Incidence	Asia	30 to 34	1996	13497.51 (11940.71to15551.51)
Incidence	Asia	30 to 34	1997	14121.87 (12338.88to16316.28)
Incidence	Asia	30 to 34	1998	14725.2 (12984.63to16837)
Incidence	Asia	30 to 34	1999	15300.71 (13340.58to17542.69)
Incidence	Asia	30 to 34	2000	15832.61 (13808.32to18291.9)
Incidence	Asia	30 to 34	2001	16050.54 (14215.83to18395.31)
Incidence	Asia	30 to 34	2002	16484.81 (14890.84to18499.62)
Incidence	Asia	30 to 34	2003	17314.6 (15898.41to19001.08)

Incidence	Asia	30 to 34	2004	17547.5 (16187.67to19130.03)
Incidence	Asia	30 to 34	2005	17570.53 (16358.57to18873.26)
Incidence	Asia	30 to 34	2006	17589.33 (16265.5to18930.88)
Incidence	Asia	30 to 34	2007	17853.34 (16704.03to19131.31)
Incidence	Asia	30 to 34	2008	18312.79 (17103.06to19771.81)
Incidence	Asia	30 to 34	2009	18959.33 (17729.15to20422.33)
Incidence	Asia	30 to 34	2010	19708.84 (18311.53to21255.03)
Incidence	Asia	30 to 34	2011	20419.27 (18776.06to22102.98)
Incidence	Asia	30 to 34	2012	20789.95 (19076.2to22497.85)
Incidence	Asia	30 to 34	2013	21599.08 (19957.45to23461.26)
Incidence	Asia	30 to 34	2014	22382.57 (20474.42to24341.99)
Incidence	Asia	30 to 34	2015	23142.73 (20958.12to25385.75)

Incidence	Asia	30 to 34	2016	24428.65 (22170.11to26959.2)
Incidence	Asia	30 to 34	2017	26125.29 (23407.01to29074.28)
Incidence	Asia	30 to 34	2018	27305.34 (23948to30844.61)
Incidence	Asia	30 to 34	2019	28469.69 (24759.8to32486.5)
Incidence	Asia	30 to 34	2020	29514.91 (25186.56to33936.46)
Incidence	Asia	30 to 34	2021	29903.81 (26130.26to34355.35)
Incidence	Asia	35 to 39	1990	20298.88 (17989.84to22937.88)
Incidence	Asia	35 to 39	1991	21112.62 (19065.79to23922.3)
Incidence	Asia	35 to 39	1992	21841.29 (19660.74to24528.02)
Incidence	Asia	35 to 39	1993	22457.14 (20259.32to25025.74)
Incidence	Asia	35 to 39	1994	22890.94 (20727.55to25557.17)
Incidence	Asia	35 to 39	1995	23194.5 (21068.7to25450.67)

Incidence	Asia	35 to 39	1996	23983.88 (22054to26097.16)
Incidence	Asia	35 to 39	1997	24934.85 (23263.17to26895.41)
Incidence	Asia	35 to 39	1998	26255.96 (24285.39to28455.1)
Incidence	Asia	35 to 39	1999	28082.93 (25884.72to30514.29)
Incidence	Asia	35 to 39	2000	29915.64 (27509.95to32512.41)
Incidence	Asia	35 to 39	2001	31650.2 (29168.25to34399.15)
Incidence	Asia	35 to 39	2002	33117.25 (30551.37to36211.42)
Incidence	Asia	35 to 39	2003	34055.3 (31540.57to36741.52)
Incidence	Asia	35 to 39	2004	34994.91 (32640.79to37665.34)
Incidence	Asia	35 to 39	2005	36030.1 (33891.99to38732.84)
Incidence	Asia	35 to 39	2006	37084.19 (34797.58to39903.82)
Incidence	Asia	35 to 39	2007	37892.94 (35462.56to40681.82)

Incidence	Asia	35 to 39	2008	38240.55 (35749.96to40837.57)
Incidence	Asia	35 to 39	2009	38757.01 (36367.19to41349.51)
Incidence	Asia	35 to 39	2010	39008.84 (36617.73to41367.73)
Incidence	Asia	35 to 39	2011	39353.2 (36669.12to42407.73)
Incidence	Asia	35 to 39	2012	39911.55 (37125.02to42757.07)
Incidence	Asia	35 to 39	2013	40890.34 (38219.77to43782.82)
Incidence	Asia	35 to 39	2014	41708.14 (38610.74to45075.89)
Incidence	Asia	35 to 39	2015	42769.08 (39230.2to46377.08)
Incidence	Asia	35 to 39	2016	44341.32 (40807.15to48144.64)
Incidence	Asia	35 to 39	2017	45969.07 (42289.24to50226.62)
Incidence	Asia	35 to 39	2018	47399.85 (43351.98to52191.17)
Incidence	Asia	35 to 39	2019	49237.12 (44304.65to54913.04)

Incidence	Asia	35 to 39	2020	51200.87 (45604.15to56928.58)
Incidence	Asia	35 to 39	2021	53261.28 (47465.53to60388.55)
Incidence	Asia	40 to 44	1990	28298.21 (25469.22to31795.78)
Incidence	Asia	40 to 44	1991	30551.98 (27617.79to33991.73)
Incidence	Asia	40 to 44	1992	32486.18 (29599.86to36017.2)
Incidence	Asia	40 to 44	1993	34717.51 (31649.2to38347.99)
Incidence	Asia	40 to 44	1994	36435.85 (33170.3to40332.95)
Incidence	Asia	40 to 44	1995	38905.25 (35554.46to42713.81)
Incidence	Asia	40 to 44	1996	40647.32 (37168.01to44356)
Incidence	Asia	40 to 44	1997	41950.68 (38661.32to45381.69)
Incidence	Asia	40 to 44	1998	42795.98 (39521.25to46659.56)
Incidence	Asia	40 to 44	1999	43763.7 (40190.11to47405.89)

Incidence	Asia	40 to 44	2000	44160.01 (41119.46to48220.57)
Incidence	Asia	40 to 44	2001	44806.43 (41787to47816.1)
Incidence	Asia	40 to 44	2002	46168.73 (43236.52to49450.55)
Incidence	Asia	40 to 44	2003	48825.06 (45668.66to52355.06)
Incidence	Asia	40 to 44	2004	52407.17 (49071.48to56226.75)
Incidence	Asia	40 to 44	2005	56829.7 (53410.93to60661.56)
Incidence	Asia	40 to 44	2006	61808.97 (57792.61to66993.01)
Incidence	Asia	40 to 44	2007	66211.48 (61375.87to71660.32)
Incidence	Asia	40 to 44	2008	68009.59 (63217.83to73501.88)
Incidence	Asia	40 to 44	2009	68368.28 (63941.31to73238.86)
Incidence	Asia	40 to 44	2010	69719.14 (65265.11to74657.62)
Incidence	Asia	40 to 44	2011	71949.54 (67320.52to76726.21)

Incidence	Asia	40 to 44	2012	73179.88 (68208.99to78772.26)
Incidence	Asia	40 to 44	2013	75518.76 (70149.82to81418.73)
Incidence	Asia	40 to 44	2014	78516.15 (72488.1to84658.51)
Incidence	Asia	40 to 44	2015	80430.67 (73701.14to87439.6)
Incidence	Asia	40 to 44	2016	81820.81 (75203.27to89053.51)
Incidence	Asia	40 to 44	2017	82929.45 (75760.92to91529.4)
Incidence	Asia	40 to 44	2018	83835.57 (75899.74to92318.88)
Incidence	Asia	40 to 44	2019	84715.53 (76366.1to93775.92)
Incidence	Asia	40 to 44	2020	85859.82 (77471.28to95579.27)
Incidence	Asia	40 to 44	2021	87841.23 (77840.34to98714.9)
Incidence	Asia	45 to 49	1990	29806.87 (27090.24to33085.11)
Incidence	Asia	45 to 49	1991	31028.16 (28450.03to33954.22)

Incidence	Asia	45 to 49	1992	32817.3 (30141.24to35718.26)
Incidence	Asia	45 to 49	1993	34876.37 (32232.56to38002.29)
Incidence	Asia	45 to 49	1994	37559.37 (34296.57to41296.97)
Incidence	Asia	45 to 49	1995	40553.84 (37334.88to44311.69)
Incidence	Asia	45 to 49	1996	44188.22 (40980.07to48322.58)
Incidence	Asia	45 to 49	1997	46794.17 (43191.32to50768.73)
Incidence	Asia	45 to 49	1998	49321.63 (45595.08to53660.87)
Incidence	Asia	45 to 49	1999	50896.77 (46517.25to55666.19)
Incidence	Asia	45 to 49	2000	53823.94 (49116.96to59677.43)
Incidence	Asia	45 to 49	2001	55843.22 (51512.77to60942.5)
Incidence	Asia	45 to 49	2002	58204.36 (54138.2to63386.72)
Incidence	Asia	45 to 49	2003	60967.97 (56935.57to65661.19)

Incidence	Asia	45 to 49	2004	62830.2 (58711.16to67127.11)
Incidence	Asia	45 to 49	2005	64166.83 (60625.48to68251.09)
Incidence	Asia	45 to 49	2006	65322.89 (61546.04to69584.04)
Incidence	Asia	45 to 49	2007	67991.15 (63852.74to72318.98)
Incidence	Asia	45 to 49	2008	73333.01 (68777.24to77568.49)
Incidence	Asia	45 to 49	2009	80263.22 (75286.7to85876.23)
Incidence	Asia	45 to 49	2010	87317.6 (81786.86to93527.45)
Incidence	Asia	45 to 49	2011	91957.22 (85971.16to98770.98)
Incidence	Asia	45 to 49	2012	93783.42 (87147.29to101649.69)
Incidence	Asia	45 to 49	2013	97816.1 (90731.88to106067.14)
Incidence	Asia	45 to 49	2014	100307.31 (92685.37to109783.74)

Incidence	Asia	45 to 49	2015	101444.85 (91728.47to109379.76)
Incidence	Asia	45 to 49	2016	104683.34 (95483.85to115067.57)
Incidence	Asia	45 to 49	2017	109376.03 (99830.2to121234.94)
Incidence	Asia	45 to 49	2018	113580.78 (102091.1to126077.65)
Incidence	Asia	45 to 49	2019	117228.02 (103954.61to130713.81)
Incidence	Asia	45 to 49	2020	119267.45 (106539.01to134952.95)
Incidence	Asia	45 to 49	2021	120030.54 (106854.7to135133.55)
Incidence	Asia	50 to 54	1990	30298.05 (26838.5to34793.44)
Incidence	Asia	50 to 54	1991	31204.27 (27927.22to35248.14)
Incidence	Asia	50 to 54	1992	32494.51 (29167.45to36621.76)
Incidence	Asia	50 to 54	1993	33766.99 (30391.82to37711.75)

Incidence	Asia	50 to 54	1994	35365.57 (32071.05to39089.31)
Incidence	Asia	50 to 54	1995	37264.25 (33888.58to41016.75)
Incidence	Asia	50 to 54	1996	38804.7 (35859.33to42398.05)
Incidence	Asia	50 to 54	1997	40910.24 (37483.74to44680.35)
Incidence	Asia	50 to 54	1998	43309.93 (39743.59to46982.76)
Incidence	Asia	50 to 54	1999	46561.33 (42503.69to50908.66)
Incidence	Asia	50 to 54	2000	49848.73 (45848.98to54502.19)
Incidence	Asia	50 to 54	2001	53956.77 (49894.21to58984.91)
Incidence	Asia	50 to 54	2002	57405.61 (53187.29to62651.78)
Incidence	Asia	50 to 54	2003	61312.63 (56283.61to67086.31)
Incidence	Asia	50 to 54	2004	64859.78 (60158.1to70455.89)
Incidence	Asia	50 to 54	2005	70325.11 (65586.49to76041.72)

Incidence	Asia	50 to 54	2006	74241.94 (69183.36to79558.77)
Incidence	Asia	50 to 54	2007	77374.08 (72536.06to82956.35)
Incidence	Asia	50 to 54	2008	78280.39 (73537.39to83949.69)
Incidence	Asia	50 to 54	2009	78239.19 (72915.84to83248.05)
Incidence	Asia	50 to 54	2010	79494.94 (74303.61to85044.15)
Incidence	Asia	50 to 54	2011	81492.35 (76633.12to87431.86)
Incidence	Asia	50 to 54	2012	84890.83 (79460.97to90439.59)
Incidence	Asia	50 to 54	2013	90335.97 (83518.22to97854.25)
Incidence	Asia	50 to 54	2014	97273.23 (88693.81to106343.16)
Incidence	Asia	50 to 54	2015	104835.47 (93685.26to116511.71)
Incidence	Asia	50 to 54	2016	111573.9 (100304.55to125317.63)

Incidence	Asia	50 to 54	2017	120225.59 (108098.12to135029.17)
Incidence	Asia	50 to 54	2018	126682.2 (113040.31to141246.29)
Incidence	Asia	50 to 54	2019	131725.26 (116548.98to148596.76)
Incidence	Asia	50 to 54	2020	135629.04 (118816.53to155670.45)
Incidence	Asia	50 to 54	2021	138208.1 (121113.36to159620.02)
Incidence	Asia	55 to 59	1990	28537.49 (25113.92to32447.4)
Incidence	Asia	55 to 59	1991	29790.98 (26875.44to34006.61)
Incidence	Asia	55 to 59	1992	31207.42 (28121.51to35219.93)
Incidence	Asia	55 to 59	1993	32591.52 (29398.55to36092.61)
Incidence	Asia	55 to 59	1994	33874.7 (30888.73to37163.37)
Incidence	Asia	55 to 59	1995	35003.96 (32201.75to38154.48)

Incidence	Asia	55 to 59	1996	35961.98 (33209.13to39377.2)
Incidence	Asia	55 to 59	1997	37229.88 (34456.01to40112.78)
Incidence	Asia	55 to 59	1998	38568.96 (35845.04to41428.44)
Incidence	Asia	55 to 59	1999	40604.75 (37511.73to43664.34)
Incidence	Asia	55 to 59	2000	42028.97 (39036.18to45244.21)
Incidence	Asia	55 to 59	2001	43609.66 (40434.24to46812.72)
Incidence	Asia	55 to 59	2002	45335.43 (41896.95to49360.47)
Incidence	Asia	55 to 59	2003	47484.89 (43373.17to52035.14)
Incidence	Asia	55 to 59	2004	52190.25 (48096.43to57276.8)
Incidence	Asia	55 to 59	2005	57049.63 (52824.14to62216.98)
Incidence	Asia	55 to 59	2006	63014.76 (58334.67to68030.58)
Incidence	Asia	55 to 59	2007	67719.47 (63043.86to73118.61)

Incidence	Asia	55 to 59	2008	72046.23 (67112.91to77410.81)
Incidence	Asia	55 to 59	2009	74307.52 (69544.91to79276.79)
Incidence	Asia	55 to 59	2010	79294.57 (74207.23to84726.29)
Incidence	Asia	55 to 59	2011	82522.18 (76251.24to88342.9)
Incidence	Asia	55 to 59	2012	85553.5 (78533.98to93040.68)
Incidence	Asia	55 to 59	2013	88442.88 (81664.63to95533)
Incidence	Asia	55 to 59	2014	91727.92 (84678.56to98872.89)
Incidence	Asia	55 to 59	2015	94305.27 (85934.63to102726.82)
Incidence	Asia	55 to 59	2016	96832.67 (89214.09to104816.7)
Incidence	Asia	55 to 59	2017	101145.93 (91920.09to111957.03)
Incidence	Asia	55 to 59	2018	109299.83 (97795.57to122212.3)

Incidence	Asia	55 to 59	2019	118645.41 (105036.46to134252.01)
Incidence	Asia	55 to 59	2020	125406.09 (110346.24to144812.39)
Incidence	Asia	55 to 59	2021	133721.83 (115366.75to154327.42)
Incidence	Asia	60 to 64	1990	22468.01 (20080.03to25256.57)
Incidence	Asia	60 to 64	1991	23609.22 (21627.12to26378.14)
Incidence	Asia	60 to 64	1992	24677.65 (22355.08to27578.21)
Incidence	Asia	60 to 64	1993	25921.01 (23596.35to28759.07)
Incidence	Asia	60 to 64	1994	27287.82 (24854.14to30187.23)
Incidence	Asia	60 to 64	1995	28565.73 (26123.33to31430.47)
Incidence	Asia	60 to 64	1996	29931.36 (27532.61to33261.89)
Incidence	Asia	60 to 64	1997	31383.32 (28923.43to34259.62)

Incidence	Asia	60 to 64	1998	32600.61 (29896.87to35206.99)
Incidence	Asia	60 to 64	1999	33637.45 (31150.69to36256)
Incidence	Asia	60 to 64	2000	34524.79 (32159.54to37275.75)
Incidence	Asia	60 to 64	2001	35520.36 (33129.1to37775.87)
Incidence	Asia	60 to 64	2002	36499.19 (34060.74to39526.08)
Incidence	Asia	60 to 64	2003	37821.35 (34642.48to40795.43)
Incidence	Asia	60 to 64	2004	39860.81 (36948.61to42842.56)
Incidence	Asia	60 to 64	2005	42057.76 (39278.88to45197.97)
Incidence	Asia	60 to 64	2006	44843.87 (41834.23to47626.57)
Incidence	Asia	60 to 64	2007	48557.72 (45465.33to51789)
Incidence	Asia	60 to 64	2008	52532.86 (48714.39to56302.49)
Incidence	Asia	60 to 64	2009	57564.19 (53705.62to61659.47)

Incidence	Asia	60 to 64	2010	62838.16 (58384.32to67193.17)
Incidence	Asia	60 to 64	2011	69184.36 (64551.59to74049.39)
Incidence	Asia	60 to 64	2012	72940.12 (67302.13to78750.64)
Incidence	Asia	60 to 64	2013	77891.63 (72204.38to84028.83)
Incidence	Asia	60 to 64	2014	81148.83 (74249.97to88164.53)
Incidence	Asia	60 to 64	2015	85816.53 (77705.27to93426.6)
Incidence	Asia	60 to 64	2016	89529.06 (81436.28to97944.05)
Incidence	Asia	60 to 64	2017	94658.85 (85010.62to105871.74)
Incidence	Asia	60 to 64	2018	98554.81 (88376.06to108873.99)
Incidence	Asia	60 to 64	2019	101187.87 (90038.56to112288.55)
Incidence	Asia	60 to 64	2020	101156.17 (89580.33to114266.44)

Incidence	Asia	60 to 64	2021	102466.86 (91644.54to116590)
Incidence	Asia	65 to 69	1990	17712.6 (15955.94to19610.64)
Incidence	Asia	65 to 69	1991	18668.28 (17083.89to20545.52)
Incidence	Asia	65 to 69	1992	19610.3 (17884.81to21637.16)
Incidence	Asia	65 to 69	1993	20735.26 (18920.19to22923.7)
Incidence	Asia	65 to 69	1994	22242.54 (20529.74to24029.05)
Incidence	Asia	65 to 69	1995	23390.9 (21638.65to25306.85)
Incidence	Asia	65 to 69	1996	24542.41 (22551.01to26515.07)
Incidence	Asia	65 to 69	1997	25715.67 (23781.21to27792.22)
Incidence	Asia	65 to 69	1998	27029.69 (24865.42to29115.55)
Incidence	Asia	65 to 69	1999	28362.07 (26051.51to30529.04)
Incidence	Asia	65 to 69	2000	29852.14 (27532.24to32152.15)

Incidence	Asia	65 to 69	2001	31269.69 (28875.24to33758.79)
Incidence	Asia	65 to 69	2002	32274.95 (29731.8to34830.42)
Incidence	Asia	65 to 69	2003	33510.9 (30668.19to36267.53)
Incidence	Asia	65 to 69	2004	35035.83 (32318.44to37847.63)
Incidence	Asia	65 to 69	2005	36605.72 (33906.43to39359.11)
Incidence	Asia	65 to 69	2006	38220.74 (35486.92to40530.6)
Incidence	Asia	65 to 69	2007	39941.8 (37303.42to42454.88)
Incidence	Asia	65 to 69	2008	41901.6 (38927to44476.8)
Incidence	Asia	65 to 69	2009	44676.37 (41791.17to47470.59)
Incidence	Asia	65 to 69	2010	47415.58 (43984.88to50383.25)
Incidence	Asia	65 to 69	2011	49927.57 (46316.08to53002.93)
Incidence	Asia	65 to 69	2012	52696.36 (48894.31to56542.93)

Incidence	Asia	65 to 69	2013	55821.81 (51105.66to59899.36)
Incidence	Asia	65 to 69	2014	59507 (54196.08to64100.17)
Incidence	Asia	65 to 69	2015	63357.45 (57520.59to68815.27)
Incidence	Asia	65 to 69	2016	69286.68 (62787.16to76054.06)
Incidence	Asia	65 to 69	2017	75819.8 (69104.18to83169.21)
Incidence	Asia	65 to 69	2018	82027.73 (74037.25to90629.85)
Incidence	Asia	65 to 69	2019	86726.62 (76863.77to96474.33)
Incidence	Asia	65 to 69	2020	91891.6 (80825.75to104082.03)
Incidence	Asia	65 to 69	2021	95354.72 (84258.88to107849.62)
Incidence	Asia	70 to 74	1990	12293.71 (11015.88to13561.4)
Incidence	Asia	70 to 74	1991	12920.15 (11769.91to14142.45)
Incidence	Asia	70 to 74	1992	13646.24 (12368.74to14982.88)

Incidence	Asia	70 to 74	1993	14501.86 (13182.96to15895.08)
Incidence	Asia	70 to 74	1994	15735.91 (14418.53to17119.35)
Incidence	Asia	70 to 74	1995	16672.69 (15368.26to18096.8)
Incidence	Asia	70 to 74	1996	17690.44 (16224.07to19072.9)
Incidence	Asia	70 to 74	1997	18680.62 (17257.63to20294.85)
Incidence	Asia	70 to 74	1998	19750.33 (18034.43to21211.3)
Incidence	Asia	70 to 74	1999	20728.46 (18998.1to22273.28)
Incidence	Asia	70 to 74	2000	21569.63 (19840.59to23349.57)
Incidence	Asia	70 to 74	2001	22599.03 (20769.89to24418.71)
Incidence	Asia	70 to 74	2002	23521.41 (21602.61to25416.58)
Incidence	Asia	70 to 74	2003	24604.58 (22434.18to26467.18)
Incidence	Asia	70 to 74	2004	26147.86 (23976.08to28270.19)

Incidence	Asia	70 to 74	2005	27823.4 (25562.41to29964.89)
Incidence	Asia	70 to 74	2006	29697.83 (27317.42to31759.31)
Incidence	Asia	70 to 74	2007	31524.11 (29185.77to33676.69)
Incidence	Asia	70 to 74	2008	33147.45 (30657.88to35422.81)
Incidence	Asia	70 to 74	2009	34427.85 (32013.36to36641.86)
Incidence	Asia	70 to 74	2010	35894.44 (33187.1to38086.33)
Incidence	Asia	70 to 74	2011	37416.89 (34366.21to39909.66)
Incidence	Asia	70 to 74	2012	38721.69 (35539.41to41228.08)
Incidence	Asia	70 to 74	2013	40360.77 (36771.94to43101.33)
Incidence	Asia	70 to 74	2014	42173.1 (38235.36to45516.27)
Incidence	Asia	70 to 74	2015	43717.31 (39358.64to46904.59)
Incidence	Asia	70 to 74	2016	45751.7 (41278.55to49856.04)

Incidence	Asia	70 to 74	2017	49339.55 (44561.61to53907.3)
Incidence	Asia	70 to 74	2018	53216.14 (47862.13to58154.41)
Incidence	Asia	70 to 74	2019	57982.58 (51804.48to63334.65)
Incidence	Asia	70 to 74	2020	61593.43 (54670.23to68980.43)
Incidence	Asia	70 to 74	2021	66692.48 (58947.82to74292.21)
Incidence	Asia	75 to 79	1990	8117.89 (7249.72to8995.27)
Incidence	Asia	75 to 79	1991	8485.94 (7719.02to9394.85)
Incidence	Asia	75 to 79	1992	8947.94 (8088.25to9846.31)
Incidence	Asia	75 to 79	1993	9428.76 (8553.91to10361.15)
Incidence	Asia	75 to 79	1994	10061.5 (9177.71to10932.01)
Incidence	Asia	75 to 79	1995	10763.16 (9787.13to11678.6)
Incidence	Asia	75 to 79	1996	11379.65 (10353.53to12309.54)

Incidence	Asia	75 to 79	1997	12038.39 (11007.99to13044.4)
Incidence	Asia	75 to 79	1998	12727.12 (11633.64to13667.85)
Incidence	Asia	75 to 79	1999	13541.65 (12367.77to14554.51)
Incidence	Asia	75 to 79	2000	14266.04 (12897.3to15426.84)
Incidence	Asia	75 to 79	2001	14962.2 (13481.12to16216.1)
Incidence	Asia	75 to 79	2002	15622.71 (14002.52to16808.27)
Incidence	Asia	75 to 79	2003	16346.52 (14698.31to17623.2)
Incidence	Asia	75 to 79	2004	17388.47 (15622.22to18747.6)
Incidence	Asia	75 to 79	2005	18565.73 (16760.17to19909.89)
Incidence	Asia	75 to 79	2006	19568.54 (17695.27to20881.1)
Incidence	Asia	75 to 79	2007	20761.09 (18825.32to22166.65)
Incidence	Asia	75 to 79	2008	22172.29 (20141.41to23677.38)

Incidence	Asia	75 to 79	2009	23608.31 (21375.65to25414.86)
Incidence	Asia	75 to 79	2010	25170.28 (22729.81to26962.25)
Incidence	Asia	75 to 79	2011	26584.31 (23683.3to28656.21)
Incidence	Asia	75 to 79	2012	27590.19 (24588.27to29735.98)
Incidence	Asia	75 to 79	2013	28957.48 (25863.5to31249.09)
Incidence	Asia	75 to 79	2014	30051.41 (26618.07to32532.8)
Incidence	Asia	75 to 79	2015	30934.84 (27214.84to33682.21)
Incidence	Asia	75 to 79	2016	31981.89 (28348.01to34941.76)
Incidence	Asia	75 to 79	2017	33607.19 (29537.07to36972.53)
Incidence	Asia	75 to 79	2018	35490.31 (30732.05to38963.43)
Incidence	Asia	75 to 79	2019	37512.32 (32498.33to41598.85)
Incidence	Asia	75 to 79	2020	38245.92 (32865.28to42804.83)

Incidence	Asia	75 to 79	2021	39892.09 (34236.64to44833.54)
Incidence	Asia	80 to 84	1990	4486.6 (3924.96to4912.81)
Incidence	Asia	80 to 84	1991	4744.06 (4202.75to5201.37)
Incidence	Asia	80 to 84	1992	5101.48 (4485.47to5584.76)
Incidence	Asia	80 to 84	1993	5476.34 (4733.04to6032.63)
Incidence	Asia	80 to 84	1994	5949.55 (5162.32to6562.2)
Incidence	Asia	80 to 84	1995	6491.45 (5648.09to7180.22)
Incidence	Asia	80 to 84	1996	6822.98 (5886.28to7523.23)
Incidence	Asia	80 to 84	1997	7086.64 (6128.11to7861.96)
Incidence	Asia	80 to 84	1998	7402.99 (6355.78to8201.56)
Incidence	Asia	80 to 84	1999	7691.53 (6624.98to8560.14)
Incidence	Asia	80 to 84	2000	8045.51 (6967.42to8907.51)

Incidence	Asia	80 to 84	2001	8443.56 (7208.5to9308.01)
Incidence	Asia	80 to 84	2002	8868.1 (7569.4to9673.63)
Incidence	Asia	80 to 84	2003	9409.63 (7986.17to10270.08)
Incidence	Asia	80 to 84	2004	10182.46 (8673.78to11127.05)
Incidence	Asia	80 to 84	2005	10961.74 (9485.64to11904.96)
Incidence	Asia	80 to 84	2006	11743.62 (9978.01to12841.31)
Incidence	Asia	80 to 84	2007	12626.89 (10649.64to13921.83)
Incidence	Asia	80 to 84	2008	13512.4 (11441.55to14760.04)
Incidence	Asia	80 to 84	2009	14276.33 (12055.85to15597.9)
Incidence	Asia	80 to 84	2010	15247.75 (13057.61to16661.05)
Incidence	Asia	80 to 84	2011	16628.53 (14117.78to18328.9)
Incidence	Asia	80 to 84	2012	17378.56 (14720.99to19157.04)

Incidence	Asia	80 to 84	2013	17699.1 (14982.72to19773.73)
Incidence	Asia	80 to 84	2014	18400.68 (15189.9to21014.29)
Incidence	Asia	80 to 84	2015	19639.34 (16214.24to22009.78)
Incidence	Asia	80 to 84	2016	21067.75 (17566.98to23503.75)
Incidence	Asia	80 to 84	2017	21944.5 (17914.42to24902.93)
Incidence	Asia	80 to 84	2018	23110.65 (19212.4to26140.86)
Incidence	Asia	80 to 84	2019	24429.95 (20364.91to27440.24)
Incidence	Asia	80 to 84	2020	24856.93 (20649.28to28108.94)
Incidence	Asia	80 to 84	2021	25564.56 (20493.92to29233.6)
Incidence	Asia	85 to 89	1990	2158.15 (1803.48to2395.81)
Incidence	Asia	85 to 89	1991	2300.29 (1935.11to2547.34)
Incidence	Asia	85 to 89	1992	2489.99 (2121.13to2787.64)

Incidence	Asia	85 to 89	1993	2680.56 (2230.18to3000.62)
Incidence	Asia	85 to 89	1994	2951.39 (2460.83to3303.99)
Incidence	Asia	85 to 89	1995	3277.1 (2767.78to3696.56)
Incidence	Asia	85 to 89	1996	3495.6 (2919.98to3937.51)
Incidence	Asia	85 to 89	1997	3653.1 (3029.71to4120.85)
Incidence	Asia	85 to 89	1998	3907.03 (3202.31to4388.58)
Incidence	Asia	85 to 89	1999	4194.26 (3455.19to4751.44)
Incidence	Asia	85 to 89	2000	4426.75 (3647.52to4987.2)
Incidence	Asia	85 to 89	2001	4651.78 (3797.2to5237.72)
Incidence	Asia	85 to 89	2002	4850.56 (3982.41to5422.69)
Incidence	Asia	85 to 89	2003	5079.24 (4139.22to5698.92)
Incidence	Asia	85 to 89	2004	5390.98 (4403.33to6005.49)

Incidence	Asia	85 to 89	2005	5792.53 (4724.34to6464.85)
Incidence	Asia	85 to 89	2006	6168.91 (5003.34to6900.55)
Incidence	Asia	85 to 89	2007	6735.82 (5445.65to7601.96)
Incidence	Asia	85 to 89	2008	7304.61 (5839.6to8183.34)
Incidence	Asia	85 to 89	2009	7808.14 (6186.7to8727.76)
Incidence	Asia	85 to 89	2010	8476.59 (6553.89to9555.19)
Incidence	Asia	85 to 89	2011	9264.27 (7268.97to10491.61)
Incidence	Asia	85 to 89	2012	9889.66 (7686.62to11212.3)
Incidence	Asia	85 to 89	2013	10406.44 (7940.74to11779.34)
Incidence	Asia	85 to 89	2014	11026.72 (8428.43to12764.06)
Incidence	Asia	85 to 89	2015	11945.11 (9084.33to13566.07)
Incidence	Asia	85 to 89	2016	12884.61 (9870.97to14755.74)

Incidence	Asia	85 to 89	2017	13443.22 (10194.2to15442.94)
Incidence	Asia	85 to 89	2018	14332.45 (10997.57to16416.08)
Incidence	Asia	85 to 89	2019	15371 (11834.69to17700.42)
Incidence	Asia	85 to 89	2020	15602.14 (11973.77to18037.12)
Incidence	Asia	85 to 89	2021	16491.11 (12495.83to19142.56)
Incidence	Asia	90 to 94	1990	526.65 (431.88to596.22)
Incidence	Asia	90 to 94	1991	571.14 (470.78to643.59)
Incidence	Asia	90 to 94	1992	629.55 (512.98to708.75)
Incidence	Asia	90 to 94	1993	701.43 (568.77to793.74)
Incidence	Asia	90 to 94	1994	787.4 (633.63to896.45)
Incidence	Asia	90 to 94	1995	905.11 (718.9to1041.73)
Incidence	Asia	90 to 94	1996	966.36 (767.11to1112.84)

Incidence	Asia	90 to 94	1997	1020.99 (808.56to1171.83)
Incidence	Asia	90 to 94	1998	1110.39 (881.56to1282.12)
Incidence	Asia	90 to 94	1999	1207.97 (953.83to1397.12)
Incidence	Asia	90 to 94	2000	1297.38 (1024.02to1494.63)
Incidence	Asia	90 to 94	2001	1400.31 (1101.01to1614.44)
Incidence	Asia	90 to 94	2002	1507.12 (1187.37to1727.64)
Incidence	Asia	90 to 94	2003	1606.19 (1271.36to1831.1)
Incidence	Asia	90 to 94	2004	1738.7 (1375.36to1979.48)
Incidence	Asia	90 to 94	2005	1880.33 (1489.42to2144.86)
Incidence	Asia	90 to 94	2006	2026.43 (1584.29to2310.4)
Incidence	Asia	90 to 94	2007	2181.22 (1700.56to2490.33)
Incidence	Asia	90 to 94	2008	2295.8 (1766.41to2617.9)

Incidence	Asia	90 to 94	2009	2380.37 (1828.81to2714.02)
Incidence	Asia	90 to 94	2010	2605.39 (1973.03to2988.18)
Incidence	Asia	90 to 94	2011	2850.87 (2170.49to3283.36)
Incidence	Asia	90 to 94	2012	3085.03 (2350to3567.03)
Incidence	Asia	90 to 94	2013	3385.77 (2542.24to3920.38)
Incidence	Asia	90 to 94	2014	3685.73 (2753.34to4305.27)
Incidence	Asia	90 to 94	2015	3993.17 (2972.96to4639.78)
Incidence	Asia	90 to 94	2016	4404.37 (3296.25to5121.74)
Incidence	Asia	90 to 94	2017	4703.11 (3503.51to5517.67)
Incidence	Asia	90 to 94	2018	5058.68 (3738.43to5902.58)
Incidence	Asia	90 to 94	2019	5531.67 (4074.34to6446.28)
Incidence	Asia	90 to 94	2020	5620.79 (4149.39to6595.35)

Incidence	Asia	90 to 94	2021	5973.06 (4337.17to6979.19)

Supplementary Table S2. Age groups and ASDR of breast cancer in Asia between 1990–2021.

Measure	Location	Age	Year	DALYs
DALYs (Disability-Adjusted Life Years)	Asia	<5	1991	0 (0to0)
DALYs (Disability-Adjusted Life Years)	Asia	5 to 9	1991	0 (0to0)
DALYs (Disability-Adjusted Life Years)	Asia	10 to 14	1991	0 (0to0)
DALYs (Disability-Adjusted Life Years)	Asia	15 to 19	1991	17205.21 (14971to19471.32)
DALYs (Disability-Adjusted Life Years)	Asia	20 to 24	1991	39795.86 (34109.11to46850.53)
DALYs (Disability-Adjusted Life Years)	Asia	25 to 29	1991	103730.77 (89192.43to122848.27)
DALYs (Disability-Adjusted Life Years)	Asia	30 to 34	1991	230907.26 (203756.64to269012.53)
DALYs (Disability-Adjusted Life Years)	Asia	35 to 39	1991	443579.93 (399102.34to500380.19)

DALYs (Disability-Adjusted Life Years)	Asia	40 to 44	1991	563307.32 (506826.24to629655.67)
DALYs (Disability-Adjusted Life Years)	Asia	45 to 49	1991	574171.73 (524216.08to630567.95)
DALYs (Disability-Adjusted Life Years)	Asia	50 to 54	1991	595105.43 (528176.23to682684.15)
DALYs (Disability-Adjusted Life Years)	Asia	55 to 59	1991	548742.5 (489255.47to632095.98)
DALYs (Disability-Adjusted Life Years)	Asia	60 to 64	1991	379821.3 (344110.57to428428.99)
DALYs (Disability-Adjusted Life Years)	Asia	65 to 69	1991	268198.76 (243718.17to298726.41)
DALYs (Disability-Adjusted Life Years)	Asia	70 to 74	1991	174526.53 (157980.28to194675.65)
DALYs (Disability-Adjusted Life Years)	Asia	75 to 79	1991	102264.78 (92372.8to113663.94)
DALYs (Disability-Adjusted Life Years)	Asia	80 to 84	1991	51573.48 (45515.46to56779.6)
DALYs (Disability-Adjusted Life Years)	Asia	85 to 89	1991	21753.71 (18664.84to24154.81)

DALYs (Disability-Adjusted Life Years)	Asia	90 to 94	1991	6193.27 (5158.86to7006.79)
DALYs (Disability-Adjusted Life Years)	Asia	95 plus	1991	1514.04 (1185.89to1772.09)
DALYs (Disability-Adjusted Life Years)	Asia	<5	1990	0 (0to0)
DALYs (Disability-Adjusted Life Years)	Asia	5 to 9	1990	0 (0to0)
DALYs (Disability-Adjusted Life Years)	Asia	10 to 14	1990	0 (0to0)
DALYs (Disability-Adjusted Life Years)	Asia	15 to 19	1990	16922.36 (14641.11to19671.75)
DALYs (Disability-Adjusted Life Years)	Asia	20 to 24	1990	39249.75 (33590.42to46284.39)
DALYs (Disability-Adjusted Life Years)	Asia	25 to 29	1990	99194.56 (84756.19to118285.99)
DALYs (Disability-Adjusted Life Years)	Asia	30 to 34	1990	228353.3 (197639.67to266441)
DALYs (Disability-Adjusted Life Years)	Asia	35 to 39	1990	432252.67 (381088.21to493063.89)
DALYs (Disability-Adjusted Life Years)	Asia	40 to 44	1990	534061.46 (477131.49to604619.72)

DALYs (Disability-Adjusted Life Years)	Asia	45 to 49	1990	556653.35 (504064.55to619974.06)
DALYs (Disability-Adjusted Life Years)	Asia	50 to 54	1990	585422.62 (510734.52to674475.51)
DALYs (Disability-Adjusted Life Years)	Asia	55 to 59	1990	531714.64 (465085.39to609619.48)
DALYs (Disability-Adjusted Life Years)	Asia	60 to 64	1990	366424.61 (325999.32to415452.13)
DALYs (Disability-Adjusted Life Years)	Asia	65 to 69	1990	258786.85 (230935.87to289488.32)
DALYs (Disability-Adjusted Life Years)	Asia	70 to 74	1990	168191 (149743.02to186639.6)
DALYs (Disability-Adjusted Life Years)	Asia	75 to 79	1990	99172.75 (88218to111151.29)
DALYs (Disability-Adjusted Life Years)	Asia	80 to 84	1990	49517.59 (43464.32to54686.92)
DALYs (Disability-Adjusted Life Years)	Asia	85 to 89	1990	20591.24 (17592.68to23062.81)
DALYs (Disability-Adjusted Life Years)	Asia	90 to 94	1990	5756.61 (4764.22to6521.82)
DALYs (Disability-Adjusted Life Years)	Asia	95 plus	1990	1471.78 (1149.2to1716.91)

DALYs (Disability-Adjusted Life Years)	Asia	<5	1994	0<br>(0to0)
DALYs (Disability-Adjusted Life Years)	Asia	5 to 9	1994	0<br>(0to0)
DALYs (Disability-Adjusted Life Years)	Asia	10 to 14	1994	0<br>(0to0)
DALYs (Disability-Adjusted Life Years)	Asia	15 to 19	1994	18514.38<br>(16153.83to21305.04)
DALYs (Disability-Adjusted Life Years)	Asia	20 to 24	1994	42516.83<br>(36250.29to50252.06)
DALYs (Disability-Adjusted Life Years)	Asia	25 to 29	1994	112437.82<br>(95622.04to134264.21)
DALYs (Disability-Adjusted Life Years)	Asia	30 to 34	1994	258147.26<br>(224004.06to302327.58)
DALYs (Disability-Adjusted Life Years)	Asia	35 to 39	1994	459912.83<br>(414581.25to517414.28)
DALYs (Disability-Adjusted Life Years)	Asia	40 to 44	1994	647554.4<br>(584414.66to719647.22)
DALYs (Disability-Adjusted Life Years)	Asia	45 to 49	1994	650058.64<br>(592973.18to718748.87)
DALYs (Disability-Adjusted Life Years)	Asia	50 to 54	1994	642000.02<br>(575286.58to718317.68)

DALYs (Disability-Adjusted Life Years)	Asia	55 to 59	1994	601850.28 (544488.69to665751.54)
DALYs (Disability-Adjusted Life Years)	Asia	60 to 64	1994	423136.56 (382216.53to475881.72)
DALYs (Disability-Adjusted Life Years)	Asia	65 to 69	1994	307499.18 (283105.69to337262.23)
DALYs (Disability-Adjusted Life Years)	Asia	70 to 74	1994	203928.41 (186656.09to223655.58)
DALYs (Disability-Adjusted Life Years)	Asia	75 to 79	1994	117701.58 (107032.28to127987.47)
DALYs (Disability-Adjusted Life Years)	Asia	80 to 84	1994	62496.68 (54853.6to68909.19)
DALYs (Disability-Adjusted Life Years)	Asia	85 to 89	1994	26996.44 (22920.81to30377.14)
DALYs (Disability-Adjusted Life Years)	Asia	90 to 94	1994	8361.26 (6796.21to9552.06)
DALYs (Disability-Adjusted Life Years)	Asia	95 plus	1994	2006.42 (1535.77to2380.72)
DALYs (Disability-Adjusted Life Years)	Asia	<5	1993	0 (0to0)
DALYs (Disability-Adjusted Life Years)	Asia	5 to 9	1993	0 (0to0)

DALYs (Disability-Adjusted Life Years)	Asia	10 to 14	1993	0 (0to0)
DALYs (Disability-Adjusted Life Years)	Asia	15 to 19	1993	17881.55 (15464.98to20530.78)
DALYs (Disability-Adjusted Life Years)	Asia	20 to 24	1993	41433.81 (35199.57to49666.99)
DALYs (Disability-Adjusted Life Years)	Asia	25 to 29	1993	109484.56 (92745.9to132165.13)
DALYs (Disability-Adjusted Life Years)	Asia	30 to 34	1993	246193.27 (213106.19to289434.51)
DALYs (Disability-Adjusted Life Years)	Asia	35 to 39	1993	458720.71 (409749.59to518060)
DALYs (Disability-Adjusted Life Years)	Asia	40 to 44	1993	623902.3 (565673.15to689892.41)
DALYs (Disability-Adjusted Life Years)	Asia	45 to 49	1993	620467.52 (571522.39to681175.72)
DALYs (Disability-Adjusted Life Years)	Asia	50 to 54	1993	626312.43 (558822.75to703678.69)
DALYs (Disability-Adjusted Life Years)	Asia	55 to 59	1993	584762.49 (523891.81to659268.19)
DALYs (Disability-Adjusted Life Years)	Asia	60 to 64	1993	407695.17 (366205.34to454275.21)

DALYs (Disability-Adjusted Life Years)	Asia	65 to 69	1993	290226.63 (263062.19to322267.95)
DALYs (Disability-Adjusted Life Years)	Asia	70 to 74	1993	190796.05 (172896.17to211006.96)
DALYs (Disability-Adjusted Life Years)	Asia	75 to 79	1993	111152.64 (100094.44to123363.2)
DALYs (Disability-Adjusted Life Years)	Asia	80 to 84	1993	58082.8 (50789.26to63843.53)
DALYs (Disability-Adjusted Life Years)	Asia	85 to 89	1993	24809.69 (21009.19to27744.38)
DALYs (Disability-Adjusted Life Years)	Asia	90 to 94	1993	7472.66 (6129.84to8449.56)
DALYs (Disability-Adjusted Life Years)	Asia	95 plus	1993	1811.44 (1412.25to2118.7)
DALYs (Disability-Adjusted Life Years)	Asia	<5	1992	0 (0to0)
DALYs (Disability-Adjusted Life Years)	Asia	5 to 9	1992	0 (0to0)
DALYs (Disability-Adjusted Life Years)	Asia	10 to 14	1992	0 (0to0)
DALYs (Disability-Adjusted Life Years)	Asia	15 to 19	1992	17522.18 (15228.88to20224.04)

DALYs (Disability-Adjusted Life Years)	Asia	20 to 24	1992	40560.19 (34703.02to48544.83)
DALYs (Disability-Adjusted Life Years)	Asia	25 to 29	1992	107081.31 (91241.08to128895.51)
DALYs (Disability-Adjusted Life Years)	Asia	30 to 34	1992	236736.81 (207880.33to277763.49)
DALYs (Disability-Adjusted Life Years)	Asia	35 to 39	1992	452786.31 (404767.76to509314.72)
DALYs (Disability-Adjusted Life Years)	Asia	40 to 44	1992	591721.66 (539848.71to663961.44)
DALYs (Disability-Adjusted Life Years)	Asia	45 to 49	1992	597112.32 (544399.78to659475.54)
DALYs (Disability-Adjusted Life Years)	Asia	50 to 54	1992	611575.01 (545978.18to694944.74)
DALYs (Disability-Adjusted Life Years)	Asia	55 to 59	1992	567198.23 (507908.61to647494.01)
DALYs (Disability-Adjusted Life Years)	Asia	60 to 64	1992	393540.05 (355596.76to443648.51)
DALYs (Disability-Adjusted Life Years)	Asia	65 to 69	1992	278424.79 (252004.24to311513.71)

DALYs (Disability-Adjusted Life Years)	Asia	70 to 74	1992	182123.46 (164761.19to203088.26)
DALYs (Disability-Adjusted Life Years)	Asia	75 to 79	1992	106625.28 (96298.08to118130.88)
DALYs (Disability-Adjusted Life Years)	Asia	80 to 84	1992	54902.23 (48544.22to60355.16)
DALYs (Disability-Adjusted Life Years)	Asia	85 to 89	1992	23402.18 (20098.98to26154.07)
DALYs (Disability-Adjusted Life Years)	Asia	90 to 94	1992	6785.43 (5637.92to7626.3)
DALYs (Disability-Adjusted Life Years)	Asia	95 plus	1992	1650.75 (1294.44to1927.71)
DALYs (Disability-Adjusted Life Years)	Asia	<5	1997	0 (0to0)
DALYs (Disability-Adjusted Life Years)	Asia	5 to 9	1997	0 (0to0)
DALYs (Disability-Adjusted Life Years)	Asia	10 to 14	1997	0 (0to0)
DALYs (Disability-Adjusted Life Years)	Asia	15 to 19	1997	20097.16 (17316.28to22890.11)
DALYs (Disability-Adjusted Life Years)	Asia	20 to 24	1997	45577.54 (38836.41to53845.71)

DALYs (Disability-Adjusted Life Years)	Asia	25 to 29	1997	118941.09 (100794.25to140980.16)
DALYs (Disability-Adjusted Life Years)	Asia	30 to 34	1997	294672.12 (256642.37to345533.5)
DALYs (Disability-Adjusted Life Years)	Asia	35 to 39	1997	486689.33 (452017.21to527334.94)
DALYs (Disability-Adjusted Life Years)	Asia	40 to 44	1997	717082.19 (658446.82to782458.8)
DALYs (Disability-Adjusted Life Years)	Asia	45 to 49	1997	755829.6 (696022.33to824042.4)
DALYs (Disability-Adjusted Life Years)	Asia	50 to 54	1997	707527.08 (641637.69to786153.21)
DALYs (Disability-Adjusted Life Years)	Asia	55 to 59	1997	632834 (579672.85to691063.68)
DALYs (Disability-Adjusted Life Years)	Asia	60 to 64	1997	466789.89 (425097.03to514977.42)
DALYs (Disability-Adjusted Life Years)	Asia	65 to 69	1997	342666.07 (315836.32to376154.61)
DALYs (Disability-Adjusted Life Years)	Asia	70 to 74	1997	232089.04 (215252.94to253332.06)

DALYs (Disability-Adjusted Life Years)	Asia	75 to 79	1997	134819.04 (123682.12to147198.19)
DALYs (Disability-Adjusted Life Years)	Asia	80 to 84	1997	71317.47 (62528.88to78985.07)
DALYs (Disability-Adjusted Life Years)	Asia	85 to 89	1997	31666.82 (26466.95to35585.11)
DALYs (Disability-Adjusted Life Years)	Asia	90 to 94	1997	10540.33 (8425.22to12103.95)
DALYs (Disability-Adjusted Life Years)	Asia	95 plus	1997	2547.51 (1928.51to3004.8)
DALYs (Disability-Adjusted Life Years)	Asia	<5	1998	0 (0to0)
DALYs (Disability-Adjusted Life Years)	Asia	5 to 9	1998	0 (0to0)
DALYs (Disability-Adjusted Life Years)	Asia	10 to 14	1998	0 (0to0)
DALYs (Disability-Adjusted Life Years)	Asia	15 to 19	1998	20788.22 (18074.35to23709.37)
DALYs (Disability-Adjusted Life Years)	Asia	20 to 24	1998	46875.8 (40152.92to54567.51)
DALYs (Disability-Adjusted Life Years)	Asia	25 to 29	1998	122972 (105550.65to143669.7)

DALYs (Disability-Adjusted Life Years)	Asia	30 to 34	1998	302787.69 (265168.5to350805.99)
DALYs (Disability-Adjusted Life Years)	Asia	35 to 39	1998	506885.69 (466954.73to552008.21)
DALYs (Disability-Adjusted Life Years)	Asia	40 to 44	1998	723394.35 (663552.94to787998.35)
DALYs (Disability-Adjusted Life Years)	Asia	45 to 49	1998	788202.24 (722618.13to858878.16)
DALYs (Disability-Adjusted Life Years)	Asia	50 to 54	1998	735253.71 (667873.77to812416.19)
DALYs (Disability-Adjusted Life Years)	Asia	55 to 59	1998	646917.96 (593925.47to703715.74)
DALYs (Disability-Adjusted Life Years)	Asia	60 to 64	1998	480006.21 (437886.82to526573.97)
DALYs (Disability-Adjusted Life Years)	Asia	65 to 69	1998	357090.37 (325024.38to392344.68)
DALYs (Disability-Adjusted Life Years)	Asia	70 to 74	1998	242960.83 (223875.33to262683.06)
DALYs (Disability-Adjusted Life Years)	Asia	75 to 79	1998	140697.06 (128843.43to152380.74)

DALYs (Disability-Adjusted Life Years)	Asia	80 to 84	1998	73849.21 (64214.95to81236.99)
DALYs (Disability-Adjusted Life Years)	Asia	85 to 89	1998	33322.77 (27670to37169.78)
DALYs (Disability-Adjusted Life Years)	Asia	90 to 94	1998	11315.37 (9088.24to12991.6)
DALYs (Disability-Adjusted Life Years)	Asia	95 plus	1998	2808.72 (2116.68to3287.13)
DALYs (Disability-Adjusted Life Years)	Asia	<5	1995	0 (0to0)
DALYs (Disability-Adjusted Life Years)	Asia	5 to 9	1995	0 (0to0)
DALYs (Disability-Adjusted Life Years)	Asia	10 to 14	1995	0 (0to0)
DALYs (Disability-Adjusted Life Years)	Asia	15 to 19	1995	18877.51 (16274.83to21923.67)
DALYs (Disability-Adjusted Life Years)	Asia	20 to 24	1995	43425.04 (36337.34to51719.75)
DALYs (Disability-Adjusted Life Years)	Asia	25 to 29	1995	114926.55 (98127.04to136850.12)
DALYs (Disability-Adjusted Life Years)	Asia	30 to 34	1995	272905.46 (234647.59to319915.64)

DALYs (Disability-Adjusted Life Years)	Asia	35 to 39	1995	461119.92 (412598.56to510043.86)
DALYs (Disability-Adjusted Life Years)	Asia	40 to 44	1995	682990.91 (619649.38to756416.52)
DALYs (Disability-Adjusted Life Years)	Asia	45 to 49	1995	681939.72 (622047.17to751487.83)
DALYs (Disability-Adjusted Life Years)	Asia	50 to 54	1995	663264.23 (595212.52to743536.51)
DALYs (Disability-Adjusted Life Years)	Asia	55 to 59	1995	613101.42 (559592.46to675450.5)
DALYs (Disability-Adjusted Life Years)	Asia	60 to 64	1995	437034.33 (392127.74to488323.72)
DALYs (Disability-Adjusted Life Years)	Asia	65 to 69	1995	318607.16 (291939.72to347482.46)
DALYs (Disability-Adjusted Life Years)	Asia	70 to 74	1995	212813.82 (194747.34to233272.94)
DALYs (Disability-Adjusted Life Years)	Asia	75 to 79	1995	124048.51 (113122.8to136074.11)
DALYs (Disability-Adjusted Life Years)	Asia	80 to 84	1995	67228.38 (58852.52to74677.85)

DALYs (Disability-Adjusted Life Years)	Asia	85 to 89	1995	29580.41 (25224.91to33338.91)
DALYs (Disability-Adjusted Life Years)	Asia	90 to 94	1995	9526.23 (7753.86to10919.84)
DALYs (Disability-Adjusted Life Years)	Asia	95 plus	1995	2256.21 (1729.95to2685.29)
DALYs (Disability-Adjusted Life Years)	Asia	<5	1996	0 (0to0)
DALYs (Disability-Adjusted Life Years)	Asia	5 to 9	1996	0 (0to0)
DALYs (Disability-Adjusted Life Years)	Asia	10 to 14	1996	0 (0to0)
DALYs (Disability-Adjusted Life Years)	Asia	15 to 19	1996	19429.81 (16818.69to22122.43)
DALYs (Disability-Adjusted Life Years)	Asia	20 to 24	1996	44463.06 (37634.71to52876.3)
DALYs (Disability-Adjusted Life Years)	Asia	25 to 29	1996	117161.23 (99531.67to140193.1)
DALYs (Disability-Adjusted Life Years)	Asia	30 to 34	1996	285930.1 (249847.21to334283.71)
DALYs (Disability-Adjusted Life Years)	Asia	35 to 39	1996	472846.2 (431524.67to518905.33)

DALYs (Disability-Adjusted Life Years)	Asia	40 to 44	1996	704138.74 (642086.68to772378.76)
DALYs (Disability-Adjusted Life Years)	Asia	45 to 49	1996	724378.47 (667277.02to793382.17)
DALYs (Disability-Adjusted Life Years)	Asia	50 to 54	1996	682111.64 (621697.02to756564.65)
DALYs (Disability-Adjusted Life Years)	Asia	55 to 59	1996	621054.31 (566992.91to687447.51)
DALYs (Disability-Adjusted Life Years)	Asia	60 to 64	1996	451481.84 (411354.44to505269.12)
DALYs (Disability-Adjusted Life Years)	Asia	65 to 69	1996	330175.56 (303538.47to360585.86)
DALYs (Disability-Adjusted Life Years)	Asia	70 to 74	1996	222087.74 (204254.72to241030.01)
DALYs (Disability-Adjusted Life Years)	Asia	75 to 79	1996	129267.72 (118787.01to140292.64)
DALYs (Disability-Adjusted Life Years)	Asia	80 to 84	1996	69564.01 (61881.08to76361.36)
DALYs (Disability-Adjusted Life Years)	Asia	85 to 89	1996	30875.2 (26254.73to34690.29)

DALYs (Disability-Adjusted Life Years)	Asia	90 to 94	1996	10081.81 (8141.77to11575.56)
DALYs (Disability-Adjusted Life Years)	Asia	95 plus	1996	2407.86 (1820.99to2861.26)
DALYs (Disability-Adjusted Life Years)	Asia	<5	2002	0 (0to0)
DALYs (Disability-Adjusted Life Years)	Asia	5 to 9	2002	0 (0to0)
DALYs (Disability-Adjusted Life Years)	Asia	10 to 14	2002	0 (0to0)
DALYs (Disability-Adjusted Life Years)	Asia	15 to 19	2002	23488.35 (20544.98to27257.66)
DALYs (Disability-Adjusted Life Years)	Asia	20 to 24	2002	51029.8 (44799.43to58180.58)
DALYs (Disability-Adjusted Life Years)	Asia	25 to 29	2002	128208.62 (110989.54to148952.36)
DALYs (Disability-Adjusted Life Years)	Asia	30 to 34	2002	321656.94 (287254.28to361985.45)
DALYs (Disability-Adjusted Life Years)	Asia	35 to 39	2002	608072.84 (554505.83to670532.87)
DALYs (Disability-Adjusted Life Years)	Asia	40 to 44	2002	747687.87 (697885.28to807813.81)

DALYs (Disability-Adjusted Life Years)	Asia	45 to 49	2002	891317.23 (828817.83to967457.44)
DALYs (Disability-Adjusted Life Years)	Asia	50 to 54	2002	907073.69 (836674.72to994375.39)
DALYs (Disability-Adjusted Life Years)	Asia	55 to 59	2002	719831.73 (661718.64to786851.5)
DALYs (Disability-Adjusted Life Years)	Asia	60 to 64	2002	510905.53 (472920.39to556484.91)
DALYs (Disability-Adjusted Life Years)	Asia	65 to 69	2002	408374.63 (373667to444391.91)
DALYs (Disability-Adjusted Life Years)	Asia	70 to 74	2002	278616.53 (256870.73to301806.45)
DALYs (Disability-Adjusted Life Years)	Asia	75 to 79	2002	165339.37 (150160.93to178967.68)
DALYs (Disability-Adjusted Life Years)	Asia	80 to 84	2002	85878.66 (75347.41to93742.28)
DALYs (Disability-Adjusted Life Years)	Asia	85 to 89	2002	40086.09 (33594.37to44549.83)
DALYs (Disability-Adjusted Life Years)	Asia	90 to 94	2002	14763.62 (12023.27to16814.14)
DALYs (Disability-Adjusted Life Years)	Asia	95 plus	2002	3972.01 (2923.7to4663.5)

DALYs (Disability-Adjusted Life Years)	Asia	<5	2003	0 (0to0)
DALYs (Disability-Adjusted Life Years)	Asia	5 to 9	2003	0 (0to0)
DALYs (Disability-Adjusted Life Years)	Asia	10 to 14	2003	0 (0to0)
DALYs (Disability-Adjusted Life Years)	Asia	15 to 19	2003	24151.23 (21012.36to27565.12)
DALYs (Disability-Adjusted Life Years)	Asia	20 to 24	2003	52131.49 (46511.87to58352.16)
DALYs (Disability-Adjusted Life Years)	Asia	25 to 29	2003	129289.79 (116006.92to145315.78)
DALYs (Disability-Adjusted Life Years)	Asia	30 to 34	2003	331906.07 (301249.29to366294.58)
DALYs (Disability-Adjusted Life Years)	Asia	35 to 39	2003	612573.63 (565205.81to668616.79)
DALYs (Disability-Adjusted Life Years)	Asia	40 to 44	2003	775006.68 (720424.57to831474.87)
DALYs (Disability-Adjusted Life Years)	Asia	45 to 49	2003	915133.84 (853092.69to982076.31)
DALYs (Disability-Adjusted Life Years)	Asia	50 to 54	2003	951411.43 (866732.25to1045228.53)

DALYs (Disability-Adjusted Life Years)	Asia	55 to 59	2003	737652.06 (670208.91to815910.83)
DALYs (Disability-Adjusted Life Years)	Asia	60 to 64	2003	520010.2 (474526.41to565749.84)
DALYs (Disability-Adjusted Life Years)	Asia	65 to 69	2003	416865.85 (379587.06to456584.53)
DALYs (Disability-Adjusted Life Years)	Asia	70 to 74	2003	288201.48 (263311.66to313410.75)
DALYs (Disability-Adjusted Life Years)	Asia	75 to 79	2003	170783.92 (154582.11to185236.76)
DALYs (Disability-Adjusted Life Years)	Asia	80 to 84	2003	90082.65 (78915.06to97393.67)
DALYs (Disability-Adjusted Life Years)	Asia	85 to 89	2003	41636.97 (35195.08to46256.34)
DALYs (Disability-Adjusted Life Years)	Asia	90 to 94	2003	15568.85 (12748.94to17634.06)
DALYs (Disability-Adjusted Life Years)	Asia	95 plus	2003	4323.73 (3209.68to5051.59)
DALYs (Disability-Adjusted Life Years)	Asia	<5	2001	0 (0to0)
DALYs (Disability-Adjusted Life Years)	Asia	5 to 9	2001	0 (0to0)

DALYs (Disability-Adjusted Life Years)	Asia	10 to 14	2001	0 (0to0)
DALYs (Disability-Adjusted Life Years)	Asia	15 to 19	2001	23155.52 (20141.95to26732.93)
DALYs (Disability-Adjusted Life Years)	Asia	20 to 24	2001	50255.27 (43568.02to57597.93)
DALYs (Disability-Adjusted Life Years)	Asia	25 to 29	2001	127968.46 (108918.89to152251.7)
DALYs (Disability-Adjusted Life Years)	Asia	30 to 34	2001	317764.93 (280110.16to364932.91)
DALYs (Disability-Adjusted Life Years)	Asia	35 to 39	2001	589298.9 (537798.77to646365.68)
DALYs (Disability-Adjusted Life Years)	Asia	40 to 44	2001	733010.33 (684395.24to787184.34)
DALYs (Disability-Adjusted Life Years)	Asia	45 to 49	2001	867025.15 (795813.32to948823.36)
DALYs (Disability-Adjusted Life Years)	Asia	50 to 54	2001	864612.46 (793545.71to948345.4)
DALYs (Disability-Adjusted Life Years)	Asia	55 to 59	2001	704059.15 (648939.09to767253.69)

DALYs (Disability-Adjusted Life Years)	Asia	60 to 64	2001	504557.96 (467593.76to543305.58)
DALYs (Disability-Adjusted Life Years)	Asia	65 to 69	2001	399862.2 (368748.56to436930.13)
DALYs (Disability-Adjusted Life Years)	Asia	70 to 74	2001	269213.37 (249707.06to292241.51)
DALYs (Disability-Adjusted Life Years)	Asia	75 to 79	2001	159864.78 (145308.23to173813.74)
DALYs (Disability-Adjusted Life Years)	Asia	80 to 84	2001	82636.3 (72060.17to91432.52)
DALYs (Disability-Adjusted Life Years)	Asia	85 to 89	2001	38817.11 (32393.98to43574.65)
DALYs (Disability-Adjusted Life Years)	Asia	90 to 94	2001	13923.2 (11295.96to15928.02)
DALYs (Disability-Adjusted Life Years)	Asia	95 plus	2001	3620.04 (2697.69to4249.3)
DALYs (Disability-Adjusted Life Years)	Asia	<5	2000	0 (0to0)
DALYs (Disability-Adjusted Life Years)	Asia	5 to 9	2000	0 (0to0)
DALYs (Disability-Adjusted Life Years)	Asia	10 to 14	2000	0 (0to0)

DALYs (Disability-Adjusted Life Years)	Asia	15 to 19	2000	22750.93 (19706.76to26386.5)
DALYs (Disability-Adjusted Life Years)	Asia	20 to 24	2000	49590.44 (42233.69to57668.67)
DALYs (Disability-Adjusted Life Years)	Asia	25 to 29	2000	127744.58 (108764.73to152702.25)
DALYs (Disability-Adjusted Life Years)	Asia	30 to 34	2000	319322.67 (277392.2to371483.57)
DALYs (Disability-Adjusted Life Years)	Asia	35 to 39	2000	563813.4 (513722.58to617571.65)
DALYs (Disability-Adjusted Life Years)	Asia	40 to 44	2000	730356.17 (675750.88to790933.71)
DALYs (Disability-Adjusted Life Years)	Asia	45 to 49	2000	848064.91 (770152.59to938132.74)
DALYs (Disability-Adjusted Life Years)	Asia	50 to 54	2000	814240.61 (741828.44to903077.68)
DALYs (Disability-Adjusted Life Years)	Asia	55 to 59	2000	685586.55 (630184.78to745142.11)
DALYs (Disability-Adjusted Life Years)	Asia	60 to 64	2000	497303.18 (458310.15to539051.03)

DALYs (Disability-Adjusted Life Years)	Asia	65 to 69	2000	385865.3 (354168.69to419854.44)
DALYs (Disability-Adjusted Life Years)	Asia	70 to 74	2000	259456.42 (239866.44to281759.06)
DALYs (Disability-Adjusted Life Years)	Asia	75 to 79	2000	153918.69 (140297.54to167825.38)
DALYs (Disability-Adjusted Life Years)	Asia	80 to 84	2000	79321.76 (69383.39to87627.94)
DALYs (Disability-Adjusted Life Years)	Asia	85 to 89	2000	37040.31 (30998.83to41656.11)
DALYs (Disability-Adjusted Life Years)	Asia	90 to 94	2000	12987.6 (10508.77to14908.56)
DALYs (Disability-Adjusted Life Years)	Asia	95 plus	2000	3283.43 (2442.14to3856.08)
DALYs (Disability-Adjusted Life Years)	Asia	<5	1999	0 (0to0)
DALYs (Disability-Adjusted Life Years)	Asia	5 to 9	1999	0 (0to0)
DALYs (Disability-Adjusted Life Years)	Asia	10 to 14	1999	0 (0to0)
DALYs (Disability-Adjusted Life Years)	Asia	15 to 19	1999	21843.5 (19181.45to24887.99)

DALYs (Disability-Adjusted Life Years)	Asia	20 to 24	1999	48287.09 (41051.11to56600.21)
DALYs (Disability-Adjusted Life Years)	Asia	25 to 29	1999	125688.6 (105850.7to148185.67)
DALYs (Disability-Adjusted Life Years)	Asia	30 to 34	1999	312098.09 (270609.54to361538.95)
DALYs (Disability-Adjusted Life Years)	Asia	35 to 39	1999	536665.29 (490500.43to585615.81)
DALYs (Disability-Adjusted Life Years)	Asia	40 to 44	1999	731392.97 (667509.62to797843.26)
DALYs (Disability-Adjusted Life Years)	Asia	45 to 49	1999	810532.6 (734380.28to890427.87)
DALYs (Disability-Adjusted Life Years)	Asia	50 to 54	1999	776713.63 (703218.15to858539.62)
DALYs (Disability-Adjusted Life Years)	Asia	55 to 59	1999	669510.07 (614208.99to728499.38)
DALYs (Disability-Adjusted Life Years)	Asia	60 to 64	1999	489098.04 (449285.19to530370.34)
DALYs (Disability-Adjusted Life Years)	Asia	65 to 69	1999	370913.45 (341730.08to403926.52)

DALYs (Disability-Adjusted Life Years)	Asia	70 to 74	1999	252327.46 (232200.65to273540.81)
DALYs (Disability-Adjusted Life Years)	Asia	75 to 79	1999	147644.81 (135343.78to160519.2)
DALYs (Disability-Adjusted Life Years)	Asia	80 to 84	1999	76420.76 (66975.03to85023.71)
DALYs (Disability-Adjusted Life Years)	Asia	85 to 89	1999	35324.87 (29711.4to39792.49)
DALYs (Disability-Adjusted Life Years)	Asia	90 to 94	1999	12165.18 (9838.97to13955.24)
DALYs (Disability-Adjusted Life Years)	Asia	95 plus	1999	3039.85 (2286.96to3578.64)
DALYs (Disability-Adjusted Life Years)	Asia	<5	2004	0 (0to0)
DALYs (Disability-Adjusted Life Years)	Asia	5 to 9	2004	0 (0to0)
DALYs (Disability-Adjusted Life Years)	Asia	10 to 14	2004	0 (0to0)
DALYs (Disability-Adjusted Life Years)	Asia	15 to 19	2004	24589.08 (21339.62to28272.66)
DALYs (Disability-Adjusted Life Years)	Asia	20 to 24	2004	53098.22 (47046.82to59858.58)

DALYs (Disability-Adjusted Life Years)	Asia	25 to 29	2004	128095.42 (114424.66to144079.64)
DALYs (Disability-Adjusted Life Years)	Asia	30 to 34	2004	330073 (301339.65to364605.51)
DALYs (Disability-Adjusted Life Years)	Asia	35 to 39	2004	611146.06 (566971.93to659615.19)
DALYs (Disability-Adjusted Life Years)	Asia	40 to 44	2004	807103.02 (754078.36to866451.51)
DALYs (Disability-Adjusted Life Years)	Asia	45 to 49	2004	921266.31 (861191.84to989408.75)
DALYs (Disability-Adjusted Life Years)	Asia	50 to 54	2004	981842.33 (907860.79to1072502.47)
DALYs (Disability-Adjusted Life Years)	Asia	55 to 59	2004	785747.15 (721633.2to865812.88)
DALYs (Disability-Adjusted Life Years)	Asia	60 to 64	2004	531824.66 (493671.95to578106.67)
DALYs (Disability-Adjusted Life Years)	Asia	65 to 69	2004	424376.16 (389257.31to460175.65)
DALYs (Disability-Adjusted Life Years)	Asia	70 to 74	2004	299147.15 (274022.59to325821.91)

DALYs (Disability-Adjusted Life Years)	Asia	75 to 79	2004	177777.79 (162161.03to192759.56)
DALYs (Disability-Adjusted Life Years)	Asia	80 to 84	2004	95338.44 (83307.22to104005.73)
DALYs (Disability-Adjusted Life Years)	Asia	85 to 89	2004	43603.29 (36739.91to48139.13)
DALYs (Disability-Adjusted Life Years)	Asia	90 to 94	2004	16580.39 (13547.6to18747.21)
DALYs (Disability-Adjusted Life Years)	Asia	95 plus	2004	4730.08 (3491.33to5499.38)
DALYs (Disability-Adjusted Life Years)	Asia	<5	2005	0 (0to0)
DALYs (Disability-Adjusted Life Years)	Asia	5 to 9	2005	0 (0to0)
DALYs (Disability-Adjusted Life Years)	Asia	10 to 14	2005	0 (0to0)
DALYs (Disability-Adjusted Life Years)	Asia	15 to 19	2005	25101.23 (21783.99to29009.08)
DALYs (Disability-Adjusted Life Years)	Asia	20 to 24	2005	54591.77 (48694.18to61101.61)
DALYs (Disability-Adjusted Life Years)	Asia	25 to 29	2005	127941.19 (116183.59to142305.14)

DALYs (Disability-Adjusted Life Years)	Asia	30 to 34	2005	323102.72 (297276.76to350010.31)
DALYs (Disability-Adjusted Life Years)	Asia	35 to 39	2005	612520.35 (571846.44to657689.87)
DALYs (Disability-Adjusted Life Years)	Asia	40 to 44	2005	841927.62 (788037.42to896842.14)
DALYs (Disability-Adjusted Life Years)	Asia	45 to 49	2005	916819.64 (863663.01to974539.46)
DALYs (Disability-Adjusted Life Years)	Asia	50 to 54	2005	1030144.31 (956543.9to1116470.23)
DALYs (Disability-Adjusted Life Years)	Asia	55 to 59	2005	826506.77 (762630.47to899017.03)
DALYs (Disability-Adjusted Life Years)	Asia	60 to 64	2005	544785.51 (506192.15to589839.57)
DALYs (Disability-Adjusted Life Years)	Asia	65 to 69	2005	432864.21 (397216.78to467146.55)
DALYs (Disability-Adjusted Life Years)	Asia	70 to 74	2005	310273.73 (285338.99to337236.02)
DALYs (Disability-Adjusted Life Years)	Asia	75 to 79	2005	185505.91 (168968.48to200458.15)

DALYs (Disability-Adjusted Life Years)	Asia	80 to 84	2005	100448.2 (88953.35to108542.91)
DALYs (Disability-Adjusted Life Years)	Asia	85 to 89	2005	46028.36 (38985.66to51124.37)
DALYs (Disability-Adjusted Life Years)	Asia	90 to 94	2005	17614.89 (14369.68to19773.92)
DALYs (Disability-Adjusted Life Years)	Asia	95 plus	2005	5096.46 (3795.58to5927)
DALYs (Disability-Adjusted Life Years)	Asia	<5	2006	0 (0to0)
DALYs (Disability-Adjusted Life Years)	Asia	5 to 9	2006	0 (0to0)
DALYs (Disability-Adjusted Life Years)	Asia	10 to 14	2006	0 (0to0)
DALYs (Disability-Adjusted Life Years)	Asia	15 to 19	2006	25046.74 (21605.9to29214.89)
DALYs (Disability-Adjusted Life Years)	Asia	20 to 24	2006	55875.56 (49834.22to62385.32)
DALYs (Disability-Adjusted Life Years)	Asia	25 to 29	2006	128582.29 (117001.19to142642.09)
DALYs (Disability-Adjusted Life Years)	Asia	30 to 34	2006	317712.34 (294670.74to342684.73)

DALYs (Disability-Adjusted Life Years)	Asia	35 to 39	2006	614082.94 (572847.04to662320.48)
DALYs (Disability-Adjusted Life Years)	Asia	40 to 44	2006	882570.01 (825349.71to947951.43)
DALYs (Disability-Adjusted Life Years)	Asia	45 to 49	2006	911464.06 (861440.63to963542.65)
DALYs (Disability-Adjusted Life Years)	Asia	50 to 54	2006	1053750.3 (980338.63to1135285.27)
DALYs (Disability-Adjusted Life Years)	Asia	55 to 59	2006	880405.6 (816793to951746.84)
DALYs (Disability-Adjusted Life Years)	Asia	60 to 64	2006	565616.87 (527653.55to611008.75)
DALYs (Disability-Adjusted Life Years)	Asia	65 to 69	2006	440515.78 (409713.76to471115.55)
DALYs (Disability-Adjusted Life Years)	Asia	70 to 74	2006	323196.81 (298924.93to347565.4)
DALYs (Disability-Adjusted Life Years)	Asia	75 to 79	2006	191604.22 (176428.81to205714.54)
DALYs (Disability-Adjusted Life Years)	Asia	80 to 84	2006	105597.62 (92415.83to115104.57)

DALYs (Disability-Adjusted Life Years)	Asia	85 to 89	2006	48141.62 (40673.68to53449.02)
DALYs (Disability-Adjusted Life Years)	Asia	90 to 94	2006	18640.4 (15186.98to21031.14)
DALYs (Disability-Adjusted Life Years)	Asia	95 plus	2006	5631.77 (4168.82to6557.81)
DALYs (Disability-Adjusted Life Years)	Asia	<5	2007	0 (0to0)
DALYs (Disability-Adjusted Life Years)	Asia	5 to 9	2007	0 (0to0)
DALYs (Disability-Adjusted Life Years)	Asia	10 to 14	2007	0 (0to0)
DALYs (Disability-Adjusted Life Years)	Asia	15 to 19	2007	25045.23 (21610.02to28973.59)
DALYs (Disability-Adjusted Life Years)	Asia	20 to 24	2007	57549.71 (51712.49to64368.01)
DALYs (Disability-Adjusted Life Years)	Asia	25 to 29	2007	131209.66 (119818.91to145678.49)
DALYs (Disability-Adjusted Life Years)	Asia	30 to 34	2007	316638.26 (294643.13to341034.07)
DALYs (Disability-Adjusted Life Years)	Asia	35 to 39	2007	613188.17 (573267.66to654989.57)

DALYs (Disability-Adjusted Life Years)	Asia	40 to 44	2007	918607.52 (850043.08to993765.63)
DALYs (Disability-Adjusted Life Years)	Asia	45 to 49	2007	927572.01 (876991.9to982528.39)
DALYs (Disability-Adjusted Life Years)	Asia	50 to 54	2007	1072409.28 (1006625.65to1159776.82)
DALYs (Disability-Adjusted Life Years)	Asia	55 to 59	2007	924151.26 (860696.7to994631.57)
DALYs (Disability-Adjusted Life Years)	Asia	60 to 64	2007	596851.25 (558082.4to642375.95)
DALYs (Disability-Adjusted Life Years)	Asia	65 to 69	2007	448868.17 (418926.92to480225.38)
DALYs (Disability-Adjusted Life Years)	Asia	70 to 74	2007	336770.34 (311417.52to363373.95)
DALYs (Disability-Adjusted Life Years)	Asia	75 to 79	2007	199487.81 (183243.54to214508.45)
DALYs (Disability-Adjusted Life Years)	Asia	80 to 84	2007	111821.31 (98032.05to122497.8)
DALYs (Disability-Adjusted Life Years)	Asia	85 to 89	2007	51434.93 (43127.04to57589.04)

DALYs (Disability-Adjusted Life Years)	Asia	90 to 94	2007	19799.53 (16044.66to22296.94)
DALYs (Disability-Adjusted Life Years)	Asia	95 plus	2007	6246.29 (4634.19to7256.52)
DALYs (Disability-Adjusted Life Years)	Asia	<5	2008	0 (0to0)
DALYs (Disability-Adjusted Life Years)	Asia	5 to 9	2008	0 (0to0)
DALYs (Disability-Adjusted Life Years)	Asia	10 to 14	2008	0 (0to0)
DALYs (Disability-Adjusted Life Years)	Asia	15 to 19	2008	25522.25 (21735.38to30104.13)
DALYs (Disability-Adjusted Life Years)	Asia	20 to 24	2008	59220.45 (52905.25to66231.82)
DALYs (Disability-Adjusted Life Years)	Asia	25 to 29	2008	136685.81 (123734.76to151001.8)
DALYs (Disability-Adjusted Life Years)	Asia	30 to 34	2008	319572.17 (296439.84to346137.19)
DALYs (Disability-Adjusted Life Years)	Asia	35 to 39	2008	610436.72 (569677.56to654645.7)
DALYs (Disability-Adjusted Life Years)	Asia	40 to 44	2008	928856.38 (858988.19to1006354.41)

DALYs (Disability-Adjusted Life Years)	Asia	45 to 49	2008	974811.07 (921206.09to1032606.2)
DALYs (Disability-Adjusted Life Years)	Asia	50 to 54	2008	1071418.67 (1003459.96to1149293.52)
DALYs (Disability-Adjusted Life Years)	Asia	55 to 59	2008	965546.26 (905796.08to1034164.81)
DALYs (Disability-Adjusted Life Years)	Asia	60 to 64	2008	628276.79 (587606.23to675459.11)
DALYs (Disability-Adjusted Life Years)	Asia	65 to 69	2008	460124.23 (427173.26to490680.31)
DALYs (Disability-Adjusted Life Years)	Asia	70 to 74	2008	347575.97 (321436.61to373743.2)
DALYs (Disability-Adjusted Life Years)	Asia	75 to 79	2008	210157.04 (192493.35to226288.38)
DALYs (Disability-Adjusted Life Years)	Asia	80 to 84	2008	118167.43 (103071.08to128094.46)
DALYs (Disability-Adjusted Life Years)	Asia	85 to 89	2008	54617.84 (45766.5to60435.17)
DALYs (Disability-Adjusted Life Years)	Asia	90 to 94	2008	20636.6 (16492.28to23179.84)

DALYs (Disability-Adjusted Life Years)	Asia	95 plus	2008	6661.41 (4940.77to7715.1)
DALYs (Disability-Adjusted Life Years)	Asia	<5	2009	0 (0to0)
DALYs (Disability-Adjusted Life Years)	Asia	5 to 9	2009	0 (0to0)
DALYs (Disability-Adjusted Life Years)	Asia	10 to 14	2009	0 (0to0)
DALYs (Disability-Adjusted Life Years)	Asia	15 to 19	2009	25915.44 (21921.92to30539.7)
DALYs (Disability-Adjusted Life Years)	Asia	20 to 24	2009	61003.39 (54711.37to67868.44)
DALYs (Disability-Adjusted Life Years)	Asia	25 to 29	2009	145003.75 (129312.27to161060.15)
DALYs (Disability-Adjusted Life Years)	Asia	30 to 34	2009	325968.71 (303220.52to352902.5)
DALYs (Disability-Adjusted Life Years)	Asia	35 to 39	2009	611837.16 (572333.55to659443.11)
DALYs (Disability-Adjusted Life Years)	Asia	40 to 44	2009	922901.91 (855977.93to990816.73)
DALYs (Disability-Adjusted Life Years)	Asia	45 to 49	2009	1040807.1 (978831.1to1111104.81)

DALYs (Disability-Adjusted Life Years)	Asia	50 to 54	2009	1068824.62 (997187.34to1138717.7)
DALYs (Disability-Adjusted Life Years)	Asia	55 to 59	2009	987022.56 (924631.79to1053039.83)
DALYs (Disability-Adjusted Life Years)	Asia	60 to 64	2009	670900.98 (627802.01to721755.65)
DALYs (Disability-Adjusted Life Years)	Asia	65 to 69	2009	479961.4 (446726.5to513430.14)
DALYs (Disability-Adjusted Life Years)	Asia	70 to 74	2009	356564.36 (332323.05to382552.25)
DALYs (Disability-Adjusted Life Years)	Asia	75 to 79	2009	220739.66 (202312.35to237839.19)
DALYs (Disability-Adjusted Life Years)	Asia	80 to 84	2009	123260.03 (107649.22to134223.82)
DALYs (Disability-Adjusted Life Years)	Asia	85 to 89	2009	57164.98 (47334.73to63443.67)
DALYs (Disability-Adjusted Life Years)	Asia	90 to 94	2009	21212.81 (16970.69to23631.88)
DALYs (Disability-Adjusted Life Years)	Asia	95 plus	2009	7094.03 (5194.27to8206.32)

DALYs (Disability-Adjusted Life Years)	Asia	<5	2010	0<br>(0to0)
DALYs (Disability-Adjusted Life Years)	Asia	5 to 9	2010	0<br>(0to0)
DALYs (Disability-Adjusted Life Years)	Asia	10 to 14	2010	0<br>(0to0)
DALYs (Disability-Adjusted Life Years)	Asia	15 to 19	2010	26341.26<br>(22090.87to30894.01)
DALYs (Disability-Adjusted Life Years)	Asia	20 to 24	2010	63135.12<br>(56277.7to69938.26)
DALYs (Disability-Adjusted Life Years)	Asia	25 to 29	2010	151321.64<br>(135681.58to167432.31)
DALYs (Disability-Adjusted Life Years)	Asia	30 to 34	2010	332175.62<br>(306898.32to356768.69)
DALYs (Disability-Adjusted Life Years)	Asia	35 to 39	2010	606342.7<br>(569843.04to641815.91)
DALYs (Disability-Adjusted Life Years)	Asia	40 to 44	2010	926347.08<br>(871670.42to987523.89)
DALYs (Disability-Adjusted Life Years)	Asia	45 to 49	2010	1108518.89<br>(1042020.24to1182033.36)
DALYs (Disability-Adjusted Life Years)	Asia	50 to 54	2010	1082581.82<br>(1019322.67to1148723.36)

DALYs (Disability-Adjusted Life Years)	Asia	55 to 59	2010	1037641.19 (973997.28to1100724.13)
DALYs (Disability-Adjusted Life Years)	Asia	60 to 64	2010	712226.1 (668199.61to763205.55)
DALYs (Disability-Adjusted Life Years)	Asia	65 to 69	2010	500248.42 (466849.42to532053.79)
DALYs (Disability-Adjusted Life Years)	Asia	70 to 74	2010	367390.62 (343511.24to393023.23)
DALYs (Disability-Adjusted Life Years)	Asia	75 to 79	2010	231733.94 (212630.15to248425.31)
DALYs (Disability-Adjusted Life Years)	Asia	80 to 84	2010	130062.05 (113680.46to141996.04)
DALYs (Disability-Adjusted Life Years)	Asia	85 to 89	2010	60914.46 (50225.44to68152.79)
DALYs (Disability-Adjusted Life Years)	Asia	90 to 94	2010	22894.18 (17980.65to25747.97)
DALYs (Disability-Adjusted Life Years)	Asia	95 plus	2010	7828.27 (5685.39to9068.86)
DALYs (Disability-Adjusted Life Years)	Asia	<5	2012	0 (0to0)
DALYs (Disability-Adjusted Life Years)	Asia	5 to 9	2012	0 (0to0)

DALYs (Disability-Adjusted Life Years)	Asia	10 to 14	2012	0 (0to0)
DALYs (Disability-Adjusted Life Years)	Asia	15 to 19	2012	27054.22 (22024.47to32725.95)
DALYs (Disability-Adjusted Life Years)	Asia	20 to 24	2012	66260.92 (59221.25to73548.26)
DALYs (Disability-Adjusted Life Years)	Asia	25 to 29	2012	160446.04 (145325.71to177247.03)
DALYs (Disability-Adjusted Life Years)	Asia	30 to 34	2012	339346.04 (311600.54to368508.25)
DALYs (Disability-Adjusted Life Years)	Asia	35 to 39	2012	607545.19 (569054.02to646075.68)
DALYs (Disability-Adjusted Life Years)	Asia	40 to 44	2012	947301.59 (886743.45to1018052.64)
DALYs (Disability-Adjusted Life Years)	Asia	45 to 49	2012	1156716.33 (1076767.6to1242613.7)
DALYs (Disability-Adjusted Life Years)	Asia	50 to 54	2012	1135531.7 (1058316.11to1218692.48)
DALYs (Disability-Adjusted Life Years)	Asia	55 to 59	2012	1093030.62 (1008589.76to1181906.79)

DALYs (Disability-Adjusted Life Years)	Asia	60 to 64	2012	801121.59 (745420.15to863370.62)
DALYs (Disability-Adjusted Life Years)	Asia	65 to 69	2012	541114.85 (499145.69to584217.26)
DALYs (Disability-Adjusted Life Years)	Asia	70 to 74	2012	385943.77 (356149.54to414722.36)
DALYs (Disability-Adjusted Life Years)	Asia	75 to 79	2012	248243.86 (225004.21to268643.53)
DALYs (Disability-Adjusted Life Years)	Asia	80 to 84	2012	145093.83 (126488.56to158210.27)
DALYs (Disability-Adjusted Life Years)	Asia	85 to 89	2012	69265.46 (57072.97to76850.56)
DALYs (Disability-Adjusted Life Years)	Asia	90 to 94	2012	26767.6 (20946.92to30316.58)
DALYs (Disability-Adjusted Life Years)	Asia	95 plus	2012	9574.98 (6925.55to11097.75)
DALYs (Disability-Adjusted Life Years)	Asia	<5	2013	0 (0to0)
DALYs (Disability-Adjusted Life Years)	Asia	5 to 9	2013	0 (0to0)
DALYs (Disability-Adjusted Life Years)	Asia	10 to 14	2013	0 (0to0)

DALYs (Disability-Adjusted Life Years)	Asia	15 to 19	2013	27757.74 (22430.94to33739.23)
DALYs (Disability-Adjusted Life Years)	Asia	20 to 24	2013	67370.1 (58903.96to77344.9)
DALYs (Disability-Adjusted Life Years)	Asia	25 to 29	2013	165884.38 (149232.02to186457.06)
DALYs (Disability-Adjusted Life Years)	Asia	30 to 34	2013	348542.26 (320125.92to382558.76)
DALYs (Disability-Adjusted Life Years)	Asia	35 to 39	2013	620280.54 (579328.5to666217.11)
DALYs (Disability-Adjusted Life Years)	Asia	40 to 44	2013	971623.63 (902890.41to1048772.56)
DALYs (Disability-Adjusted Life Years)	Asia	45 to 49	2013	1189350.21 (1103397.02to1285610.48)
DALYs (Disability-Adjusted Life Years)	Asia	50 to 54	2013	1197445.43 (1101586.92to1307843.72)
DALYs (Disability-Adjusted Life Years)	Asia	55 to 59	2013	1118076.13 (1038278.45to1202742.98)
DALYs (Disability-Adjusted Life Years)	Asia	60 to 64	2013	840651.44 (781934to904449.27)

DALYs (Disability-Adjusted Life Years)	Asia	65 to 69	2013	561877.15 (517224.79to607033.32)
DALYs (Disability-Adjusted Life Years)	Asia	70 to 74	2013	395155.78 (364245.13to423131.83)
DALYs (Disability-Adjusted Life Years)	Asia	75 to 79	2013	257017.01 (234924.81to275825.22)
DALYs (Disability-Adjusted Life Years)	Asia	80 to 84	2013	146592.88 (126912.27to163534.95)
DALYs (Disability-Adjusted Life Years)	Asia	85 to 89	2013	72296.07 (58370.97to81391.57)
DALYs (Disability-Adjusted Life Years)	Asia	90 to 94	2013	29211.61 (22614.32to33292.27)
DALYs (Disability-Adjusted Life Years)	Asia	95 plus	2013	10534.59 (7567.08to12212.03)
DALYs (Disability-Adjusted Life Years)	Asia	<5	2011	0 (0to0)
DALYs (Disability-Adjusted Life Years)	Asia	5 to 9	2011	0 (0to0)
DALYs (Disability-Adjusted Life Years)	Asia	10 to 14	2011	0 (0to0)
DALYs (Disability-Adjusted Life Years)	Asia	15 to 19	2011	27019.04 (22797.07to32163.5)

DALYs (Disability-Adjusted Life Years)	Asia	20 to 24	2011	65135.58 (57596.88to72380.19)
DALYs (Disability-Adjusted Life Years)	Asia	25 to 29	2011	155909.4 (140170.33to171771.36)
DALYs (Disability-Adjusted Life Years)	Asia	30 to 34	2011	336772.89 (309773.37to365081.98)
DALYs (Disability-Adjusted Life Years)	Asia	35 to 39	2011	601289.47 (562755.2to645104.5)
DALYs (Disability-Adjusted Life Years)	Asia	40 to 44	2011	938974.95 (877472.09to996158.41)
DALYs (Disability-Adjusted Life Years)	Asia	45 to 49	2011	1145018.19 (1068915.67to1226023.86)
DALYs (Disability-Adjusted Life Years)	Asia	50 to 54	2011	1102316.47 (1030850.34to1174117.5)
DALYs (Disability-Adjusted Life Years)	Asia	55 to 59	2011	1066808.5 (991098.39to1140353.36)
DALYs (Disability-Adjusted Life Years)	Asia	60 to 64	2011	766343.17 (714517.49to815846.06)
DALYs (Disability-Adjusted Life Years)	Asia	65 to 69	2011	519219.83 (483528.78to556433.51)

DALYs (Disability-Adjusted Life Years)	Asia	70 to 74	2011	377606.81 (351396.86to404658.29)
DALYs (Disability-Adjusted Life Years)	Asia	75 to 79	2011	241879.86 (220551.45to260611.97)
DALYs (Disability-Adjusted Life Years)	Asia	80 to 84	2011	139962.61 (122114.66to153177.65)
DALYs (Disability-Adjusted Life Years)	Asia	85 to 89	2011	65668.05 (54557.07to73512.37)
DALYs (Disability-Adjusted Life Years)	Asia	90 to 94	2011	24894.45 (19744.23to28055.3)
DALYs (Disability-Adjusted Life Years)	Asia	95 plus	2011	8801.57 (6409.64to10246.89)
DALYs (Disability-Adjusted Life Years)	Asia	<5	2014	0 (0to0)
DALYs (Disability-Adjusted Life Years)	Asia	5 to 9	2014	0 (0to0)
DALYs (Disability-Adjusted Life Years)	Asia	10 to 14	2014	0 (0to0)
DALYs (Disability-Adjusted Life Years)	Asia	15 to 19	2014	28073.63 (22260.43to34558.75)
DALYs (Disability-Adjusted Life Years)	Asia	20 to 24	2014	67256.46 (55502.3to79208.45)

DALYs (Disability-Adjusted Life Years)	Asia	25 to 29	2014	165854.21 (149303.05to186418.63)
DALYs (Disability-Adjusted Life Years)	Asia	30 to 34	2014	355400.99 (326141.76to386143.9)
DALYs (Disability-Adjusted Life Years)	Asia	35 to 39	2014	624195.95 (582981.84to667111.94)
DALYs (Disability-Adjusted Life Years)	Asia	40 to 44	2014	995495.2 (921914.96to1072583.31)
DALYs (Disability-Adjusted Life Years)	Asia	45 to 49	2014	1205443.71 (1116760.56to1307645.04)
DALYs (Disability-Adjusted Life Years)	Asia	50 to 54	2014	1274697.5 (1151344.11to1418453.93)
DALYs (Disability-Adjusted Life Years)	Asia	55 to 59	2014	1151001.25 (1068990.48to1239408.95)
DALYs (Disability-Adjusted Life Years)	Asia	60 to 64	2014	867820.71 (804575.99to936162.53)
DALYs (Disability-Adjusted Life Years)	Asia	65 to 69	2014	586230.04 (542677.34to630223.99)
DALYs (Disability-Adjusted Life Years)	Asia	70 to 74	2014	403501.07 (375295.04to432545.27)

DALYs (Disability-Adjusted Life Years)	Asia	75 to 79	2014	264285.11 (240928.01to285496.96)
DALYs (Disability-Adjusted Life Years)	Asia	80 to 84	2014	151878.22 (128965.81to171700.6)
DALYs (Disability-Adjusted Life Years)	Asia	85 to 89	2014	75914.05 (61375.51to87619.45)
DALYs (Disability-Adjusted Life Years)	Asia	90 to 94	2014	31556.36 (24482.5to36503.61)
DALYs (Disability-Adjusted Life Years)	Asia	95 plus	2014	11145.38 (8039.03to12919.56)
DALYs (Disability-Adjusted Life Years)	Asia	<5	2021	0 (0to0)
DALYs (Disability-Adjusted Life Years)	Asia	5 to 9	2021	0 (0to0)
DALYs (Disability-Adjusted Life Years)	Asia	10 to 14	2021	0 (0to0)
DALYs (Disability-Adjusted Life Years)	Asia	15 to 19	2021	31309.57 (23109.76to40674.29)
DALYs (Disability-Adjusted Life Years)	Asia	20 to 24	2021	75171.58 (59814.01to95965.82)
DALYs (Disability-Adjusted Life Years)	Asia	25 to 29	2021	177813.63 (151141.63to209739.95)

DALYs (Disability-Adjusted Life Years)	Asia	30 to 34	2021	435467.29 (383937.8to492446.18)
DALYs (Disability-Adjusted Life Years)	Asia	35 to 39	2021	736085.91 (660469.19to816788.33)
DALYs (Disability-Adjusted Life Years)	Asia	40 to 44	2021	1073061.49 (951997.49to1202058.58)
DALYs (Disability-Adjusted Life Years)	Asia	45 to 49	2021	1383361.21 (1233945.44to1541469.91)
DALYs (Disability-Adjusted Life Years)	Asia	50 to 54	2021	1620795.89 (1436581.53to1831453.24)
DALYs (Disability-Adjusted Life Years)	Asia	55 to 59	2021	1569131.52 (1356769.19to1806675.98)
DALYs (Disability-Adjusted Life Years)	Asia	60 to 64	2021	1051185.6 (935903.72to1175629.73)
DALYs (Disability-Adjusted Life Years)	Asia	65 to 69	2021	874030.13 (787329.11to978178.8)
DALYs (Disability-Adjusted Life Years)	Asia	70 to 74	2021	593932.63 (528895.08to660999.72)
DALYs (Disability-Adjusted Life Years)	Asia	75 to 79	2021	332155.57 (290264.78to372643.5)

DALYs (Disability-Adjusted Life Years)	Asia	80 to 84	2021	202881.12 (171337.98to230439.84)
DALYs (Disability-Adjusted Life Years)	Asia	85 to 89	2021	108620.87 (85314.69to124756.62)
DALYs (Disability-Adjusted Life Years)	Asia	90 to 94	2021	50187.12 (37760.64to58195.67)
DALYs (Disability-Adjusted Life Years)	Asia	95 plus	2021	19985.13 (14027.61to23663.72)
DALYs (Disability-Adjusted Life Years)	Asia	<5	2016	0 (0to0)
DALYs (Disability-Adjusted Life Years)	Asia	5 to 9	2016	0 (0to0)
DALYs (Disability-Adjusted Life Years)	Asia	10 to 14	2016	0 (0to0)
DALYs (Disability-Adjusted Life Years)	Asia	15 to 19	2016	28379.45 (22073.95to36306.01)
DALYs (Disability-Adjusted Life Years)	Asia	20 to 24	2016	69121.14 (55309.47to84912.43)
DALYs (Disability-Adjusted Life Years)	Asia	25 to 29	2016	171374.99 (149916.89to195602.76)
DALYs (Disability-Adjusted Life Years)	Asia	30 to 34	2016	379466.33 (345727.25to418715.25)

DALYs (Disability-Adjusted Life Years)	Asia	35 to 39	2016	654959.4 (608440.71to705600.94)
DALYs (Disability-Adjusted Life Years)	Asia	40 to 44	2016	1030240.25 (956162.38to1119986.31)
DALYs (Disability-Adjusted Life Years)	Asia	45 to 49	2016	1257109.67 (1150999.23to1372685.93)
DALYs (Disability-Adjusted Life Years)	Asia	50 to 54	2016	1426951.16 (1267219.98to1615858.1)
DALYs (Disability-Adjusted Life Years)	Asia	55 to 59	2016	1222054.71 (1120357.06to1334907.74)
DALYs (Disability-Adjusted Life Years)	Asia	60 to 64	2016	951583.81 (876184.46to1034034.06)
DALYs (Disability-Adjusted Life Years)	Asia	65 to 69	2016	668569.81 (613338to731927.76)
DALYs (Disability-Adjusted Life Years)	Asia	70 to 74	2016	435821.54 (402741.43to472400.03)
DALYs (Disability-Adjusted Life Years)	Asia	75 to 79	2016	279659.52 (252306.32to303657.91)
DALYs (Disability-Adjusted Life Years)	Asia	80 to 84	2016	171204.25 (147796.8to190020.63)

DALYs (Disability-Adjusted Life Years)	Asia	85 to 89	2016	87226.52 (70521.61to97810.39)
DALYs (Disability-Adjusted Life Years)	Asia	90 to 94	2016	37270.41 (28988.1to42688.78)
DALYs (Disability-Adjusted Life Years)	Asia	95 plus	2016	13499.05 (9562.81to15692.28)
DALYs (Disability-Adjusted Life Years)	Asia	<5	2017	0 (0to0)
DALYs (Disability-Adjusted Life Years)	Asia	5 to 9	2017	0 (0to0)
DALYs (Disability-Adjusted Life Years)	Asia	10 to 14	2017	0 (0to0)
DALYs (Disability-Adjusted Life Years)	Asia	15 to 19	2017	28986.46 (22376.57to36671.8)
DALYs (Disability-Adjusted Life Years)	Asia	20 to 24	2017	70166.07 (56194.54to85056.8)
DALYs (Disability-Adjusted Life Years)	Asia	25 to 29	2017	175802.63 (152504.45to201543.6)
DALYs (Disability-Adjusted Life Years)	Asia	30 to 34	2017	397755.05 (360473.75to438672.03)
DALYs (Disability-Adjusted Life Years)	Asia	35 to 39	2017	673724.69 (623211.32to726072.6)

DALYs (Disability-Adjusted Life Years)	Asia	40 to 44	2017	1040086.74 (943168.21to1137425.07)
DALYs (Disability-Adjusted Life Years)	Asia	45 to 49	2017	1299325 (1173941.93to1424523.74)
DALYs (Disability-Adjusted Life Years)	Asia	50 to 54	2017	1496818.3 (1337100.37to1680373.72)
DALYs (Disability-Adjusted Life Years)	Asia	55 to 59	2017	1269933.2 (1146097.3to1404273.47)
DALYs (Disability-Adjusted Life Years)	Asia	60 to 64	2017	992368.48 (898833.06to1092031.54)
DALYs (Disability-Adjusted Life Years)	Asia	65 to 69	2017	722839.02 (657128.66to788714.2)
DALYs (Disability-Adjusted Life Years)	Asia	70 to 74	2017	465633.24 (426749.76to508205.39)
DALYs (Disability-Adjusted Life Years)	Asia	75 to 79	2017	291523.34 (262793.13to318804.02)
DALYs (Disability-Adjusted Life Years)	Asia	80 to 84	2017	177515.39 (152702.61to200932.07)
DALYs (Disability-Adjusted Life Years)	Asia	85 to 89	2017	90235.51 (72446.43to102352.15)

DALYs (Disability-Adjusted Life Years)	Asia	90 to 94	2017	39688.14 (30594.44to45505.51)
DALYs (Disability-Adjusted Life Years)	Asia	95 plus	2017	14898.58 (10652.24to17420.98)
DALYs (Disability-Adjusted Life Years)	Asia	<5	2018	0 (0to0)
DALYs (Disability-Adjusted Life Years)	Asia	5 to 9	2018	0 (0to0)
DALYs (Disability-Adjusted Life Years)	Asia	10 to 14	2018	0 (0to0)
DALYs (Disability-Adjusted Life Years)	Asia	15 to 19	2018	28831.63 (21669.43to37916.27)
DALYs (Disability-Adjusted Life Years)	Asia	20 to 24	2018	70461.84 (55384.95to88475.29)
DALYs (Disability-Adjusted Life Years)	Asia	25 to 29	2018	174480.8 (148957.71to205798.16)
DALYs (Disability-Adjusted Life Years)	Asia	30 to 34	2018	406281.22 (361806.59to453070.99)
DALYs (Disability-Adjusted Life Years)	Asia	35 to 39	2018	680649.21 (630067.81to741687.75)
DALYs (Disability-Adjusted Life Years)	Asia	40 to 44	2018	1043044.56 (948841.88to1142445.05)

DALYs (Disability-Adjusted Life Years)	Asia	45 to 49	2018	1331509.23 (1206095.93to1462988.38)
DALYs (Disability-Adjusted Life Years)	Asia	50 to 54	2018	1544173.68 (1382209.9to1714688.63)
DALYs (Disability-Adjusted Life Years)	Asia	55 to 59	2018	1352012.9 (1205502.98to1510788.3)
DALYs (Disability-Adjusted Life Years)	Asia	60 to 64	2018	1024104.9 (927158.66to1118105.84)
DALYs (Disability-Adjusted Life Years)	Asia	65 to 69	2018	773985.39 (701732.04to850516.14)
DALYs (Disability-Adjusted Life Years)	Asia	70 to 74	2018	494753.84 (447476.61to537425.62)
DALYs (Disability-Adjusted Life Years)	Asia	75 to 79	2018	304405.28 (272058.27to333935.69)
DALYs (Disability-Adjusted Life Years)	Asia	80 to 84	2018	185451.64 (157700.18to209062.2)
DALYs (Disability-Adjusted Life Years)	Asia	85 to 89	2018	95119.48 (76363.09to107418.67)
DALYs (Disability-Adjusted Life Years)	Asia	90 to 94	2018	42552.93 (32355.13to49050.47)

DALYs (Disability-Adjusted Life Years)	Asia	95 plus	2018	16413.03 (11639.22to19302.99)
DALYs (Disability-Adjusted Life Years)	Asia	<5	2015	0 (0to0)
DALYs (Disability-Adjusted Life Years)	Asia	5 to 9	2015	0 (0to0)
DALYs (Disability-Adjusted Life Years)	Asia	10 to 14	2015	0 (0to0)
DALYs (Disability-Adjusted Life Years)	Asia	15 to 19	2015	28142.19 (21940.49to35342.23)
DALYs (Disability-Adjusted Life Years)	Asia	20 to 24	2015	67797.68 (54872.46to82412.74)
DALYs (Disability-Adjusted Life Years)	Asia	25 to 29	2015	166790.25 (147543.13to190952.36)
DALYs (Disability-Adjusted Life Years)	Asia	30 to 34	2015	364393.64 (331221.92to401477.21)
DALYs (Disability-Adjusted Life Years)	Asia	35 to 39	2015	635802.2 (586681.03to685952.4)
DALYs (Disability-Adjusted Life Years)	Asia	40 to 44	2015	1015310.42 (926928.62to1097882.07)
DALYs (Disability-Adjusted Life Years)	Asia	45 to 49	2015	1222087.29 (1114357.88to1323182.79)

DALYs (Disability-Adjusted Life Years)	Asia	50 to 54	2015	1360684.87 (1189581.61to1536503.25)
DALYs (Disability-Adjusted Life Years)	Asia	55 to 59	2015	1188781.85 (1081259.4to1294545.94)
DALYs (Disability-Adjusted Life Years)	Asia	60 to 64	2015	917446.09 (839480.07to996878.22)
DALYs (Disability-Adjusted Life Years)	Asia	65 to 69	2015	618626.1 (566030.98to670921.89)
DALYs (Disability-Adjusted Life Years)	Asia	70 to 74	2015	416253.47 (382233.31to447833.56)
DALYs (Disability-Adjusted Life Years)	Asia	75 to 79	2015	271481.52 (243824.31to296500.72)
DALYs (Disability-Adjusted Life Years)	Asia	80 to 84	2015	160861.75 (137862.02to180700.71)
DALYs (Disability-Adjusted Life Years)	Asia	85 to 89	2015	81508.45 (66034.83to92210.51)
DALYs (Disability-Adjusted Life Years)	Asia	90 to 94	2015	34006.68 (26243.32to38822.78)
DALYs (Disability-Adjusted Life Years)	Asia	95 plus	2015	12045.64 (8629.18to14068.5)

DALYs (Disability-Adjusted Life Years)	Asia	<5	2020	0 (0to0)
DALYs (Disability-Adjusted Life Years)	Asia	5 to 9	2020	0 (0to0)
DALYs (Disability-Adjusted Life Years)	Asia	10 to 14	2020	0 (0to0)
DALYs (Disability-Adjusted Life Years)	Asia	15 to 19	2020	30965.58 (22524.68to41477.88)
DALYs (Disability-Adjusted Life Years)	Asia	20 to 24	2020	74570.02 (57563.36to94518.13)
DALYs (Disability-Adjusted Life Years)	Asia	25 to 29	2020	177640.87 (151292.16to212172.11)
DALYs (Disability-Adjusted Life Years)	Asia	30 to 34	2020	432244.24 (374949.76to491930.8)
DALYs (Disability-Adjusted Life Years)	Asia	35 to 39	2020	718624.11 (648956.86to788757.07)
DALYs (Disability-Adjusted Life Years)	Asia	40 to 44	2020	1057801.06 (952824.84to1168829.67)
DALYs (Disability-Adjusted Life Years)	Asia	45 to 49	2020	1376600.34 (1236939.25to1524271.32)
DALYs (Disability-Adjusted Life Years)	Asia	50 to 54	2020	1604683.47 (1441738.51to1798170.54)

DALYs (Disability-Adjusted Life Years)	Asia	55 to 59	2020	1495672.91 (1299921.03to1723948.79)
DALYs (Disability-Adjusted Life Years)	Asia	60 to 64	2020	1039186.65 (933267.89to1153390.52)
DALYs (Disability-Adjusted Life Years)	Asia	65 to 69	2020	849701.93 (757877.55to945554.82)
DALYs (Disability-Adjusted Life Years)	Asia	70 to 74	2020	556832.76 (501560to617457.97)
DALYs (Disability-Adjusted Life Years)	Asia	75 to 79	2020	320430.27 (280444.27to360665.64)
DALYs (Disability-Adjusted Life Years)	Asia	80 to 84	2020	198437.92 (169889.55to221378.35)
DALYs (Disability-Adjusted Life Years)	Asia	85 to 89	2020	103606.16 (83615.5to117900.56)
DALYs (Disability-Adjusted Life Years)	Asia	90 to 94	2020	47488.11 (35733.09to54997.19)
DALYs (Disability-Adjusted Life Years)	Asia	95 plus	2020	18379.88 (12871.79to21637.64)
DALYs (Disability-Adjusted Life Years)	Asia	<5	2019	0 (0to0)
DALYs (Disability-Adjusted Life Years)	Asia	5 to 9	2019	0 (0to0)

DALYs (Disability-Adjusted Life Years)	Asia	10 to 14	2019	0 (0to0)
DALYs (Disability-Adjusted Life Years)	Asia	15 to 19	2019	29291.32 (21850.43to39021.28)
DALYs (Disability-Adjusted Life Years)	Asia	20 to 24	2019	71642.18 (55640.17to91542.96)
DALYs (Disability-Adjusted Life Years)	Asia	25 to 29	2019	176697.46 (152013.28to208379.17)
DALYs (Disability-Adjusted Life Years)	Asia	30 to 34	2019	415984.51 (367780.65to468897.19)
DALYs (Disability-Adjusted Life Years)	Asia	35 to 39	2019	695500.33 (636988.1to762268.96)
DALYs (Disability-Adjusted Life Years)	Asia	40 to 44	2019	1043804.93 (949059.61to1149254.71)
DALYs (Disability-Adjusted Life Years)	Asia	45 to 49	2019	1360859.42 (1225620.42to1512544.58)
DALYs (Disability-Adjusted Life Years)	Asia	50 to 54	2019	1576742.52 (1406199.81to1754456.64)
DALYs (Disability-Adjusted Life Years)	Asia	55 to 59	2019	1439779.88 (1265840.39to1638762.84)

DALYs (Disability-Adjusted Life Years)	Asia	60 to 64	2019	1043707.74 (942677.96to1148119.25)
DALYs (Disability-Adjusted Life Years)	Asia	65 to 69	2019	812533.99 (729461.21to895289.53)
DALYs (Disability-Adjusted Life Years)	Asia	70 to 74	2019	530181.92 (484760.24to583018.36)
DALYs (Disability-Adjusted Life Years)	Asia	75 to 79	2019	317502.71 (278114.33to354040.17)
DALYs (Disability-Adjusted Life Years)	Asia	80 to 84	2019	194491.87 (167198.31to217634.86)
DALYs (Disability-Adjusted Life Years)	Asia	85 to 89	2019	101491.55 (81497.56to115437.45)
DALYs (Disability-Adjusted Life Years)	Asia	90 to 94	2019	46401.36 (35479.16to53379.89)
DALYs (Disability-Adjusted Life Years)	Asia	95 plus	2019	17765.04 (12588to20838.96)

Supplementary Table S3. Age groups and age-standardized deaths rate of breast cancer in Asia between 1990–2021.

Measure	Location	Age	Year	Deaths

Deaths	Asia	15 to 19	1980	167.43 (140.73to199.56)
Deaths	Asia	20 to 24	1980	360 (289.73to448.81)
Deaths	Asia	25 to 29	1980	1170.25 (934.31to1468.07)
Deaths	Asia	30 to 34	1980	2735.42 (2215.32to3377.38)
Deaths	Asia	35 to 39	1980	5052.87 (4154to6126.32)
Deaths	Asia	40 to 44	1980	8174.69 (6856.62to9775.19)
Deaths	Asia	45 to 49	1980	10613.9 (9059.16to12550.98)
Deaths	Asia	50 to 54	1980	11986.09 (9895.86to14751.48)
Deaths	Asia	55 to 59	1980	11642.6 (9760.22to13968.93)
Deaths	Asia	60 to 64	1980	8830.45 (7660.42to10369.24)
Deaths	Asia	65 to 69	1980	7491.1 (6627.8to8543.2)
Deaths	Asia	70 to 74	1980	5939.66 (5261.76to6769.44)

Deaths	Asia	75 to 79	1980	3966.23 (3463.31to4522.16)
Deaths	Asia	80 to 84	1980	2192.99 (1882.38to2524.35)
Deaths	Asia	85 to 89	1980	1119.65 (950.96to1292.27)
Deaths	Asia	90 to 94	1980	407.87 (331.54to483.4)
Deaths	Asia	95 plus	1980	128.66 (95.94to160.92)
Deaths	Asia	15 to 19	1981	178.32 (147.19to213.02)
Deaths	Asia	20 to 24	1981	369.55 (296.29to460.44)
Deaths	Asia	25 to 29	1981	1212.76 (956.67to1533.67)
Deaths	Asia	30 to 34	1981	2909.54 (2359.78to3607.52)
Deaths	Asia	35 to 39	1981	5167.76 (4323.99to6287.83)
Deaths	Asia	40 to 44	1981	8262.72 (6889.18to9941.41)
Deaths	Asia	45 to 49	1981	10874.16 (9291.03to12857.46)

Deaths	Asia	50 to 54	1981	12331.81 (10164.17to14925.79)
Deaths	Asia	55 to 59	1981	11992.72 (10068.94to14487.07)
Deaths	Asia	60 to 64	1981	9125.25 (7909.41to10567.49)
Deaths	Asia	65 to 69	1981	7670.78 (6801.38to8720.05)
Deaths	Asia	70 to 74	1981	6087.41 (5378.67to6905.01)
Deaths	Asia	75 to 79	1981	4148.22 (3660.69to4684.22)
Deaths	Asia	80 to 84	1981	2322.45 (2030.97to2656.62)
Deaths	Asia	85 to 89	1981	1144.13 (976.08to1323.12)
Deaths	Asia	90 to 94	1981	417.39 (341.91to495.31)
Deaths	Asia	95 plus	1981	130.58 (97.44to165.16)
Deaths	Asia	15 to 19	1983	191.39 (160.61to226.36)
Deaths	Asia	20 to 24	1983	397.21 (323.48to489.94)

Deaths	Asia	25 to 29	1983	1212.41 (986.6to1479.5)
Deaths	Asia	30 to 34	1983	3165.8 (2603.45to3864.9)
Deaths	Asia	35 to 39	1983	5468.96 (4638.95to6512.58)
Deaths	Asia	40 to 44	1983	8354.27 (7171.75to9870.52)
Deaths	Asia	45 to 49	1983	11141.66 (9617.84to13106.02)
Deaths	Asia	50 to 54	1983	12719.52 (10560.99to15303.38)
Deaths	Asia	55 to 59	1983	12494.12 (10619.74to14944.98)
Deaths	Asia	60 to 64	1983	9563.05 (8335.49to11102.18)
Deaths	Asia	65 to 69	1983	7942.07 (7038.87to9008.37)
Deaths	Asia	70 to 74	1983	6398.73 (5711.67to7220.29)
Deaths	Asia	75 to 79	1983	4497.14 (3998.7to5047.31)
Deaths	Asia	80 to 84	1983	2582.67 (2264.12to2907.72)

Deaths	Asia	85 to 89	1983	1266.62 (1094.16to1434.41)
Deaths	Asia	90 to 94	1983	442.75 (362.53to518.3)
Deaths	Asia	95 plus	1983	138.79 (104.4to172.55)
Deaths	Asia	15 to 19	1982	184.49 (152.85to220.77)
Deaths	Asia	20 to 24	1982	376.02 (304.27to460.23)
Deaths	Asia	25 to 29	1982	1205.74 (966.9to1489.79)
Deaths	Asia	30 to 34	1982	3002.36 (2441.23to3644.21)
Deaths	Asia	35 to 39	1982	5297.27 (4480.84to6326.03)
Deaths	Asia	40 to 44	1982	8262.25 (7013.17to9809.35)
Deaths	Asia	45 to 49	1982	10971.93 (9432.84to12840.24)
Deaths	Asia	50 to 54	1982	12497.51 (10535.33to15127.56)
Deaths	Asia	55 to 59	1982	12222.34 (10331.34to14636.81)

Deaths	Asia	60 to 64	1982	9311.1 (8085.9to10812.11)
Deaths	Asia	65 to 69	1982	7795.25 (6948.79to8884.43)
Deaths	Asia	70 to 74	1982	6223.17 (5605.14to7062.26)
Deaths	Asia	75 to 79	1982	4329.96 (3849.42to4911.41)
Deaths	Asia	80 to 84	1982	2448.12 (2150.2to2792.69)
Deaths	Asia	85 to 89	1982	1207.35 (1040.84to1372.63)
Deaths	Asia	90 to 94	1982	433.78 (355.04to512.8)
Deaths	Asia	95 plus	1982	133.01 (99.72to168.06)
Deaths	Asia	15 to 19	1984	195.88 (167.01to229.68)
Deaths	Asia	20 to 24	1984	420.24 (346.18to510.39)
Deaths	Asia	25 to 29	1984	1217.05 (1010.25to1472.09)
Deaths	Asia	30 to 34	1984	3277.71 (2761.21to3949.47)

Deaths	Asia	35 to 39	1984	5762.9 (4983.91to6703.69)
Deaths	Asia	40 to 44	1984	8519.14 (7476.61to9832.88)
Deaths	Asia	45 to 49	1984	11253.35 (9913.81to12926.38)
Deaths	Asia	50 to 54	1984	12949.27 (10981.08to15470.93)
Deaths	Asia	55 to 59	1984	12832.96 (11095.82to15070.84)
Deaths	Asia	60 to 64	1984	9833.37 (8657.22to11271.65)
Deaths	Asia	65 to 69	1984	8079.24 (7201.63to9103.81)
Deaths	Asia	70 to 74	1984	6582.66 (5895.22to7338.88)
Deaths	Asia	75 to 79	1984	4645.72 (4137.45to5161.58)
Deaths	Asia	80 to 84	1984	2716.58 (2412.17to3044.13)
Deaths	Asia	85 to 89	1984	1294.96 (1104.23to1457.46)
Deaths	Asia	90 to 94	1984	455.46 (375.3to530.78)

Deaths	Asia	95 plus	1984	141.59 (106.68to175.05)
Deaths	Asia	15 to 19	1985	201.09 (171.61to235.94)
Deaths	Asia	20 to 24	1985	448.64 (372.9to534.65)
Deaths	Asia	25 to 29	1985	1228.15 (1029.24to1461.65)
Deaths	Asia	30 to 34	1985	3472.35 (2919.15to4151.74)
Deaths	Asia	35 to 39	1985	6031.33 (5217.98to6946.75)
Deaths	Asia	40 to 44	1985	8699.3 (7601.25to9995.51)
Deaths	Asia	45 to 49	1985	11449.02 (10036.18to13025.89)
Deaths	Asia	50 to 54	1985	13267.97 (11225.07to15649.46)
Deaths	Asia	55 to 59	1985	13236.24 (11446.44to15332.27)
Deaths	Asia	60 to 64	1985	10172.5 (8890.07to11601.7)
Deaths	Asia	65 to 69	1985	8329.01 (7324.79to9414.86)

Deaths	Asia	70 to 74	1985	6797.61 (6048.82to7583.42)
Deaths	Asia	75 to 79	1985	4818.41 (4280.01to5402)
Deaths	Asia	80 to 84	1985	2861.02 (2529.09to3186.06)
Deaths	Asia	85 to 89	1985	1342.72 (1160.43to1518.03)
Deaths	Asia	90 to 94	1985	477.52 (396.73to552.93)
Deaths	Asia	95 plus	1985	146.48 (111.2to178.07)
Deaths	Asia	15 to 19	1986	206.02 (177.94to238.07)
Deaths	Asia	20 to 24	1986	477.09 (400.54to569.93)
Deaths	Asia	25 to 29	1986	1249.84 (1047.69to1484.71)
Deaths	Asia	30 to 34	1986	3579.3 (2981.79to4250.26)
Deaths	Asia	35 to 39	1986	6388.9 (5525.62to7347.45)
Deaths	Asia	40 to 44	1986	8953.37 (7846.01to10171.17)

Deaths	Asia	45 to 49	1986	11570.39 (10248.57to13203.57)
Deaths	Asia	50 to 54	1986	13632.66 (11741.27to16084)
Deaths	Asia	55 to 59	1986	13610.13 (11893to15855.84)
Deaths	Asia	60 to 64	1986	10509.47 (9314.48to11849.16)
Deaths	Asia	65 to 69	1986	8608.35 (7808.22to9664.24)
Deaths	Asia	70 to 74	1986	6985.75 (6353.9to7799.82)
Deaths	Asia	75 to 79	1986	4976.8 (4488.57to5575.05)
Deaths	Asia	80 to 84	1986	3008.16 (2704.52to3341.46)
Deaths	Asia	85 to 89	1986	1414.09 (1218.33to1591.44)
Deaths	Asia	90 to 94	1986	499.39 (417.46to568.78)
Deaths	Asia	95 plus	1986	149.61 (114.17to178.87)
Deaths	Asia	15 to 19	1987	211.39 (182.32to243.89)

Deaths	Asia	20 to 24	1987	507.27 (426.43to609.82)
Deaths	Asia	25 to 29	1987	1302.15 (1095.36to1574.87)
Deaths	Asia	30 to 34	1987	3695.2 (3128.57to4368.77)
Deaths	Asia	35 to 39	1987	6773 (5967.59to7769.81)
Deaths	Asia	40 to 44	1987	9355.11 (8276.59to10641.95)
Deaths	Asia	45 to 49	1987	11739.74 (10464.62to13275.16)
Deaths	Asia	50 to 54	1987	14039.94 (12017.4to16416.4)
Deaths	Asia	55 to 59	1987	14069.56 (12257.85to16302.57)
Deaths	Asia	60 to 64	1987	10932.57 (9677.22to12451.79)
Deaths	Asia	65 to 69	1987	8948.76 (8006.31to10069.33)
Deaths	Asia	70 to 74	1987	7212.1 (6479.27to8063.37)
Deaths	Asia	75 to 79	1987	5162.3 (4626.06to5792.79)

Deaths	Asia	80 to 84	1987	3190.06 (2829.86to3537)
Deaths	Asia	85 to 89	1987	1507.19 (1284.65to1688.39)
Deaths	Asia	90 to 94	1987	518.97 (431.54to592.42)
Deaths	Asia	95 plus	1987	151.97 (117.63to179.74)
Deaths	Asia	15 to 19	1988	217.92 (187.65to249.52)
Deaths	Asia	20 to 24	1988	532.51 (449.29to628.61)
Deaths	Asia	25 to 29	1988	1380.02 (1174.49to1632.75)
Deaths	Asia	30 to 34	1988	3785.91 (3243.4to4495.45)
Deaths	Asia	35 to 39	1988	7229.47 (6339.3to8304.96)
Deaths	Asia	40 to 44	1988	9770.91 (8649.69to11123.44)
Deaths	Asia	45 to 49	1988	11971.16 (10689.86to13477.62)
Deaths	Asia	50 to 54	1988	14387.48 (12378.86to16945.01)

Deaths	Asia	55 to 59	1988	14514.05 (12776.76to16835.51)
Deaths	Asia	60 to 64	1988	11338.13 (10133.81to12984.69)
Deaths	Asia	65 to 69	1988	9332.82 (8421.79to10505.62)
Deaths	Asia	70 to 74	1988	7460.48 (6673.23to8393.5)
Deaths	Asia	75 to 79	1988	5394.87 (4816.02to6085.9)
Deaths	Asia	80 to 84	1988	3363.44 (2994.61to3752.16)
Deaths	Asia	85 to 89	1988	1648.41 (1419.11to1861.56)
Deaths	Asia	90 to 94	1988	555.26 (461.5to628.66)
Deaths	Asia	95 plus	1988	160.06 (124.75to189.29)
Deaths	Asia	15 to 19	1990	228.11 (197.42to265.63)
Deaths	Asia	20 to 24	1990	565.19 (485.03to666.8)
Deaths	Asia	25 to 29	1990	1527.45 (1303.67to1823.2)

Deaths	Asia	30 to 34	1990	3834.76 (3317.14to4476.45)
Deaths	Asia	35 to 39	1990	7928.52 (7022.53to9039.75)
Deaths	Asia	40 to 44	1990	10764.73 (9580.39to12203.86)
Deaths	Asia	45 to 49	1990	12511.52 (11370.22to13950.34)
Deaths	Asia	50 to 54	1990	14823.25 (12888.73to17099.98)
Deaths	Asia	55 to 59	1990	15296.8 (13369.29to17528.13)
Deaths	Asia	60 to 64	1990	12130.54 (10777.52to13748.46)
Deaths	Asia	65 to 69	1990	10106.08 (9014.09to11318.4)
Deaths	Asia	70 to 74	1990	7949.51 (7072.08to8840.15)
Deaths	Asia	75 to 79	1990	5810.91 (5167.95to6488.62)
Deaths	Asia	80 to 84	1990	3681.66 (3232.24to4067.86)
Deaths	Asia	85 to 89	1990	1917.95 (1637.12to2147.24)

Deaths	Asia	90 to 94	1990	627.29 (520.06to712.49)
Deaths	Asia	95 plus	1990	172.63 (134.11to203.16)
Deaths	Asia	15 to 19	1989	223.79 (193.28to258.35)
Deaths	Asia	20 to 24	1989	553.81 (464.83to663.75)
Deaths	Asia	25 to 29	1989	1463.62 (1239.08to1730.96)
Deaths	Asia	30 to 34	1989	3840.34 (3261.41to4539.77)
Deaths	Asia	35 to 39	1989	7570.47 (6636.13to8618.45)
Deaths	Asia	40 to 44	1989	10389.63 (9162.01to11801.11)
Deaths	Asia	45 to 49	1989	12271.17 (10923.12to13855.21)
Deaths	Asia	50 to 54	1989	14678.38 (12596.66to17051.66)
Deaths	Asia	55 to 59	1989	14986.79 (12991.75to17421.73)
Deaths	Asia	60 to 64	1989	11811.5 (10400.8to13389.72)

Deaths	Asia	65 to 69	1989	9794.17 (8729.88to10965.99)
Deaths	Asia	70 to 74	1989	7710.09 (6838.93to8628.21)
Deaths	Asia	75 to 79	1989	5632.72 (5002.21to6286.82)
Deaths	Asia	80 to 84	1989	3530.63 (3160.51to3865.35)
Deaths	Asia	85 to 89	1989	1800.52 (1567.4to2016.6)
Deaths	Asia	90 to 94	1989	589.81 (489.36to670.71)
Deaths	Asia	95 plus	1989	166.88 (130.65to196.4)
Deaths	Asia	15 to 19	1991	231.88 (201.98to262.48)
Deaths	Asia	20 to 24	1991	572.86 (492.1to672.65)
Deaths	Asia	25 to 29	1991	1597.09 (1370.52to1894)
Deaths	Asia	30 to 34	1991	3873.84 (3423.26to4501.91)
Deaths	Asia	35 to 39	1991	8134.43 (7327.85to9173.4)

Deaths	Asia	40 to 44	1991	11341.81 (10226.16to12691.39)
Deaths	Asia	45 to 49	1991	12896.8 (11792.14to14180.63)
Deaths	Asia	50 to 54	1991	15060.66 (13334.48to17251.65)
Deaths	Asia	55 to 59	1991	15783.58 (14134.76to18226.04)
Deaths	Asia	60 to 64	1991	12573.68 (11413.26to14188.89)
Deaths	Asia	65 to 69	1991	10472.18 (9518.47to11693.5)
Deaths	Asia	70 to 74	1991	8247.7 (7481.1to9198.53)
Deaths	Asia	75 to 79	1991	5997.49 (5408.08to6666.13)
Deaths	Asia	80 to 84	1991	3836.6 (3373.04to4214.13)
Deaths	Asia	85 to 89	1991	2030.38 (1730.34to2261.46)
Deaths	Asia	90 to 94	1991	675.92 (561.62to762.02)
Deaths	Asia	95 plus	1991	177.84 (139.44to209.21)

Deaths	Asia	15 to 19	1992	236.15 (205.31to272.83)
Deaths	Asia	20 to 24	1992	583.77 (499.1to698.24)
Deaths	Asia	25 to 29	1992	1648.77 (1403.54to1975.66)
Deaths	Asia	30 to 34	1992	3967.65 (3485.52to4643.95)
Deaths	Asia	35 to 39	1992	8303.98 (7443.2to9362.47)
Deaths	Asia	40 to 44	1992	11906.95 (10851.8to13348.31)
Deaths	Asia	45 to 49	1992	13395.86 (12237.93to14804.27)
Deaths	Asia	50 to 54	1992	15468.4 (13803.78to17610.76)
Deaths	Asia	55 to 59	1992	16314.52 (14582.85to18669.75)
Deaths	Asia	60 to 64	1992	13030.95 (11740.63to14719.72)
Deaths	Asia	65 to 69	1992	10872.18 (9819.19to12142.15)
Deaths	Asia	70 to 74	1992	8607.57 (7728.99to9607.01)

Deaths	Asia	75 to 79	1992	6261.36 (5612.57to6938.68)
Deaths	Asia	80 to 84	1992	4089.2 (3633.27to4458.02)
Deaths	Asia	85 to 89	1992	2189.71 (1880.12to2450.48)
Deaths	Asia	90 to 94	1992	742.37 (615.89to835.01)
Deaths	Asia	95 plus	1992	194.41 (151.77to227.41)
Deaths	Asia	15 to 19	1994	249.42 (217.3to286.71)
Deaths	Asia	20 to 24	1994	611.64 (519.62to721.5)
Deaths	Asia	25 to 29	1994	1729.97 (1473.07to2059.05)
Deaths	Asia	30 to 34	1994	4319.8 (3753.41to5056.51)
Deaths	Asia	35 to 39	1994	8431.16 (7606.89to9479.39)
Deaths	Asia	40 to 44	1994	13018.07 (11763.85to14534.57)
Deaths	Asia	45 to 49	1994	14544.24 (13264.78to16103.74)

Deaths	Asia	50 to 54	1994	16214.89 (14515to18191.67)
Deaths	Asia	55 to 59	1994	17315.3 (15659.4to19133.46)
Deaths	Asia	60 to 64	1994	14001.26 (12641.31to15697.01)
Deaths	Asia	65 to 69	1994	12006.66 (11030.83to13179.13)
Deaths	Asia	70 to 74	1994	9641.49 (8814.73to10576.08)
Deaths	Asia	75 to 79	1994	6924.75 (6290.63to7550.93)
Deaths	Asia	80 to 84	1994	4664.56 (4100.71to5130.52)
Deaths	Asia	85 to 89	1994	2533.77 (2148.74to2860.31)
Deaths	Asia	90 to 94	1994	918.73 (741.32to1048.76)
Deaths	Asia	95 plus	1994	237.61 (181.47to283.07)
Deaths	Asia	15 to 19	1995	254.25 (219.32to294.57)
Deaths	Asia	20 to 24	1995	624.7 (523.59to742.82)

Deaths	Asia	25 to 29	1995	1767.51 (1507.94to2107.42)
Deaths	Asia	30 to 34	1995	4565.54 (3935.87to5352.25)
Deaths	Asia	35 to 39	1995	8447.02 (7584.08to9334.05)
Deaths	Asia	40 to 44	1995	13733 (12477.7to15263.8)
Deaths	Asia	45 to 49	1995	15248.86 (13892.37to16799.43)
Deaths	Asia	50 to 54	1995	16738.44 (15038.94to18782.66)
Deaths	Asia	55 to 59	1995	17634.76 (16018.31to19443.24)
Deaths	Asia	60 to 64	1995	14453.72 (13004.48to16110.49)
Deaths	Asia	65 to 69	1995	12433.66 (11425.12to13555.28)
Deaths	Asia	70 to 74	1995	10057.89 (9233.34to11036.27)
Deaths	Asia	75 to 79	1995	7293.71 (6634.91to7988.69)
Deaths	Asia	80 to 84	1995	5021.44 (4367.64to5556.9)

Deaths	Asia	85 to 89	1995	2778.61 (2372.1to3140.78)
Deaths	Asia	90 to 94	1995	1048.67 (842.21to1209.27)
Deaths	Asia	95 plus	1995	267.38 (203.75to319.45)
Deaths	Asia	15 to 19	1997	270.56 (232.94to308.63)
Deaths	Asia	20 to 24	1997	655.6 (558.86to774.74)
Deaths	Asia	25 to 29	1997	1827.84 (1549.2to2174.41)
Deaths	Asia	30 to 34	1997	4928.72 (4302.3to5761.52)
Deaths	Asia	35 to 39	1997	8896.28 (8259.7to9638.9)
Deaths	Asia	40 to 44	1997	14412.74 (13250.77to15719.38)
Deaths	Asia	45 to 49	1997	16867.6 (15509.58to18370.11)
Deaths	Asia	50 to 54	1997	17814.52 (16104.31to19793.25)
Deaths	Asia	55 to 59	1997	18172.93 (16658.3to19863.74)

Deaths	Asia	60 to 64	1997	15427.51 (14051.82to17020.84)
Deaths	Asia	65 to 69	1997	13366.53 (12289.71to14652.11)
Deaths	Asia	70 to 74	1997	10955.97 (10122.01to11921.35)
Deaths	Asia	75 to 79	1997	7910.03 (7244.19to8624.62)
Deaths	Asia	80 to 84	1997	5321.13 (4661.56to5889.22)
Deaths	Asia	85 to 89	1997	2963.14 (2469.54to3342.55)
Deaths	Asia	90 to 94	1997	1159.26 (926.63to1332.63)
Deaths	Asia	95 plus	1997	303.1 (228.49to358.43)
Deaths	Asia	15 to 19	1998	279.81 (243.35to318.65)
Deaths	Asia	20 to 24	1998	674.15 (579.6to785.06)
Deaths	Asia	25 to 29	1998	1888.8 (1621.23to2195.81)
Deaths	Asia	30 to 34	1998	5062.65 (4416.68to5876.9)

Deaths	Asia	35 to 39	1998	9256.42 (8530.75to10120.48)
Deaths	Asia	40 to 44	1998	14538.92 (13307.97to15812.47)
Deaths	Asia	45 to 49	1998	17586.36 (16153.86to19202.66)
Deaths	Asia	50 to 54	1998	18494.71 (16766.66to20383.46)
Deaths	Asia	55 to 59	1998	18565.52 (17028.55to20210.29)
Deaths	Asia	60 to 64	1998	15864.73 (14462.19to17411.82)
Deaths	Asia	65 to 69	1998	13927.66 (12685.4to15278.21)
Deaths	Asia	70 to 74	1998	11462.15 (10507.81to12398.34)
Deaths	Asia	75 to 79	1998	8247.21 (7553.39to8944.03)
Deaths	Asia	80 to 84	1998	5508.13 (4810.31to6054.54)
Deaths	Asia	85 to 89	1998	3114.03 (2581.3to3475.46)
Deaths	Asia	90 to 94	1998	1243.77 (998.47to1436.06)

Deaths	Asia	95 plus	1998	334.76 (251.15to395.12)
Deaths	Asia	15 to 19	1999	293.93 (258.08to335.55)
Deaths	Asia	20 to 24	1999	694.27 (589.79to811.94)
Deaths	Asia	25 to 29	1999	1930.42 (1622.04to2280.17)
Deaths	Asia	30 to 34	1999	5217.26 (4514.26to6053.92)
Deaths	Asia	35 to 39	1999	9793.9 (8982.29to10679.25)
Deaths	Asia	40 to 44	1999	14695.65 (13386.07to16048.86)
Deaths	Asia	45 to 49	1999	18073.82 (16377.92to19870.76)
Deaths	Asia	50 to 54	1999	19512.14 (17729.6to21582.9)
Deaths	Asia	55 to 59	1999	19198.24 (17603.29to20838.82)
Deaths	Asia	60 to 64	1999	16164.24 (14857.19to17545.28)
Deaths	Asia	65 to 69	1999	14456.27 (13278.95to15781.65)

Deaths	Asia	70 to 74	1999	11897.74 (10973.27to12895.88)
Deaths	Asia	75 to 79	1999	8650.52 (7942.27to9399.07)
Deaths	Asia	80 to 84	1999	5699.16 (4968.54to6317.64)
Deaths	Asia	85 to 89	1999	3298.12 (2748.62to3694.34)
Deaths	Asia	90 to 94	1999	1336.17 (1071.6to1537.16)
Deaths	Asia	95 plus	1999	362.72 (272.59to428.41)
Deaths	Asia	15 to 19	2000	306.05 (265.7to355.44)
Deaths	Asia	20 to 24	2000	712.8 (608.77to830.61)
Deaths	Asia	25 to 29	2000	1961.83 (1676.08to2342.07)
Deaths	Asia	30 to 34	2000	5336.71 (4636.79to6207.96)
Deaths	Asia	35 to 39	2000	10284.72 (9396.66to11266.96)
Deaths	Asia	40 to 44	2000	14663.88 (13626.61to15916.68)

Deaths	Asia	45 to 49	2000	18915.1 (17153.98to20885.78)
Deaths	Asia	50 to 54	2000	20454.51 (18659.5to22721.84)
Deaths	Asia	55 to 59	2000	19644.62 (18018.04to21325.04)
Deaths	Asia	60 to 64	2000	16433.97 (15179.2to17818.28)
Deaths	Asia	65 to 69	2000	15029.22 (13794.1to16359.31)
Deaths	Asia	70 to 74	2000	12225.57 (11285.51to13250.34)
Deaths	Asia	75 to 79	2000	9010.94 (8217.13to9833.89)
Deaths	Asia	80 to 84	2000	5904.83 (5152.89to6527.23)
Deaths	Asia	85 to 89	2000	3456.73 (2911.42to3903.54)
Deaths	Asia	90 to 94	2000	1425.12 (1144.07to1647.13)
Deaths	Asia	95 plus	2000	392.17 (291.51to463.47)
Deaths	Asia	15 to 19	2003	324.7 (282.41to371.79)

Deaths	Asia	20 to 24	2003	748.43 (664.9to840.3)
Deaths	Asia	25 to 29	2003	1983.3 (1781.26to2226.47)
Deaths	Asia	30 to 34	2003	5537.63 (5037.34to6107.1)
Deaths	Asia	35 to 39	2003	11166.68 (10277.46to12177.14)
Deaths	Asia	40 to 44	2003	15498.72 (14392.72to16622.69)
Deaths	Asia	45 to 49	2003	20392.93 (18955.79to21919.18)
Deaths	Asia	50 to 54	2003	23844.07 (21742.98to26204.98)
Deaths	Asia	55 to 59	2003	21051.85 (19144.16to23304.19)
Deaths	Asia	60 to 64	2003	17130.28 (15627.25to18655.26)
Deaths	Asia	65 to 69	2003	16211.82 (14782.11to17731.02)
Deaths	Asia	70 to 74	2003	13565.42 (12410.77to14756.59)
Deaths	Asia	75 to 79	2003	9968.86 (9052.33to10755.1)

Deaths	Asia	80 to 84	2003	6682.43 (5876.31to7225.1)
Deaths	Asia	85 to 89	2003	3879.34 (3258.29to4310.28)
Deaths	Asia	90 to 94	2003	1701.76 (1385.66to1937.12)
Deaths	Asia	95 plus	2003	517.62 (380.64to605.13)
Deaths	Asia	15 to 19	2001	311.4 (270.92to359.6)
Deaths	Asia	20 to 24	2001	722.25 (627.4to827.77)
Deaths	Asia	25 to 29	2001	1964.99 (1678.03to2333.17)
Deaths	Asia	30 to 34	2001	5307.55 (4684.67to6086.02)
Deaths	Asia	35 to 39	2001	10749.9 (9856.77to11832.12)
Deaths	Asia	40 to 44	2001	14697.69 (13732.86to15835.65)
Deaths	Asia	45 to 49	2001	19331.52 (17848.94to21136.53)
Deaths	Asia	50 to 54	2001	21699.01 (19960.49to23812.83)

Deaths	Asia	55 to 59	2001	20152.14 (18554.99to21882.96)
Deaths	Asia	60 to 64	2001	16659.2 (15535.35to17904.23)
Deaths	Asia	65 to 69	2001	15565.46 (14343.08to16984.83)
Deaths	Asia	70 to 74	2001	12681.51 (11768.31to13773.59)
Deaths	Asia	75 to 79	2001	9350.42 (8492.16to10168.21)
Deaths	Asia	80 to 84	2001	6142.82 (5360.99to6766.69)
Deaths	Asia	85 to 89	2001	3623.47 (3033.78to4071.09)
Deaths	Asia	90 to 94	2001	1526.3 (1228.48to1753.74)
Deaths	Asia	95 plus	2001	432.99 (319.74to510.87)
Deaths	Asia	15 to 19	2004	330.49 (286.97to379.47)
Deaths	Asia	20 to 24	2004	761.78 (675.06to859.04)
Deaths	Asia	25 to 29	2004	1963.3 (1763.5to2200.8)

Deaths	Asia	30 to 34	2004	5503.16 (5039.57to6079.22)
Deaths	Asia	35 to 39	2004	11127.34 (10357.3to12018.54)
Deaths	Asia	40 to 44	2004	16117.37 (15075.51to17320.55)
Deaths	Asia	45 to 49	2004	20507.98 (19224.81to22075.95)
Deaths	Asia	50 to 54	2004	24567.96 (22779.33to26778.12)
Deaths	Asia	55 to 59	2004	22373.68 (20546.17to24629.09)
Deaths	Asia	60 to 64	2004	17485.77 (16269.5to18950.42)
Deaths	Asia	65 to 69	2004	16487.82 (15183.56to17836.25)
Deaths	Asia	70 to 74	2004	14059.1 (12895.83to15290.43)
Deaths	Asia	75 to 79	2004	10363.8 (9436.25to11205.48)
Deaths	Asia	80 to 84	2004	7067.93 (6210.8to7680.18)
Deaths	Asia	85 to 89	2004	4059.51 (3418.62to4483.08)

Deaths	Asia	90 to 94	2004	1809.96 (1469.94to2040.28)
Deaths	Asia	95 plus	2004	566.33 (415.69to661.84)
Deaths	Asia	15 to 19	2005	337.27 (292.71to390.35)
Deaths	Asia	20 to 24	2005	782.69 (694.62to876.11)
Deaths	Asia	25 to 29	2005	1959 (1781.29to2179.85)
Deaths	Asia	30 to 34	2005	5382.83 (4963.15to5817.85)
Deaths	Asia	35 to 39	2005	11142.58 (10418.55to11961.08)
Deaths	Asia	40 to 44	2005	16783.46 (15723.95to17832.43)
Deaths	Asia	45 to 49	2005	20378.24 (19220.97to21674.45)
Deaths	Asia	50 to 54	2005	25761.07 (24015.48to27869.32)
Deaths	Asia	55 to 59	2005	23513.32 (21786.62to25601.6)
Deaths	Asia	60 to 64	2005	17881.73 (16637.09to19385.38)

Deaths	Asia	65 to 69	2005	16800.37 (15474.73to18040.97)
Deaths	Asia	70 to 74	2005	14559.07 (13495.39to15772.66)
Deaths	Asia	75 to 79	2005	10799.3 (9865.84to11626.46)
Deaths	Asia	80 to 84	2005	7434.14 (6572.91to8002.93)
Deaths	Asia	85 to 89	2005	4274.83 (3601.73to4740.29)
Deaths	Asia	90 to 94	2005	1920.57 (1556.25to2156.47)
Deaths	Asia	95 plus	2005	609.62 (450.52to709.49)
Deaths	Asia	15 to 19	2006	336.45 (289.88to391.17)
Deaths	Asia	20 to 24	2006	800.56 (713.93to894.25)
Deaths	Asia	25 to 29	2006	1966.91 (1793.91to2174.91)
Deaths	Asia	30 to 34	2006	5290.4 (4893.74to5705)
Deaths	Asia	35 to 39	2006	11161.98 (10424.86to12051.51)

Deaths	Asia	40 to 44	2006	17566.01 (16445.1to18923.64)
Deaths	Asia	45 to 49	2006	20221.49 (19120.22to21372.29)
Deaths	Asia	50 to 54	2006	26319.13 (24532.51to28386.44)
Deaths	Asia	55 to 59	2006	25002.8 (23133.84to27036.39)
Deaths	Asia	60 to 64	2006	18527.03 (17213.03to19956.41)
Deaths	Asia	65 to 69	2006	17066.7 (15802.34to18251.08)
Deaths	Asia	70 to 74	2006	15143.81 (13975.9to16274.28)
Deaths	Asia	75 to 79	2006	11141.23 (10195.34to11894.19)
Deaths	Asia	80 to 84	2006	7801.99 (6820.82to8484.84)
Deaths	Asia	85 to 89	2006	4459.77 (3773.36to4963.15)
Deaths	Asia	90 to 94	2006	2029.76 (1638.42to2293.59)
Deaths	Asia	95 plus	2006	673.4 (494.87to785.7)

Deaths	Asia	15 to 19	2007	336.34 (290.14to390.19)
Deaths	Asia	20 to 24	2007	823.96 (741.4to919.09)
Deaths	Asia	25 to 29	2007	2004.68 (1833.58to2223.14)
Deaths	Asia	30 to 34	2007	5268.42 (4905.16to5672.29)
Deaths	Asia	35 to 39	2007	11136.66 (10400.73to11929.96)
Deaths	Asia	40 to 44	2007	18264.34 (16919.37to19768.86)
Deaths	Asia	45 to 49	2007	20537.59 (19443.12to21784.11)
Deaths	Asia	50 to 54	2007	26767.05 (25133.66to28775.93)
Deaths	Asia	55 to 59	2007	26212.62 (24400.86to28126.98)
Deaths	Asia	60 to 64	2007	19493.89 (18269.79to20896.84)
Deaths	Asia	65 to 69	2007	17356.6 (16226.53to18461.7)
Deaths	Asia	70 to 74	2007	15763.65 (14634.18to16945.39)

Deaths	Asia	75 to 79	2007	11585.52 (10647.25to12376.5)
Deaths	Asia	80 to 84	2007	8252.22 (7188.48to9023.36)
Deaths	Asia	85 to 89	2007	4753.15 (4025.86to5309.57)
Deaths	Asia	90 to 94	2007	2153.51 (1729.85to2425.34)
Deaths	Asia	95 plus	2007	746.48 (549.12to872.19)
Deaths	Asia	15 to 19	2008	342.61 (292.16to404.32)
Deaths	Asia	20 to 24	2008	847.46 (757.17to949.87)
Deaths	Asia	25 to 29	2008	2086.75 (1891.71to2306.63)
Deaths	Asia	30 to 34	2008	5312.22 (4920.16to5742.59)
Deaths	Asia	35 to 39	2008	11077.23 (10336.99to11879.24)
Deaths	Asia	40 to 44	2008	18462.69 (17132.16to19979.47)
Deaths	Asia	45 to 49	2008	21534.28 (20339.58to22748.88)

Deaths	Asia	50 to 54	2008	26732.34 (25066.59to28559.13)
Deaths	Asia	55 to 59	2008	27376.72 (25686.56to29385.26)
Deaths	Asia	60 to 64	2008	20477.73 (19117.25to21998.93)
Deaths	Asia	65 to 69	2008	17757.87 (16498.78to18882.33)
Deaths	Asia	70 to 74	2008	16257.69 (15128.18to17385.27)
Deaths	Asia	75 to 79	2008	12197.7 (11189.15to13112.68)
Deaths	Asia	80 to 84	2008	8708.06 (7596.33to9397.13)
Deaths	Asia	85 to 89	2008	5034.89 (4197.07to5538.64)
Deaths	Asia	90 to 94	2008	2241.66 (1790.92to2517.36)
Deaths	Asia	95 plus	2008	795.72 (580.72to925.64)
Deaths	Asia	15 to 19	2009	347.77 (292.54to411.67)
Deaths	Asia	20 to 24	2009	872.61 (778.91to971.94)

Deaths	Asia	25 to 29	2009	2211.12 (1977.74to2459.5)
Deaths	Asia	30 to 34	2009	5413.12 (5044.6to5846.48)
Deaths	Asia	35 to 39	2009	11093.67 (10389.19to11912.32)
Deaths	Asia	40 to 44	2009	18342.24 (17055.86to19583.83)
Deaths	Asia	45 to 49	2009	22957.17 (21581.11to24499.04)
Deaths	Asia	50 to 54	2009	26659.18 (24953.52to28331.89)
Deaths	Asia	55 to 59	2009	27960.52 (26212.37to29717.95)
Deaths	Asia	60 to 64	2009	21820.96 (20472.59to23406.27)
Deaths	Asia	65 to 69	2009	18491.07 (17291.55to19661.42)
Deaths	Asia	70 to 74	2009	16672.09 (15572.64to17844.65)
Deaths	Asia	75 to 79	2009	12799.13 (11736.86to13768.53)
Deaths	Asia	80 to 84	2009	9069.24 (7924.99to9805.6)

Deaths	Asia	85 to 89	2009	5257.66 (4402.08to5783.68)
Deaths	Asia	90 to 94	2009	2300.64 (1829.14to2577.67)
Deaths	Asia	95 plus	2009	848.05 (613.7to985.34)
Deaths	Asia	15 to 19	2010	353.38 (295.58to414.85)
Deaths	Asia	20 to 24	2010	902.68 (805.41to1003.8)
Deaths	Asia	25 to 29	2010	2304.59 (2071.61to2546.55)
Deaths	Asia	30 to 34	2010	5510.03 (5103.55to5921.29)
Deaths	Asia	35 to 39	2010	10985.78 (10351.21to11627.15)
Deaths	Asia	40 to 44	2010	18401.59 (17334.83to19599.46)
Deaths	Asia	45 to 49	2010	24429.12 (22963.9to26051.48)
Deaths	Asia	50 to 54	2010	26984.47 (25400.2to28696.87)
Deaths	Asia	55 to 59	2010	29398.96 (27653.8to31154.83)

Deaths	Asia	60 to 64	2010	23153.11 (21757.72to24646.29)
Deaths	Asia	65 to 69	2010	19245.12 (18047.08to20332.18)
Deaths	Asia	70 to 74	2010	17165.77 (16049.99to18205.26)
Deaths	Asia	75 to 79	2010	13422.47 (12368.59to14360.54)
Deaths	Asia	80 to 84	2010	9561.08 (8345.06to10390.93)
Deaths	Asia	85 to 89	2010	5589.01 (4552.17to6196.99)
Deaths	Asia	90 to 94	2010	2478.54 (1944.31to2803.16)
Deaths	Asia	95 plus	2010	935.63 (673.96to1089.28)
Deaths	Asia	15 to 19	2011	362.35 (304.57to433.59)
Deaths	Asia	20 to 24	2011	930.75 (824.12to1035.72)
Deaths	Asia	25 to 29	2011	2371.66 (2132.28to2624.21)
Deaths	Asia	30 to 34	2011	5580.16 (5138.76to6037)

Deaths	Asia	35 to 39	2011	10888.55 (10212.39to11635.66)
Deaths	Asia	40 to 44	2011	18637.21 (17462.12to19746.6)
Deaths	Asia	45 to 49	2011	25220.72 (23631.18to27076.58)
Deaths	Asia	50 to 54	2011	27449.41 (25633.49to29182.18)
Deaths	Asia	55 to 59	2011	30210.99 (28174.53to32358.39)
Deaths	Asia	60 to 64	2011	24873.4 (23293.9to26435.3)
Deaths	Asia	65 to 69	2011	19941.58 (18638.7to21297.08)
Deaths	Asia	70 to 74	2011	17620.29 (16382.25to18827.31)
Deaths	Asia	75 to 79	2011	13996.91 (12785.57to15113.88)
Deaths	Asia	80 to 84	2011	10285.4 (8966.69to11173.3)
Deaths	Asia	85 to 89	2011	6018.31 (4984.42to6725.48)
Deaths	Asia	90 to 94	2011	2694.15 (2131.64to3052.32)

Deaths	Asia	95 plus	2011	1051.56 (760.06to1226.65)
Deaths	Asia	15 to 19	2012	362.73 (295.54to438.8)
Deaths	Asia	20 to 24	2012	946.88 (847.04to1051.57)
Deaths	Asia	25 to 29	2012	2439.21 (2208.81to2708.75)
Deaths	Asia	30 to 34	2012	5620.7 (5146.16to6086.57)
Deaths	Asia	35 to 39	2012	11002.36 (10330.67to11715.02)
Deaths	Asia	40 to 44	2012	18791.65 (17575.3to20168.43)
Deaths	Asia	45 to 49	2012	25477.21 (23729.09to27377.9)
Deaths	Asia	50 to 54	2012	28241.42 (26326.46to30205.41)
Deaths	Asia	55 to 59	2012	30948.44 (28704to33640.92)
Deaths	Asia	60 to 64	2012	25982.16 (24170.89to27924.44)
Deaths	Asia	65 to 69	2012	20732.36 (19219.87to22326.47)

Deaths	Asia	70 to 74	2012	17980.95 (16712.34to19249.55)
Deaths	Asia	75 to 79	2012	14348.61 (13037.1to15394.83)
Deaths	Asia	80 to 84	2012	10653.49 (9284.46to11558.18)
Deaths	Asia	85 to 89	2012	6337.63 (5179.65to7036.67)
Deaths	Asia	90 to 94	2012	2894.37 (2255.69to3287.49)
Deaths	Asia	95 plus	2012	1144.48 (821.29to1331.78)
Deaths	Asia	15 to 19	2013	372.09 (301.45to453.86)
Deaths	Asia	20 to 24	2013	962.57 (841.07to1098.99)
Deaths	Asia	25 to 29	2013	2520.55 (2268.35to2841)
Deaths	Asia	30 to 34	2013	5769.73 (5313.05to6323.06)
Deaths	Asia	35 to 39	2013	11230.47 (10508.32to12003.19)
Deaths	Asia	40 to 44	2013	19263.36 (17936.67to20767.98)

Deaths	Asia	45 to 49	2013	26185.66 (24277.02to28204.01)
Deaths	Asia	50 to 54	2013	29748.11 (27377.87to32494.75)
Deaths	Asia	55 to 59	2013	31650.22 (29320.28to33997.38)
Deaths	Asia	60 to 64	2013	27241.9 (25329.73to29300.35)
Deaths	Asia	65 to 69	2013	21479.7 (19852.67to23090.56)
Deaths	Asia	70 to 74	2013	18378.33 (16922.4to19578.98)
Deaths	Asia	75 to 79	2013	14848.41 (13579.45to15885.38)
Deaths	Asia	80 to 84	2013	10745.24 (9294.63to11943.86)
Deaths	Asia	85 to 89	2013	6607.08 (5385.37to7429.54)
Deaths	Asia	90 to 94	2013	3158.02 (2455.46to3603.06)
Deaths	Asia	95 plus	2013	1258.26 (897.49to1460.34)
Deaths	Asia	15 to 19	2014	376.15 (299.87to464.31)

Deaths	Asia	20 to 24	2014	960.41 (795.33to1132.17)
Deaths	Asia	25 to 29	2014	2519.3 (2265.34to2827.84)
Deaths	Asia	30 to 34	2014	5878.18 (5408.78to6413.68)
Deaths	Asia	35 to 39	2014	11294.74 (10604.62to12080.76)
Deaths	Asia	40 to 44	2014	19722.75 (18320.87to21197.19)
Deaths	Asia	45 to 49	2014	26534.66 (24597.08to28666.98)
Deaths	Asia	50 to 54	2014	31647.63 (28523.51to35298.99)
Deaths	Asia	55 to 59	2014	32567.32 (30234.9to35002.18)
Deaths	Asia	60 to 64	2014	28084.98 (25880.56to30304.06)
Deaths	Asia	65 to 69	2014	22345.67 (20732.84to24024.06)
Deaths	Asia	70 to 74	2014	18730.65 (17241.74to20024.41)
Deaths	Asia	75 to 79	2014	15273.72 (13915.83to16424.38)

Deaths	Asia	80 to 84	2014	11123.43 (9450.77to12577.66)
Deaths	Asia	85 to 89	2014	6929.45 (5622.96to7947.42)
Deaths	Asia	90 to 94	2014	3409.87 (2646.09to3948.2)
Deaths	Asia	95 plus	2014	1331.56 (958.16to1548.64)
Deaths	Asia	15 to 19	2015	376.99 (295.01to471.13)
Deaths	Asia	20 to 24	2015	968.04 (781.66to1176.38)
Deaths	Asia	25 to 29	2015	2533.5 (2243.71to2887.29)
Deaths	Asia	30 to 34	2015	6023.84 (5468.74to6589.63)
Deaths	Asia	35 to 39	2015	11499.74 (10658.54to12364.75)
Deaths	Asia	40 to 44	2015	20110.93 (18457.96to21702.36)
Deaths	Asia	45 to 49	2015	26915.64 (24581.54to29246.61)
Deaths	Asia	50 to 54	2015	33779.13 (29672to38180.23)

Deaths	Asia	55 to 59	2015	33632.1 (30669.29to36495.06)
Deaths	Asia	60 to 64	2015	29720.66 (27180.44to32135.93)
Deaths	Asia	65 to 69	2015	23587.85 (21456.24to25437.84)
Deaths	Asia	70 to 74	2015	19309.84 (17676.06to20705.39)
Deaths	Asia	75 to 79	2015	15684.85 (14109.59to16971.92)
Deaths	Asia	80 to 84	2015	11772.65 (10017.75to13192.89)
Deaths	Asia	85 to 89	2015	7435.96 (5976.48to8371.61)
Deaths	Asia	90 to 94	2015	3673.51 (2810.25to4197.23)
Deaths	Asia	95 plus	2015	1441.29 (1028.51to1685.7)
Deaths	Asia	15 to 19	2016	380.06 (295.76to486.18)
Deaths	Asia	20 to 24	2016	987 (789.92to1212.7)
Deaths	Asia	25 to 29	2016	2603.37 (2276.33to2987.51)

Deaths	Asia	30 to 34	2016	6269.23 (5703.88to6931.43)
Deaths	Asia	35 to 39	2016	11841.96 (11002.75to12727.71)
Deaths	Asia	40 to 44	2016	20409.16 (18989.78to22247.07)
Deaths	Asia	45 to 49	2016	27682.34 (25539.02to30142.32)
Deaths	Asia	50 to 54	2016	35415.61 (31490.69to40116.02)
Deaths	Asia	55 to 59	2016	34551.35 (31742.57to37647.28)
Deaths	Asia	60 to 64	2016	30822.72 (28373.55to33614.93)
Deaths	Asia	65 to 69	2016	25474.79 (23530.76to27830.51)
Deaths	Asia	70 to 74	2016	20206.1 (18698.09to21912.54)
Deaths	Asia	75 to 79	2016	16141.63 (14504.05to17543.73)
Deaths	Asia	80 to 84	2016	12521.19 (10844.29to13889.81)
Deaths	Asia	85 to 89	2016	7957.2 (6434.55to8885.47)

Deaths	Asia	90 to 94	2016	4025.91 (3094.77to4614.32)
Deaths	Asia	95 plus	2016	1617.53 (1140.94to1886.67)
Deaths	Asia	15 to 19	2018	385.74 (289.3to508.85)
Deaths	Asia	20 to 24	2018	1005.87 (793.72to1257.41)
Deaths	Asia	25 to 29	2018	2649.21 (2260.17to3117.84)
Deaths	Asia	30 to 34	2018	6700.21 (5980.51to7487.85)
Deaths	Asia	35 to 39	2018	12288.79 (11395.09to13342.7)
Deaths	Asia	40 to 44	2018	20655.36 (18913.97to22592.06)
Deaths	Asia	45 to 49	2018	29265.22 (26605.17to32164.82)
Deaths	Asia	50 to 54	2018	38256.79 (34142.14to42475.16)
Deaths	Asia	55 to 59	2018	38128.14 (34013.94to42569.77)
Deaths	Asia	60 to 64	2018	33169.47 (30197.1to36131.09)

Deaths	Asia	65 to 69	2018	29468.6 (26774.88to32305.57)
Deaths	Asia	70 to 74	2018	22850.23 (20805.18to24836.39)
Deaths	Asia	75 to 79	2018	17530.31 (15705.65to19118.13)
Deaths	Asia	80 to 84	2018	13548.68 (11568.81to15199.31)
Deaths	Asia	85 to 89	2018	8652.76 (6953.93to9748.06)
Deaths	Asia	90 to 94	2018	4595.02 (3489.1to5252.05)
Deaths	Asia	95 plus	2018	1971.4 (1389.26to2323.39)
Deaths	Asia	15 to 19	2017	388.04 (298.78to492.8)
Deaths	Asia	20 to 24	2017	1002.05 (800.61to1219.68)
Deaths	Asia	25 to 29	2017	2670.53 (2308.06to3050.14)
Deaths	Asia	30 to 34	2017	6566.11 (5943.18to7246.5)
Deaths	Asia	35 to 39	2017	12177.23 (11283.33to13097.13)

Deaths	Asia	40 to 44	2017	20605.39 (18686.5to22445.2)
Deaths	Asia	45 to 49	2017	28588.05 (25890.84to31293.85)
Deaths	Asia	50 to 54	2017	37113.73 (33211.97to41726.75)
Deaths	Asia	55 to 59	2017	35867.07 (32543.31to39786.64)
Deaths	Asia	60 to 64	2017	32139.3 (29188to35315.71)
Deaths	Asia	65 to 69	2017	27525.7 (25223.51to30111.61)
Deaths	Asia	70 to 74	2017	21541.48 (19814.8to23448.97)
Deaths	Asia	75 to 79	2017	16808.75 (15135.02to18417.99)
Deaths	Asia	80 to 84	2017	12974.96 (10983.66to14691.86)
Deaths	Asia	85 to 89	2017	8219.99 (6659.63to9316.59)
Deaths	Asia	90 to 94	2017	4285.67 (3266.78to4927.76)
Deaths	Asia	95 plus	2017	1786.2 (1263.93to2099)

Deaths	Asia	15 to 19	2019	391.7 (292.66to521.62)
Deaths	Asia	20 to 24	2019	1022.67 (794.1to1297.73)
Deaths	Asia	25 to 29	2019	2682.36 (2301.69to3154.68)
Deaths	Asia	30 to 34	2019	6855.97 (6069.97to7732.68)
Deaths	Asia	35 to 39	2019	12543.52 (11533.72to13706.22)
Deaths	Asia	40 to 44	2019	20658.72 (18818.56to22759.32)
Deaths	Asia	45 to 49	2019	29888.14 (26943.09to33091.72)
Deaths	Asia	50 to 54	2019	39038.3 (34870.07to43397.94)
Deaths	Asia	55 to 59	2019	40551.81 (35551.93to46166.89)
Deaths	Asia	60 to 64	2019	33801.07 (30747.76to37001.13)
Deaths	Asia	65 to 69	2019	30909.57 (27831to34219.02)
Deaths	Asia	70 to 74	2019	24430.21 (22196.31to26695.45)

Deaths	Asia	75 to 79	2019	18264.29 (16120.83to20321.47)
Deaths	Asia	80 to 84	2019	14213.51 (12131.59to15865.15)
Deaths	Asia	85 to 89	2019	9233.79 (7387.62to10397.94)
Deaths	Asia	90 to 94	2019	5013.6 (3803.14to5761.95)
Deaths	Asia	95 plus	2019	2134.53 (1508.84to2520.45)
Deaths	Asia	15 to 19	2021	418.22 (307.95to542.49)
Deaths	Asia	20 to 24	2021	1072.22 (850.91to1369.04)
Deaths	Asia	25 to 29	2021	2699.11 (2289.2to3189.6)
Deaths	Asia	30 to 34	2021	7178.87 (6337.22to8139.51)
Deaths	Asia	35 to 39	2021	13252.83 (11934.91to14714.22)
Deaths	Asia	40 to 44	2021	21213.72 (18862.31to23653.69)
Deaths	Asia	45 to 49	2021	30373.19 (27103.79to33853.46)

Deaths	Asia	50 to 54	2021	40076.43 (35515.79to45132.3)
Deaths	Asia	55 to 59	2021	44131.81 (37991.78to50971.79)
Deaths	Asia	60 to 64	2021	33973.14 (30382.73to37961.73)
Deaths	Asia	65 to 69	2021	33234.6 (29872.49to37140.62)
Deaths	Asia	70 to 74	2021	27322.24 (24632.02to30436.39)
Deaths	Asia	75 to 79	2021	19048 (16664.11to21317.32)
Deaths	Asia	80 to 84	2021	14810.59 (12417.32to16804.21)
Deaths	Asia	85 to 89	2021	9870.54 (7800.67to11396.85)
Deaths	Asia	90 to 94	2021	5421.13 (4072.08to6287.22)
Deaths	Asia	95 plus	2021	2397.94 (1653.98to2857.22)
Deaths	Asia	15 to 19	1996	261.63 (226.48to298.64)
Deaths	Asia	20 to 24	1996	639.67 (540.74to759.53)

Deaths	Asia	25 to 29	1996	1801.21 (1533.87to2158.88)
Deaths	Asia	30 to 34	1996	4783.1 (4178.48to5583.18)
Deaths	Asia	35 to 39	1996	8653.49 (7893.33to9475.41)
Deaths	Asia	40 to 44	1996	14154.62 (12875.1to15526.83)
Deaths	Asia	45 to 49	1996	16179.07 (14882.78to17710.63)
Deaths	Asia	50 to 54	1996	17199.66 (15636.72to19068.22)
Deaths	Asia	55 to 59	1996	17850.96 (16261.45to19864.77)
Deaths	Asia	60 to 64	1996	14924.53 (13614.42to16718.84)
Deaths	Asia	65 to 69	1996	12881.3 (11872.09to13994.34)
Deaths	Asia	70 to 74	1996	10488.67 (9623.5to11393.92)
Deaths	Asia	75 to 79	1996	7592.53 (6960.71to8262.84)
Deaths	Asia	80 to 84	1996	5193.99 (4581.24to5714.27)

Deaths	Asia	85 to 89	1996	2895.81 (2449.97to3248.76)
Deaths	Asia	90 to 94	1996	1109.86 (884.44to1277.49)
Deaths	Asia	95 plus	1996	285.81 (216.35to340.13)
Deaths	Asia	15 to 19	2002	315.83 (276.36to366.19)
Deaths	Asia	20 to 24	2002	733.16 (643.16to837.21)
Deaths	Asia	25 to 29	2002	1967.69 (1709.7to2291.93)
Deaths	Asia	30 to 34	2002	5370.32 (4816.36to6035.15)
Deaths	Asia	35 to 39	2002	11092.44 (10149.53to12257.96)
Deaths	Asia	40 to 44	2002	14973.31 (13944.46to16145.65)
Deaths	Asia	45 to 49	2002	19869.12 (18466.29to21614.6)
Deaths	Asia	50 to 54	2002	22743.92 (20976.33to24967.46)
Deaths	Asia	55 to 59	2002	20569.65 (18925.03to22564.25)

Deaths	Asia	60 to 64	2002	16848.48 (15593.13to18425.77)
Deaths	Asia	65 to 69	2002	15891.55 (14549.09to17294.9)
Deaths	Asia	70 to 74	2002	13123.04 (12121.97to14248.52)
Deaths	Asia	75 to 79	2002	9662.06 (8771.28to10405.97)
Deaths	Asia	80 to 84	2002	6375.73 (5559.93to6951.79)
Deaths	Asia	85 to 89	2002	3737.68 (3127.49to4149.6)
Deaths	Asia	90 to 94	2002	1615.56 (1306.48to1853.18)
Deaths	Asia	95 plus	2002	475.68 (346.97to560.02)
Deaths	Asia	15 to 19	1993	240.94 (208.56to276.9)
Deaths	Asia	20 to 24	1993	596.13 (505.72to715.45)
Deaths	Asia	25 to 29	1993	1685.41 (1422.95to2033.27)
Deaths	Asia	30 to 34	1993	4122.38 (3573.3to4849.1)

Deaths	Asia	35 to 39	1993	8413.05 (7494.23to9522.27)
Deaths	Asia	40 to 44	1993	12552.05 (11378.18to13897.61)
Deaths	Asia	45 to 49	1993	13905.3 (12770.55to15241.62)
Deaths	Asia	50 to 54	1993	15830.31 (14100.38to17797.65)
Deaths	Asia	55 to 59	1993	16819.61 (15043.01to18993.84)
Deaths	Asia	60 to 64	1993	13495.9 (12111.05to15066.95)
Deaths	Asia	65 to 69	1993	11330.91 (10284.02to12583.78)
Deaths	Asia	70 to 74	1993	9015.92 (8118.62to9992.73)
Deaths	Asia	75 to 79	1993	6533 (5896.99to7278.67)
Deaths	Asia	80 to 84	1993	4329.57 (3810.84to4776.62)
Deaths	Asia	85 to 89	1993	2324.53 (1963.56to2607.16)
Deaths	Asia	90 to 94	1993	819.04 (675.67to931.96)

Deaths	Asia	95 plus	1993	213.95 (166.51to252.25)
Deaths	Asia	15 to 19	2020	413.9 (300.67to555.1)
Deaths	Asia	20 to 24	2020	1064.28 (817.79to1346.66)
Deaths	Asia	25 to 29	2020	2696.69 (2288.28to3215.13)
Deaths	Asia	30 to 34	2020	7126.12 (6165.98to8108.38)
Deaths	Asia	35 to 39	2020	12950.43 (11659.8to14189.2)
Deaths	Asia	40 to 44	2020	20926.95 (18952.93to23191.46)
Deaths	Asia	45 to 49	2020	30222.97 (27274.38to33292.48)
Deaths	Asia	50 to 54	2020	39699.48 (35584.46to44273.49)
Deaths	Asia	55 to 59	2020	42083.87 (36709.43to48216.78)
Deaths	Asia	60 to 64	2020	33624.03 (30310.52to37105.68)
Deaths	Asia	65 to 69	2020	32325.04 (29093.46to35803.27)

Deaths	Asia	70 to 74	2020	25654.99 (23257.92to28249.02)
Deaths	Asia	75 to 79	2020	18401.86 (16094.06to20651.5)
Deaths	Asia	80 to 84	2020	14504.26 (12402.89to16204.2)
Deaths	Asia	85 to 89	2020	9423.69 (7509.02to10705.71)
Deaths	Asia	90 to 94	2020	5131.2 (3874.25to5945.51)
Deaths	Asia	95 plus	2020	2211.96 (1532.52to2619.44)

Supplementary Table S4. Absolute and relative cross-country inequality in ASDR of Asian breast cancer, 1990–2021.

Year	Lower	Slope Index of Inequality (SII)	Upper
1990	76.29014427	239.6962167	403.1022891
1991	83.04368452	223.0032173	362.96275
1992	96.14995257	235.3614962	374.5730398
1993	99.09201341	259.2235922	419.355171
1994	134.772935	270.3382403	405.9035457

1995	123.0960226	293.7728295	464.4496364
1996	119.6906142	310.9344569	502.1782995
1997	138.8149771	326.8828574	514.9507377
1998	202.8849834	366.1320025	529.3790215
1999	211.4104678	395.3932194	579.375971
2000	164.9106549	398.1545568	631.3984588
2001	232.3807209	420.5820905	608.7834601
2002	206.8625518	402.7759055	598.6892592
2003	216.233938	424.5899927	632.9460474
2004	225.3739717	432.2457473	639.1175228
2005	230.1576328	439.2572863	648.3569398
2006	226.6140036	445.2992503	663.984497
2007	208.5112172	441.549778	674.5883388
2008	249.4359848	458.9179178	668.3998508
2009	251.0778681	461.0301295	670.9823909
2010	304.896323	513.8760294	722.8557357
2011	309.2648796	517.2337708	725.2026621

2012	311.0837208	542.6239763	774.1642317
2013	297.1296164	533.6860808	770.2425452
2014	309.1517926	545.5430972	781.9344018
2015	287.7894139	520.8231966	753.8569793
2016	308.2455555	529.8406986	751.4358417
2017	308.1274355	526.9988755	745.8703155
2018	314.4558936	518.4331946	722.4104956
2019	293.1265645	533.4088195	773.6910744
2020	225.6033673	496.1557633	766.7081593
2021	112.5639676	422.5093527	732.4547378

Supplementary Table S5. Frontier analysis on the basis of sociodemographic-index and ASDR (per 100,000) of breast cancer in Asia in 2021.

location	year	val	SDI	frontier	eff_diff	trend
Armenia	2021	559.9601853	0.701833194	183.2383256	376.7218597	Decrease
Azerbaijan	2021	459.2150125	0.694851274	183.2383256	275.9766869	Decrease
Bangladesh	2021	281.9449665	0.492420885	183.2383256	98.7066409	Increase

Bhutan	2021	267.9064647	0.473062378	183.2383256	84.6681391	Increase
Brunei Darussalam	2021	618.0768186	0.810234367	183.2383256	434.838493	Increase
Cambodia	2021	633.7422074	0.473621491	183.2383256	450.5038818	Increase
China	2021	281.5417975	0.72162976	183.2383256	98.3034719	Decrease
Democratic People's Republic of Korea	2021	381.1333924	0.569854634	183.2383256	197.8950668	Increase
Georgia	2021	835.3615795	0.789518937	183.2383256	652.1232539	Decrease
India	2021	401.8103072	0.575401649	183.2383256	218.5719816	Increase
Indonesia	2021	604.2516824	0.656868336	183.2383256	421.0133568	Increase
Japan	2021	352.9725508	0.871241813	183.2383256	169.7342252	Increase
Kazakhstan	2021	402.3946372	0.725144495	183.2383256	219.1563116	Decrease
Kyrgyzstan	2021	362.7340773	0.603979328	183.2383256	179.4957517	Decrease

Lao People's Democratic Republic	2021	576.1481882	0.489136091	183.2383256	392.9098626	Increase
Malaysia	2021	846.5065978	0.742523828	183.2383256	663.2682722	Increase
Maldives	2021	259.0261239	0.650886627	183.2383256	75.7877983	Decrease
Mauritius	2021	725.7737571	0.718260446	183.2383256	542.5354315	Increase
Mongolia	2021	185.223109	0.617621565	183.2383256	1.9847834	Increase
Myanmar	2021	611.4838507	0.53390084	183.2383256	428.2455251	Decrease
Nepal	2021	277.4924855	0.433174635	219.7906642	57.7018213	Increase
Pakistan	2021	927.3736462	0.504028689	183.2383256	744.1353206	Increase
Philippines	2021	778.768066	0.651219329	183.2383256	595.5297404	Increase
Republic of Korea	2021	234.9757142	0.886675267	183.2383256	51.7373886	Increase
Seychelles	2021	682.8469233	0.730150775	183.2383256	499.6085977	Increase
Singapore	2021	355.8285084	0.856097766	183.2383256	172.5901828	Decrease

Sri Lanka	2021	364.9028208	0.701534935	183.2383256	181.6644952	Increase
Taiwan (Province of China)	2021	446.6002971	0.874747053	183.2383256	263.3619715	Increase
Tajikistan	2021	353.1318489	0.541511187	183.2383256	169.8935233	Decrease
Thailand	2021	535.5460626	0.682547933	183.2383256	352.307737	Increase
Timor-Leste	2021	429.2218566	0.444667619	219.7906642	209.4311924	Increase
Turkmenistan	2021	436.2833932	0.682160776	183.2383256	253.0450676	Decrease
Uzbekistan	2021	388.8624784	0.662621694	183.2383256	205.6241528	Decrease
Viet Nam	2021	351.1408723	0.627933721	183.2383256	167.9025467	Increase
